# Copper oxide nanoparticles: from EU-regulatory landscape and mechanistic toxicity towards a cuproptosis-linked adverse outcome pathway

**DOI:** 10.1007/s00204-026-04465-1

**Published:** 2026-06-27

**Authors:** Sophie Scholz, Julia Prinz, Linde Sevenants, Andrea Holzwarth, Birgit Mertens, Robert Pieper, Vera Ritz, Kirsten Schulz, Holger Sieg, Aswin Mangerich, Linda Böhmert

**Affiliations:** 1 German Federal Institute for Risk Assessment, Max-Dohrn-Str. 8-10, 10589 Berlin, Germany; 2 Institute of Nutritional Science, Nutritional Toxicology Group, University of Potsdam, Karl-Liebknecht-Str. 24-25, 14476 Potsdam, Germany; 3 Sciensano (Belgian Institute for Health), J. Wytsmanstraat 14, 1050 Brussels, Belgium

**Keywords:** Copper oxides, Uptake mechanism, Cuproptosis, Nanotoxicology, AOP

## Abstract

Copper is an essential trace element, yet concerns regarding its toxic potential have gained increasing attention, particularly with regard to nanoparticulate forms of copper oxides. Both CuO and Cu_2_O are currently authorised in the EU for use in agriculture, biocides, and animal nutrition, but regulatory clarity regarding their nanoforms remains limited. This comprehensive review—combining narrative, bibliometric, and systematic approaches—addressed three key questions: (i) whether CuO and Cu_2_O nanoparticles are sufficiently considered within EU regulations to prevent unintentional oral exposure via the food and feed chain, (ii) whether these nanoparticles can cross the intestinal barrier as intact particles and accumulate in tissues, and (iii) whether they elicit distinct biological responses, including regulated cell death pathways such as cuproptosis. With respect to question (i), a detailed analysis of EU legal frameworks revealed a heterogeneous regulatory landscape and a lack of binding provisions that would preclude unintended presence of copper oxide nanoforms in regulated materials. Although some nano-specific data requirements exist, empirical information on nanoparticulate fractions in commercial products is still lacking. With respect to questions (ii) and (iii), our systematic literature analysis supports consistent intracellular uptake of CuO and Cu_2_O nanoparticles, yet direct evidence for transport of intact particles across the intestinal barrier is scarce. Mechanistic evidence supports cuproptosis as a key pathway for CuO nanoparticle toxicity, while the extent to which these effects differ quantitatively from ionic copper remains unclear due to insufficient data using appropriate experimental controls. Notably, a pronounced data gap was identified for Cu_2_O nanoparticles across all evaluated domains. Finally, to organise and integrate the mechanistic evidence presented, we developed an adverse outcome pathway (AOP), registered as AOP 590 in the AOP Wiki, describing how increased intracellular copper can induce cuproptosis via disruption of energy metabolism. Collectively, these findings highlight the need for coordinated analytical, mechanistic, and regulatory research to support evidence-based risk assessment of CuO and Cu_2_O nanoparticles in the food and feed context.

**Supplementary Information:**

The online version contains supplementary material available at https://doi.org/10.1007/s00204-026-04465-1.

## Objectives of the review article

Copper is an essential trace element, but its potential for toxicity in nanoparticulate form is receiving increasing attention (reviewed by Sajjad et al. 2023 and Naz et al. 2020). Both CuO and Cu_2_O are authorised in the EU for use in agriculture, biocides and animal nutrition ((EC) 1107/2009, (EC) 528/2012, (EC) 1831/2003). (EU 2012; EC 2003, EG 2009b) However, current regulatory frameworks do not consistently distinguish between nanoforms and non-nanoforms, raising the possibility of unintentional oral exposure to CuO and Cu_2_O nanoparticles (NPs) via the food and feed chain. This leads to the first key question to be addressed in this review: (i) Are CuO and Cu_2_O NPs sufficiently addressed in EU regulations to prevent unintentional oral exposure?

Potential health risks associated with CuO and Cu_2_O NPs depend not only on exposure levels, but also on their biological behaviour. While ionic copper follows well-regulated absorption, distribution, metabolism, and excretion (ADME) pathways, NPs may alter these processes, affecting bioavailability and biodistribution. (Strauch et al. 2020) This motivates the second question of this review: (ii) Can CuO and Cu_2_O NPs cross the intestinal barrier as intact particles and accumulate in sensitive tissues?

Beyond uptake and translocation, it is essential to determine whether these NPs exert biological responses distinct from those of ionic copper. A particular focus is on cuproptosis, a recently identified copper-specific form of regulated cell death. (Tsvetkov et al. 2022) This gives rise to the third question of the article: (iii) Do CuO and Cu_2_O NPs trigger distinct biological responses, including mechanisms of regulated cell death such as cuproptosis?

This review was conducted within the EFSA-funded NAMs4NANO project, which explores the use of New Approach Methodologies (NAMs) in the risk assessment of nanomaterials. Using CuO and Cu_2_O NPs as a case study, our aim was to critically evaluate the regulatory situation, assess the quality and coverage of current scientific data, and explore mechanistic insights gained from in vitro and NAM-based approaches. We begin with an overview of the EU regulatory landscape and an overview on copper homeostasis and toxicity as well as nano-specific considerations. We then present the results of our comprehensive bibliometric and systematic analysis of the scientific literature. By addressing the key questions (i) to (iii), this review seeks to summarise and discuss evidence on nano-specific effects related to cellular uptake, intestinal translocation, and mechanisms of cell death, and to highlight knowledge gaps relevant to the risk assessment and legislation of CuO and Cu_2_O NPs in the food and feed context.

## EU regulation of copper, copper oxides, and their nanoforms

**Fig. 1:**
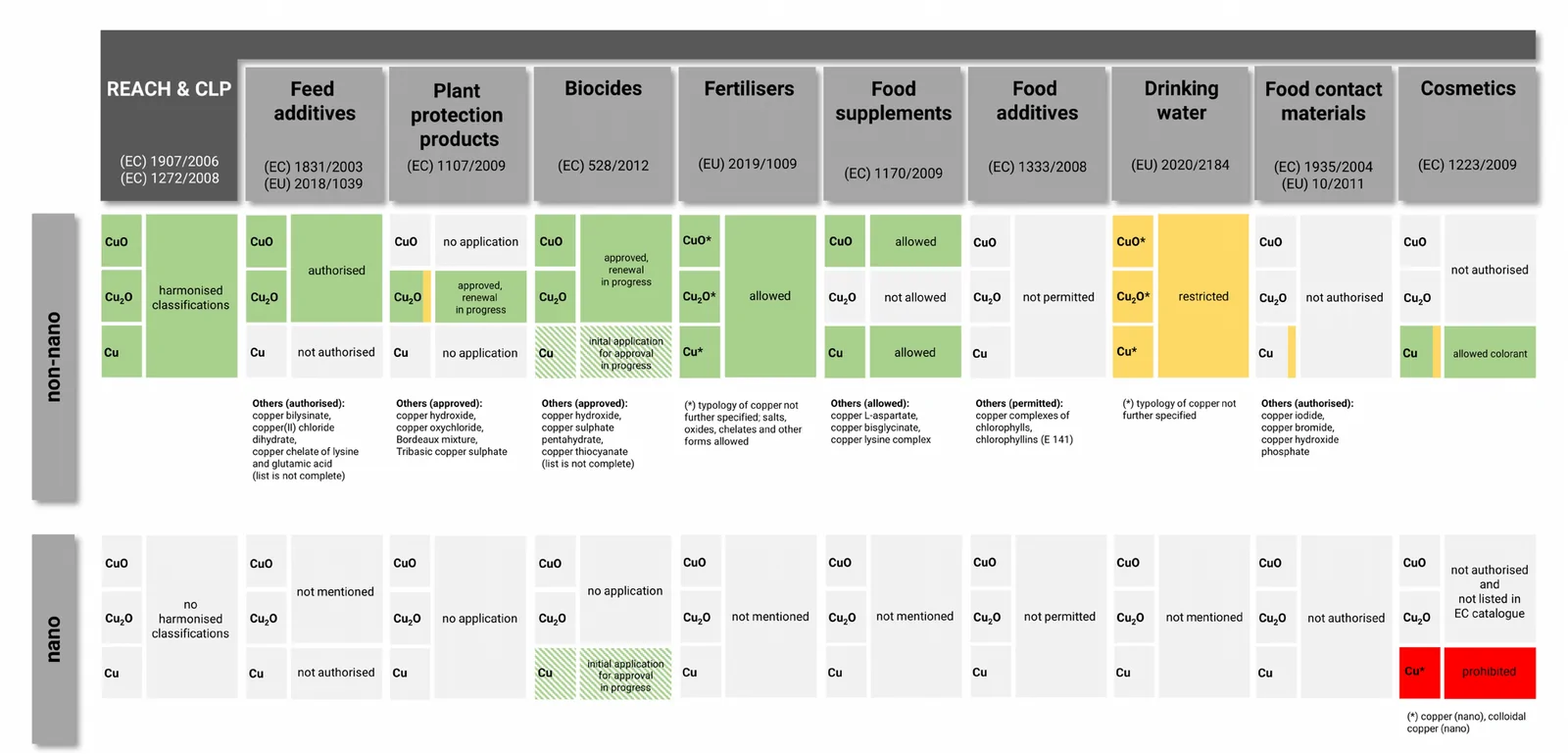
Overview of EU regulations covering different legislative sectors. Entries for CuO, Cu_2_O, copper (Cu) and other copper compounds (in both nanoforms and non-nanoforms) are summarised for each field using terminology of the respective regulation. Green indicates positive entries exist (i.e., classified, authorised, approved or allowed), grey indicates that no entries exist (i.e., not classified, not authorised, no application, not mentioned, or not permitted), orange indicates that the substance is restricted; and red indicates that the substance is prohibited. CLP: Classification, Labelling and Packaging of Chemicals and Mixtures; EC: European Commission; EU: European Union; REACH: Registration, Evaluation, Authorisation and Restriction of Chemicals

The European Union (EU) maintains multiple legislative frameworks aimed at protecting human health, animal health, and the environment from risks posed by chemicals across different sectors. This part of this review summarises key legislations relevant for copper and copper oxides, both CuO and Cu_2_O, with attention to both nanoforms and non-nanoforms (Fig. 1). By clarifying the current regulatory landscape, this section establishes the context in which scientific findings must be interpreted, as regulatory definitions, classifications, and data requirements directly influence risk assessment strategies and decision-making. Moreover, identifying regulatory gaps and ambiguities highlights where scientific evidence, particularly mechanistic insights into uptake, biodistribution, and cell death pathways, is essential to support future policy making and safety evaluation. Specifically, we review the status quo on copper and copper oxide regulations and examine whether CuO and Cu_2_O NPs are adequately addressed within EU Regulations to mitigate unintentional oral exposure through the food and feed chain.

In 2011, the European Commission (EC) released Recommendation 2011/696/EU defining nanomaterials (NMs) (2011/696/EU) (EC 2011a); this has been replaced by the Recommendation 2022/C 229/01. (EC 2022) The EC definition on NMs applies exclusively to solid particles of natural, incidental or manufactured origin. It classifies a material as a NM if at least 50% of its constituent particles are NPs. Particles are defined as NPs, if they meet at least one of the following criteria: (i) for spherical particles, at least one external dimension lies between 1 and 100 nm; (ii) for elongated particles, at least two external dimensions are below 100 nm and one dimension exceeds 100 nm; and (iii) for plate-like particles, one external dimension is below 100 nm and the remaining dimensions exceed 100 nm. A more detailed discussion of these criteria and related challenges in NPs and NMs classification and characterisation can be found elsewhere. (Rauscher et al. 2023).

Despite the EC Recommendations 2011/696/EU and 2022/C 229/01, the term NM is not applied consistently across all EU Regulations. Some legislations predate the Recommendations 2011/696/EU and 2022/C 229/01 and therefore do not align with them, and other frameworks apply their own scope and criteria, further contributing to variation in how NMs are defined. For example, in the cosmetics sector, a definition for “nanomaterials” is provided in Regulation (EC) 1223/2009, which predates the EC Recommendations 2011/696/EU and 2022/C 229/01. (EC 2009c) By contrast, the Biocidal Products Regulation (Regulation (EC) 528/2012) incorporates the EC Recommendation into its legal text. (EU 2012) Under REACH (Regulation (EC) 1907/2006), NMs have been explicitly addressed since 2020, using a modified form of the EC definition and introducing the term “nanoform” instead of NM. (EC 2006) The Novel Foods Regulation (EU) 2015/2283 includes a definition for “engineered nanomaterials”. Of note, the regulations governing plant protection products (PPP) (Regulation (EC) 1107/2009), food contact materials (FCM) (Regulation (EC) 1935/2004), and feed additives (Regulation (EC) 1831/2003) do not currently define the term “nanomaterial” (EC 2003, 2004, 2009b). In the following sections, we describe the regulatory status of copper, CuO, Cu_2_O, and other copper compounds in both nanoform and non-nanoform, across relevant EU legislative frameworks.

### REACH [(EC) 1907/2006] and CLP [(EC) 1272/2008]

In the EU chemicals framework, the REACH Regulation ((EC) 1907/2006) governs the *Registration, Evaluation, Authorisation and Restriction of Chemicals*. (EC 2006). Further, the *Classification, Labelling and Packaging* (CLP) Regulation ((EC) 1272/2008) covers the classification and labelling of substances and mixtures. (EC 2008b) Both Regulations also include definitions and specific provisions on NMs. To address the specific requirements for NMs, REACH Annexes I, III and VI-XII were revised through Regulation ((EU) 2018/1881), which entered into force on 1st January 2020. (EC, 2018b) Harmonised classifications are listed in Annex VI of the CLP Regulation and are summarised in Table 1 for CuO, Cu_2_O and copper in their non-nanoforms. Their respective nanoforms should be considered separately. (EC 2008b).

**Table 1:** Harmonised classifications for CuO, Cu_2_O and copper

	CuO CAS no: 1317-38-0 (ATP17)	Cu_2_O CAS no: 1317-39-1 (ATP17)	Copper [specific surface area > 0.67 mm²/mg] CAS no: 7440-50-8 (ATP22)
Human hazard		Acute Tox. 4, H302 Eye Dam. 1, H318 Acute Tox. 4, H332	
Environmental hazard	Aquatic Acute 1, H400, M = 100 Aquatic Chronic 1, H410, M = 10	Aquatic Acute 1, H400, M = 100 Aquatic Chronic 1, H410, M = 10	Aquatic Acute 1, H400, M = 10 Aquatic Chronic 1, H410, M = 1

ATP: Adaptation to Technical Progress

### Feed additives [(EC) 1831/2003 and (EU) 2018/1039]

Copper compounds are used in animal feed to meet nutritional requirements and are authorised as a feed additive in the form of organic and inorganic compounds under Regulation (EC) 1831/2003. (EC, 2018b) While CuO and Cu_2_O are authorised as a feed additive, elemental copper is not authorised.

In 2016, the EFSA Panel on Additives and Products or Substances used in Animal Feed (FEEDAP) recommended lowering the maximum total copper content in the complete feed for food-producing animals and pets, in order to protect animal health, safeguard consumers, and especially to reduce environmental copper contamination. A key recommendation was to reduce the maximum copper content in complete feed for piglets from 170 mg/kg to 25 mg/kg. (EFSA 2016a) The current maximum permissible levels of copper in complete feed for different species are implemented in Regulation (EU) 2018/1039. (EC, 2018a).

According to Commission Regulation (EC) 429/2008, which establishes detailed requirements for the preparation and presentation of applications, as well as the assessment and authorisation of feed additives, applicants must provide information on particle size distribution, particle shape, density, bulk density and dusting potential of solid preparations. The Regulation does not contain explicit provisions or definitions regarding NMs or NPs. Consequently, measurement of particle size distribution does not necessarily encompass particles in the nanoscale. The Regulation specifies only that products containing more than 1% of particles with a diameter < 50 µm, on a weight basis, must undergo safety testing in an acute inhalation toxicity study. (EC 2008a) (N.B.: The Regulation is currently under revision at the European level). Notably, to the authors’ knowledge, no copper compounds intentionally produced in nanoforms are currently authorised as feed additives. Yet, the unintentional presence of CuO or Cu_2_O NPs cannot be excluded, due to the absence of nano-specific requirements in the regulation.

Nonetheless, existing guidance documents, while not legally binding, do provide relevant recommendations for the authorisation of feed additives with regard to NMs. Thus, according to the EFSA Guidance on the identity, characterisation and conditions of use of feed additives, it should be clearly indicated if a feed additive is considered a NM under EU legislation. (EFSA 2018) Accordingly, particle size characterisation should be performed when the nature of the additive suggests the potential presence of NPs. In 2021, the EFSA Scientific Committee issued the “Guidance on Particle-TR” (“Guidance on technical requirements for regulated food and feed product applications to establish the presence of small particles including nanoparticles”) (EFSA 2021), which outlines mandatory information requirements for regulated food products for which the EU NM definition under Regulation (EU) 2015/2283 applies directly or indirectly. This includes, among others, feed additives, food supplements, and food additives, irrespective of whether these particles were used intentionally or were unintentionally present. To promote consistency across EFSA assessments on NPs, the Guidance on Particle-TR is also relevant for PPP and FCM. The Guidance on Particle-TR provides a decision framework to determine if the material consists of or contains a certain fraction of small particles, and consequently, whether a NM-specific risk assessment, as described in the “EFSA Guidance on risk assessment of nanomaterials” (EFSA Guidance on Nano-RA) is required, or whether a conventional risk assessment is adequate. (EFSA 2021) The decision process considers parameters including solubility, dissolution rate, particle size distribution, and available safety data. Even though these Guidances are not legally binding, they are regularly used by the EFSA FEEDAP Panel in their assessments. A positive assessment, confirming that the use of a feed additive does not pose safety risks to humans, animals, or the environment, is considered a prerequisite for later authorisation; thus, both Guidances play a relevant role in the assessment process.

### Plant protection products [(EC) 1107/2009]

Authorisation of PPPs in the EU follows a two-step process. First, active substances are evaluated at the EU level. Subsequently, formulated products containing these substances along with their co-formulants, undergo authorisation on national level to confirm their efficacy and ensure protection of human and animal health as well as the environment (EFSA 2021) Copper-based compounds, containing Cu_2_O as active substance, are authorised in the EU and are widely used in PPPs for their antifungal properties, both in organic and conventional agriculture. Foliar spraying remains the most common application method, although soil and seed treatments are also used. In accordance with Regulation (EC) 1107/2009 and based on EFSA’s conclusion from the peer review of the pesticide risk assessment of copper compounds as active substances, i.e., copper hydroxide (CAS No 20427-59-2), copper oxychloride (CAS No 1332-65-6), Cu_2_O, Bordeaux mixture (CAS No 8011-3-0) and tribasic copper sulphate (CAS No 12527-76-3), these substances are currently approved as active substances in PPP. (EC 2009b; EFSA 2018) In contrast, neither for CuO nor for elemental copper applications for approval as active substances in PPPs were made. To the authors’ knowledge, no intentionally produced nanoform of any copper compound is currently approved or placed on the market as an active substance in PPP. However, it should be noted that, since the Regulation (EC) 1107/2009 (EC 2009b) does not include specific provisions or definitions addressing nanoforms of active substances, nanoforms of Cu_2_O may not be explicitly identified or designated as such. Similar to the situation for feed additives, the EFSA Guidances on Particle-TR and Nano-RA provide scientific recommendations for the assessment of NMs in PPPs. Although Guidances are not legally binding, they are routinely used and play a relevant role in the assessment process. (EFSA 2021) According to the EFSA Guidance on Nano-RA, a comprehensive risk assessment addressing nanospecific properties is required, when (i) the material is an active substance in PPP that consists of or contains nanoforms, as defined under Regulations (EU) 2018/1881 and (EU) 2020/878, or (ii) a PPP contains co-formulants present in a nanoform. (EFSA 2021, EC, 2018b, 2020).

### Biocides [(EU) 528/2012]

Biocides are authorised in a two-step process, similar to the one for PPPs. First, the active substance must be approved for use in a specific product-type (PT). Then, the biocidal product for this PT must be authorised. The Biocidal Products Regulation (EU) 528/2012 establishes harmonised rules “concerning the making available on the market and use of biocidal products”. (EU 2012) Under this Regulation, Cu_2_O is approved as a biocidal active substance for PT 21 (antifouling products) and CuO is approved as biocidal active substance in PT 8 (wood preservatives). Both approvals are legally limited to a period of ten years and are subjected to renewal by 30th June 2028 (Cu_2_O) and 31st July 2029 (CuO), respectively. In addition, granulated copper is approved as an active substance in PT 8 until 31st December 2026. For copper as NM, initial applications for approval are currently under evaluation for PT 2 (disinfectants and algaecides not intended for direct application to humans or animals), PT 5 (drinking water) and PT 11 (preservatives for liquid-cooling and processing systems). The Biocidal Products Regulation includes specific provisions addressing NMs. These provisions apply to active substances and biocidal products that meet the criteria of NMs, as defined in the Regulation, which is aligned with the EC Recommendation. (EC 2022) For intentionally produced nanoforms of active substances a separate dossier fulfilling all data requirements must usually be submitted for evaluation. (EU 2012) A specific risk assessment is required when nanoforms of either active or non-active substances are used in a biocidal product. However, unintentionally produced nanoforms of active substances of CuO or Cu_2_O may remain unidentified, as the core data set for active substances described in Annex II of the Regulation does not require a general particle size distribution analysis. Only for the final biocidal products a size distribution analysis is part of the core data set, as described in Annex III. (EU 2012) As a result, a biocidal product may be classified as conventional if the formulation as a whole no longer meets the criteria of NMs, despite containing an active substance that is unintentionally a NM.

To date, only a limited number of nanoscale biocidal active substances have been approved in general (e.g., pyrogenic, synthetic amorphous, nano, surface treated silicon dioxide (SiO_2_, CAS No 68909-20-6) for PT 18 (insecticides, acaricides and products to control other arthropods)). At the time of writing of this article, no copper-containing substances intentionally produced in nanoform are approved for use in biocides.

### Fertilisers [(EU) 2019/1009]

Regulation (EU) 2019/1009 states the rules governing the making available on the market of EU fertilising products. (EU 2019) For the various categories of fertilisers (e.g., organic fertilisers, inorganic micronutrient fertilisers, organic soil improvers), maximum permissible copper contents are specified (see Regulation (EU) 2019/1009 for detailed values). However, copper itself may be placed on the market as a fertilising agent. The Regulation does not define the typology of micronutrients in fertilisers; i.e., salts, oxides, chelates among other forms are allowed. Consequently, CuO or Cu_2_O may also be used as fertilisers. These could be potentially be present in nanoforms, as the Regulation neither addresses nanoforms nor requires a detailed size distribution analysis. (EU 2019).

### Food supplements [(EC) 1170/2009]

Minerals and vitamins allowed for use in the manufacture of food supplements are listed in Annex I of Directive 2002/46/EC. (EC 2002) The allowed chemical forms are further specified in Annex II of the same Directive including copper and CuO. (EC 2002) In 2009, the EFSA Scientific Panel on Food Additives and Nutrient Sources Added to Food (ANS) published a Scientific Opinion on CuO as a source of copper intended for nutritional use in food supplements. (EFSA 2009) The Panel concluded that CuO as a source of copper at the proposed use level is considered safe, provided that the Tolerable Upper Intake Level (UL) is not exceeded. Also in 2009, Directive 2002/46/EC was amended by Regulation (EC) 1170/2009, stating that copper in the form of CuO may be used for the manufacture of food supplements. (EC 2002, 2009a) Additional allowed forms of copper include copper L-aspartate, copper bisglycinate and copper lysine complex (Annex II of Regulation (EC) 1170/2009). (EC 2009a) The Regulation does not address nanoforms and also does not require a detailed particle size distribution analysis. Consequently, the presence of CuO NPs in food supplements cannot be excluded. However, the EFSA Guidance on Particle-TR is applicable to food supplements and enables the determination of whether a material consists of or contains a certain fraction of small particles. Where this is the case, a nano-specific risk assessment must be performed in accordance with the EFSA Guidance on Nano-RA. (EFSA 2021).

### Food additives [(EC) 1333/2008]

According to Annex II of the Food Additives Ordinance (Regulation (EC) 1333/2008), the use of copper complexes of chlorophylls and chlorophyllins as a food additive (E 141) is permitted in several food categories under the *quantum satis* principle, meaning without maximum level restrictions. In contrast, CuO, Cu_2_O and elemental copper, whether in non-nanoforms or nanoforms, are currently not permitted as food additives. (EC 2008c) The EFSA Guidance on Particle-TR also applies for food additives and provides a framework for determining whether a material consists of or contains a certain fraction of small particles. If this is the case, a nano-specific risk assessment in accordance with the EFSA Guidance on Nano-RA is required. (EFSA 2021).

### Drinking water [(EU) 2020/2184]

The quality of water intended for human consumption is regulated by Directive (EU) 2020/2184. (EU 2020) The minimum requirements for parametric values used to assess drinking water quality are stated in Annex I. In Part B “Chemical parameters”, the maximum concentration of copper is restricted to 2 mg/L. The Directive does not specify the typology of copper or other metals in drinking water and refers only to total copper in general. Additionally, it does not address nanoforms or requires a detailed particle size distribution analysis. Consequently, the presence of CuO or Cu_2_O in nanoform cannot be excluded.

### Food contact materials [(EC) 1935/2004 and (EU) 10/2011]

According to Article 3 of the EU Framework Regulation on food contact materials (FCM) (Regulation (EC) 1935/2004), materials and articles intended to come into contact with food must, under normal or foreseeable conditions of use, not release their constituents into food in quantities that could endanger human health. (EC 2004) In principle, no general authorisation requirement exists for FCM or substances contained therein. However, in accordance to Articles 5 and 6 of the Regulation, specific measures may be adopted at the EU or national level for certain groups of materials and articles. These measures may include the establishment of so-called positive lists, i.e., lists of substances authorised for use in the manufacture of materials and articles intended to come into contact with food – as well as, where relevant, other conditions of use. An example of such an individual measure is the Regulation (EU) 10/2011 on plastic materials and articles intended to come into contact with food. (EC 2011b) According to Annex I of this Regulation, several copper compounds (i.e., copper iodide, copper bromide and copper hydroxide phosphate) are authorised for intentional use in the manufacture of plastic layers in plastic materials and articles. The substance migration limit (SML) for copper migrating from plastic materials and articles is set at 5 mg/kg of food or food simulant (Annex I of Regulation (EU) 10/2011). The Regulation mentions NPs, yet does not provide a definition, and explicitly states that authorisations based on conventional particle sizes do not apply to engineered NPs. For example, titanium nitride NPs are specifically authorised as an additive or polymer production aid. In contrast, copper compounds in nanoform, or CuO or Cu_2_O in non-nanoform are currently not authorised. At the same time, the Regulation does not require a detailed particle size distribution analysis; therefore the presence of unintentionally produced NPs, e.g. from other metal oxides, cannot be excluded. (EC 2011b) However, for FCM also the EFSA Guidances on Particle-TA and Nano-RA are applicable and complement the existing FCM-specific Regulations. According to the EFSA Guidance on Nano-RA a comprehensive risk assessment addressing nanospecific properties is required, when (i) material is a substance in the manufacture of FCMs that is in nanoform as defined under Regulation (EU) 10/2011 or (ii) the material is deliberately engineered to a particle size that exhibits functional physical and chemical properties significantly different from those of its larger-scale counterpart, in accordance to Regulation (EC) 450/2009 (EFSA 2021; EC 2011b, EG, 2009a).

### Cosmetics ((EC) 1223/2009)

The legal framework for copper, CuO, and Cu_2_O in the cosmetic sector is also of interest, as this is the only sector in which a copper NM – in this case elemental copper – is specifically prohibited due to toxicological concerns. Cosmetics are also a relevant sector for oral exposure, as for example toothpaste and lipsticks fall under these regulations. In addition, the EU Cosmetics Regulation ((EC) No 1223/2009) provides more comprehensive provisions on NMs, including a distinct definition, than Regulations within the food and feed sector. (EC 2009c) In the Regulation, CuO and Cu_2_O are not specifically regulated, however, non-nano elemental copper is listed as an allowed colorant in Annex IV (reference number 132), with the restriction “only as colorant”. (EC 2009c) According to Article 16 (10a) of the EU Cosmetics Regulation, the EC is responsible for publishing and maintaining a catalogue listing all NMs contained in cosmetic products available on the market. The Regulation defines a NM as an insoluble or biopersistant and intentionally manufactured material with one or more external dimensions, or an internal structure, on the scale from 1 to 100 nm. Both, colloidal copper (for dermal and oral applications) and copper (for dermal applications) are listed in the current “EC catalogue for nanomaterials” published in 2019. (EC 2019) In 2021, the Scientific Committee on Consumer Safety (SCCS) issued two publications concering colloidal copper (nano) and copper (nano). First, general recommendations on the risk assessment of NMs in cosmetic products were published (SCCS/1618/20) (Bernauer et al. 2021) As part of this, the NMs listed in the EC catalogue of 2019 were prioritised according to their potential health risks. To this end, the scoring system suggested by Brand et al*.* was applied. (Brand et al. 2020) This system was originally developed for ranking potential health risks associated with food substances, considering four descriptors (i.e., physico-chemical properties, hazard, kinetics, and exposure). Within this ranking, colloidal copper occupied the highest position (1 out of 29 listed NMs), prompting the SCCS to highlight the need for further safety evaluation of copper NMs. Second, the SCCS published a Scientific Opinion on copper (nano) and colloidal copper (nano) (SCCS/1621/2020). (Bernauer et al. 2020) The Committee concluded that the available data provided were insufficient to perform an adequate and comprehensive risk assessment. Nevertheless, the submitted data indicated the potential for systemic uptake of copper NPs (and/or ionic copper), which could lead to accumulation in certain organs, particularly in liver and spleen. In addition, the SCCS raised concerns regarding potential mutagenic/genotoxic and immunotoxic/nephrotoxic effects associated with copper NMs. Since 2024, copper (nano) and colloidal copper (nano) have been banned from use in any cosmetic products placed on the market or used in the EU—as amended by Regulation (EU) 2024/858 and listed in Annex II of the EU Cosmetics Regulation. (EC 2009c, 2024) According to Article 13 of the EU Cosmetics Regulation, the presence of substances in nanoform shall be submitted to the Commission prior to placing a cosmetic product on the market. (EC 2009c) Additionally, parameters and methods for the identification and characterisation of nanomaterials)—including particle size and size distribution—intended for use in cosmetic products are specified in a SCCS guidance document (SCCS/1655/23. (Bernauer et al. 2023) Among the legislative frameworks discussed in this review, the EU Cosmetics Regulation provides the most specific and comprehensive provisions concerning nanomaterials. However, despite the ban on copper and colloidal copper NMs, the Regulation does not explicitly require manufacturers to conduct a detailed particle size distribution assessment to ensure the absence of unintentionally produced NMs. In fact, the definition of NMs under this regulation explicitly only applies to intentionally manufactured NMs.

### Conclusions of the section

In the preceding sections we have thoroughly reviewed current EU legislations to assess whether CuO and Cu_2_O NPs are adequately addressed within regulatory frameworks to prevent unintentional oral exposure through the food and feed chain. Overall, nanoforms of copper and copper oxides are not uniformly or consistently addressed across EU legislation. While some EU Regulations already include specific data requirements for applicants for intentionally produced NMs, others do not address nanoforms at all.

A recurring regulatory gap identified across most reviewed frameworks concerns the distinction between intentionally produced and unintentionally produced NMs. In particular, the absence of mandatory and harmonised requirements for particle size distribution analysis means that particulate materials may not be systematically assessed for the presence of nanoscale fractions. As a result, CuO or Cu_2_O NPs may remain unidentified where nanoforms are not intentionally used.

Against this background, the EFSA Guidance on Particle-TR can be applied across several of the reviewed regulatory areas, including feed additives, PPP, food supplements, food additives, and FCM. This Guidance provides definitions for NPs and NMs and outlines a decision-making framework to determine, whether a material consists of, or contains, a specific fraction of small particles. Based on this assessment, it further indicates whether a risk assessment following the EFSA Guidance on risk assessment of nanomaterials (EFSA Guidance on Nano-RA) is required, or whether a risk assessment as a conventional material is sufficient. (EFSA 2021, EFSA 2023) To the best of our knowledge, this Guidance is frequently referenced during the application process by both manufacturers and regulatory authorities. Integration of the considerations outlined in these Guidance documents into legally binding regulatory frameworks is recommended in order to enhance consistency in regulatory decision-making, reduce ambiguity regarding data requirements, and improve the reliability and transparency of NP safety evaluations across sectors.

Taken together, the reviewed regulatory frameworks indicate that the unintentional presence of Cu, CuO, and/or Cu_2_O NPs from different regulated sectors cannot be excluded, whereas the intentional use of copper-containing NMs is currently very unlikely. Consequently, the entry of such NPs into the food and feed chain may occur unintentionally. In the following section, we therefore review the current knowledge on human exposure to Cu, CuO, and Cu_2_O in both nanoform and non-nanoforms via the food and feed chain.

## Exposure to copper, CuO and Cu_2_O and their nanoforms

As outlined in the previous section, it is possible that nanoforms of copper, CuO or Cu_2_O enter the food and feed chain unintentionally. To understand the impact of this regulatory gap, it is therefore essential to examine human exposure to copper and its nanoforms. In 2023, the EFSA Scientific Committee performed a copper exposure assessment from all sources when re-evaluating the health-based guidance values (HBGVs). With the re-evaluation using the harmonised approach for establishing HBGVs for regulated substances that are also nutrients from 2021, the initial acceptable daily intake (ADI) of 0.15 mg/kg bodyweight (bw) was lowered to 0.07 mg/kg bw. The current ADI is based on the conclusion that copper retention, referring to gradual accumulation in the body due to insufficient excretion, is not expected at daily intakes below 5 mg. It was concluded that dietary intake is the primary source of copper for the general population, while dermal or inhalation exposure is only reported in occupational settings. (EFSA 2023).

The total dietary copper consumption of adults is approx. 1.04 to 1.62 mg per day, with the main contributing food categories being grains, fruits, meat, vegetables, coffee and tea as well as milk. The EFSA further concludes that natural copper background levels are the most significant source of copper and contribution of regulated use in different sectors is negligible (EFSA 2023) However, while copper as a ubiquitously present metal enters the food chain in non-nanoform, regulated use of CuO and/or Cu_2_O in feed additives, PPPs, biocides, fertilisers, food supplements or drinking water may lead to the unintentional entry of nanoforms into the food chain. This was not considered in the EFSA assessment and to our knowledge, there are also no studies available that assess the amounts of CuO or Cu_2_O NPs in relevant foods on the market. Thus, it is not possible to estimate the contribution of those NPs to the total copper exposure nor to identify relevant sources.

A potential entry of CuO or Cu_2_O NPs into the food chain is the use in feed additives, which are not specified as nanoforms due to regulatory gaps. To ensure adequate copper intake, particularly in rapidly growing farm animals or in species with continuous copper losses through the production of eggs and milk, copper-containing feed additives are commonly used. These additives may consist of inorganic, such as CuO or Cu_2_O, or organic copper compounds, such as chelates of amino acids. Copper sulphate (CuSO_4_) is the most widely applied form, due to its favourable bioavailability. Common inorganic copper preparations are frequently bound to amino acids. (Espinosa and Stein 2021) Species-specific copper requirements are established by scientific bodies in Europe and the United States, with comprehensive summaries on individual requirements available, e.g., in EFSA (2016a, b). (EFSA 2016a) In pig production, high copper supplementation is often used to exploit its pharmacological properties. In this case copper doses in feed frequently exceed the nutritional requirements of animals, particularly during early postnatal growth phase, with the aim of preventing piglet mortality and improving growth performance. Copper is partially accumulated in animal tissues, such as liver, kidney, and muscles. Exceeding the permissible maximum copper content in foods of animal origin, as defined in Regulation (EC) 396/2005), most frequently affects liver, especially beef liver. (BVL 2022, 2021) As a result, liver from beef or sheep and their derived products represent the major dietary source of copper for humans. (EC 2005) In line with that, individuals who consume high amounts of mammalian liver were identified as the only group that exceeds the ADI for copper within the BfR MEAL study from 2023. (Kolbaum et al. 2023) In order to identify whether feed additives are a potential source of CuO or Cu_2_O NPs it could therefore be a first step to assess their presence in liver and liver derived products.

The assessment of transfer of CuO or Cu_2_O NPs from feed additives into animal derived products is especially important given the ongoing discussions on the intentional use of nanoscale copper formulations. Several reviews have summarised the research on the potential use of nanoscale copper additives in animal nutrition, highlighting the opportunity to reduce the total copper content required in feed due to higher bioavailability, but there are still issues about the pathways and the toxicity. (Casale et al. 2025; Scott et al. 2018; Sabry et al. 2021) The reduction of total copper content in feed is one effort to prevent further copper accumulation in the soil, as excess dietary copper that exceeds animals’ nutritional requirements is largely excreted via the faeces (Schulz et al. 2025), entering the soil through manure.

Elevated copper levels in the soil are unfavourable for several reasons, including the suspected contribution to the development of antimicrobial resistance of soil microorganisms as well as the increase of copper in crop. (EFSA 2016a, EFSA 2023) Whether the applied manure contains CuO or Cu_2_O NPs and whether they remain in the soil is not investigated. However, the use of CuO and/or Cu_2_O containing PPPs and fertilisers does not only contribute to the accumulation of total copper in the soil, but also holds the potential to release nanoforms into the environment unintentionally. To prevent copper accumulation in soil—especially in permanent crops like vineyards, the use of copper-based PPPs is strictly regulated (i.e., limited to 4 kg Cu/ha/year in organic farming). (EFSA 2023) The EFSA concludes that elevated copper levels in soil due to the use of manure, PPPs or fertilisers on copper levels in plant derived products are of minor importance. (EFSA 2023) However, these considerations are based on total copper levels and do not take the potential presence of CuO or Cu_2_O NPs into account. To our knowledge there are currently no data available to estimate the amount of unintentionally produced CuO and/or Cu_2_O NPs in PPPs or fertilisers. This is an important data gap, as experimental data demonstrates that CuO and other metal oxide NPs can be taken up from soil. In experiments using lettuce, CuO NPs applied to a nutrient solution entered the plants and distributed into roots and shoots. (Li Jinxing et al. 2020) Further, the application of PPPs with copper in nanoform and non-nanoforms can directly lead to accumulation of copper in crop. (Xiong and Li 2022) For instance, it was demonstrated that CuO NPs can penetrate the plant cell wall when applied to leaves of *Zea mays*. (Adhikari et al. 2016) This is especially relevant, given that nano copper PPPs are already used outside the EU, as reviewed by Casale et al. 2025. 

Environmental accumulation of copper and potentially copper nanoforms may not only lead to transfer into plant derived foods but also into waterbodies and reach drinking water sources. Drinking water can also contribute to copper exposure. Depending on local conditions, concentrations in water are typically below the legally binding maximum limit for total copper of 0.2 mg/L in the EU [(EU) 2020/2184], but may occasionally reach higher levels in rare cases as high as 6.4 mg/L (Zietz et al. 2003; EU 2020). Importantly, these data refer to total copper and do not distinguish between ionic, particulate, or nanoscale forms; consequently, the presence of CuO or Cu_2_O NPs cannot be excluded, although no data are currently available to assess their occurrence in drinking water. In this context, it is noteworthy that initial applications for approval of elemental copper in nanoform are currently under evaluation for biocidal product types including drinking water.

In contrast to dietary and environmental exposure pathways, the use of copper-containing PPPs and biocidal products gives rise to exposure scenarios that are primarily use-related and affect specific population groups. PPPs are mostly applied by foliar spraying, thus exposure mainly occurs via dermal and inhalation routes during product handling, application, and re-entry activities. The use of personal protective equipment (PPE) effectively reduces operator and worker exposure to levels below the acceptable operator exposure level (AOEL). Similarly, exposure levels of residents and bystanders generally remain within acceptable safety margins when appropriate risk mitigation measures (RMMs), such as buffer zones, are applied. (EFSA 2013, 2018) However, in some high-use scenarios like grape cultivation, worker exposure can exceed the AOEL even when PPE is used. (EFSA 2018) The AOEL and the PPE do not consider the presence of Cu_2_O NPs, which may unintentionally be present in PPPs. Thus, dermal and inhalation exposure of Cu_2_O NPs may be of relevance in occupational settings where PPPs are used. Also, the use of biocidal products may result in dermal and inhalation exposure to copper compounds and potentially to unintentionally present CuO or Cu_2_O NPs. Cu_2_O is approved as active substance in antifouling paints (PT21) and CuO in wood preservatives used in professional settings (PT8) (see above). In both cases, exposure primarily occurs during professional handling, application and post-application cleaning. Oral exposure may occur in non-professional settings, for instance when toddlers touch wet or dry paint followed by hand-to-mouth transfer, or when infants are chronically exposed through mouthing of treated timber surfaces and acutely exposed by chewing on preserved timber off-cuts. Regarding human health, the use of Cu_2_O-antifouling products is considered safe for both professional and non-professional applications, provided that PPE is used. (EFSA 2016b; ECHA 2016) Similarly, the proposed uses of CuO in wood preservatives are considered safe for industrial and professional users, as well as for indirectly exposed persons of the general public. (ECHA 2011) However, these assessments are based on total copper and do not consider the potential presence or behaviour of unintentionally produced nanoforms. Consequently, while current regulatory evaluations indicate no safety concern for conventional copper compounds, exposure to CuO or Cu_2_O NPs cannot be excluded. To date, no data are available that quantify the presence of such nanoforms in biocidal products or assess their contribution to human exposure, representing a relevant data gap.

Overall, exposure to copper is considered to be within the acceptable limits for the general population, with dietary intake representing the primary exposure pathway. Regulated uses of copper compounds in animal nutrition, PPPs, biocides, fertilisers, food supplements and drinking water are generally considered safe for consumers, workers and bystanders when evaluated on the basis of total copper. (EFSA 2023, EFSA 2016b; ECHA 2011, 2016) However, across all assessed exposure pathways, including foods of animal and plant origin, drinking water, and use-related exposure scenarios, available data do not differentiate between ionic, particulate or nanoscale forms of copper. At present, the main concern of regulatory bodies regarding copper containing products used in animal nutrition and PPPs relates to their environmental impact, and initial measures to reduce copper input in soil have been implemented. (Tamm et al. 2022) The potential unintentional presence of CuO or Cu_2_O NPs has only recently gained attention. Thus, none or only limited experimental data are available on the occurrence and transfer of CuO or Cu_2_O NPs from feed additives into animal-derived foods, from soils or PPPs into crops, or from PPPs, fertilisers or biocidal products into environmental compartments relevant for human exposure. This represents a substantial knowledge gap that limits the interpretation of exposure assessments in the context of nano-specific risks. NPs may exhibit different behaviours and biodistribution patterns in the body compared to ionic copper, potentially leading to toxicological outcomes. Furthermore, the effects might be similar in nature, yet differ in magnitude, site of action, or target organs.

## Overview of copper and copper oxide homeostasis and toxicity

This section provides an overview of biological uptake pathways of copper, homeostatic regulation, and toxicological mechanisms. This context is essential for assessing copper-specific NP toxicity relative to endogenous copper physiology and established mechanisms of copper-induced toxicity. Furthermore, by outlining the essential principles governing general copper behaviour in biological systems, it establishes a basis for understanding deviations in kinetics and effects observed for copper in NP form and supports the subsequent sections on the systematic and bibliometric analysis of copper oxide NP research and toxicity mechanisms.

### General copper homeostasis and physiology

**Fig. 2:**
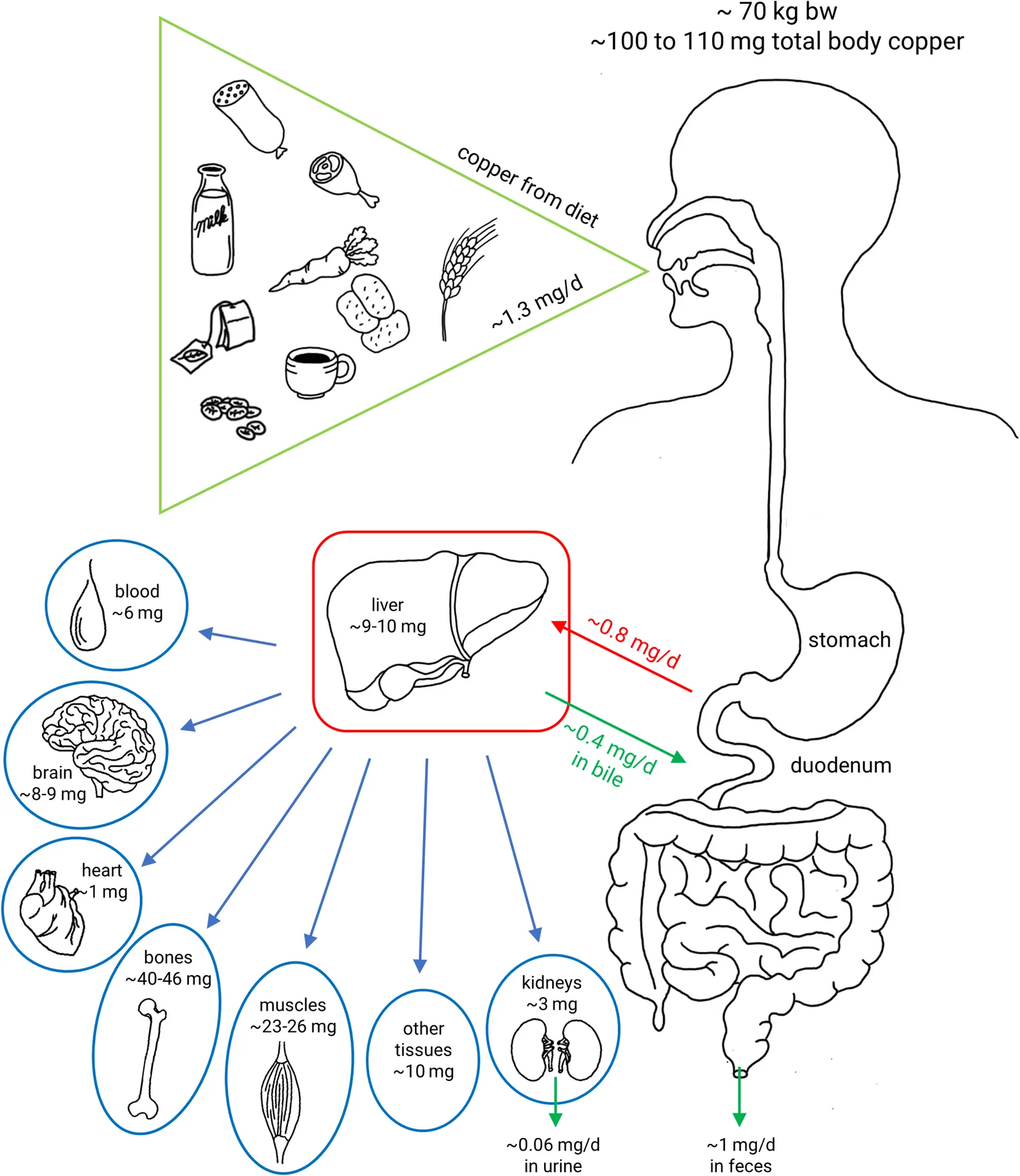
Schematic overview of copper uptake from the diet, its distribution within the body, enterohepatic circulation, and excretion. bw: body weight. (Figure created based on information from Espinosa et al. 2021, van Campen et al. 1965, Forcarelli et al. 2022, Wijmenga et al. 2004 and Collins et al. 2017)

Copper is an essential micronutrient that plays important physiological roles as a cofactor in cuproenzymes, which exploit its redox activity to mediate single electron transfers (reviewed by Collins 2021 and Schreiber et al. 2013). Two key cuproenzymes include superoxide dismutase (SOD), one of the primary antioxidant enzyme systems (Tainer et al. 1983), and the cytochrome c oxidase (COX) enzyme, a component of the mitochondrial electron transport chain (ETC) for ATP generation (Richter and Ludwig 2003). Orally ingested copper is absorbed at various sites of the gastrointestinal tract (GIT), with the majority of absorption occurring in the duodenum and, to a lesser extent, in the stomach (Fig. 2). (van Campen and Mitchell 1965) Following absorption, copper reaches the liver, which is the central organ responsible for copper redistribution and excretion. Approximately 98% of copper is excreted via the bile. (Wijmenga and Klomp 2004) On average, a 70-kg person has an estimated total body copper content of 100 to 110 mg, of which ~ 46 mg are present in skeleton/bone marrow, 26 mg in muscles, 10 mg in the liver, 9 mg in the brain and 6 mg in the blood. When normalised to organ weight, the kidney contains the highest copper levels (~ 12 µg/g tissue), followed by the liver (~ 6 µg/g tissue), brain (~ 5 µg/g tissue) and heart (~ 5 µg/g tissue). (Focarelli et al. 2022).

**Fig. 3:**
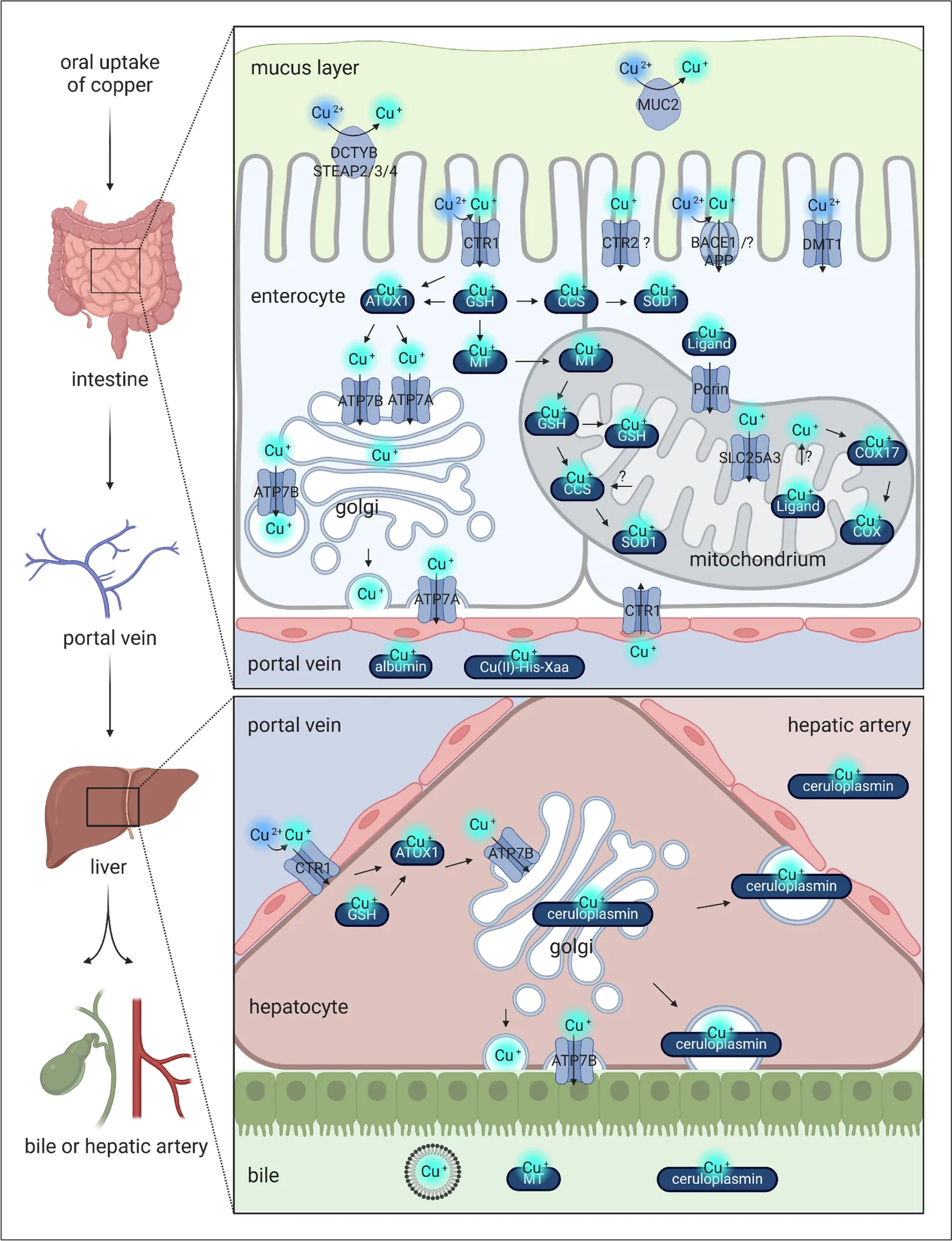
Copper homeostasis in humans. Dietary copper ions are absorbed in the intestine and enter enterocytes via specific transporters, from where they are transported to mitochondria for the metalation of cuproenzymes. Subsequently, copper ions are transferred through the Golgi system into portal circulation. After reaching the liver, hepatocytes either excrete copper ions into the bile or release them into systemic circulation. APP: amyloid precursor protein; ATOX1: antioxidant 1 copper chaperone; ATP7A/B: ATPase copper transporting alpha/beta; CCS: copper chaperone for superoxide dismutase; COX/17: cytochrome c oxidase/ 17; CTR1/2: copper transporter 1/2; BACE: β-Site APP-Cleaving Enzyme 1; DCTYB: cytochrome b reductase 1; DMT1: divalent metal transporter 1; GSH: glutathione; MT: metallothionein; MUC2: mucin 2; SOD1: superoxide dismutase 1; STEAP2/3/4: six-transmembrane epithelial antigen of the prostate 2/3/4. Figure was created in BioRender. Scholz, S. (2026) https://BioRender.com/o2b75pw

Copper homeostasis in humans is tightly regulated and generally well understood (Fig. 3). Regulation of dietary copper uptake begins in the intestinal lumen. Recent findings by Reznik et al. revealed that mucin 2 (MUC2), the principal protein component of intestinal mucus, has multiple roles in modulating copper absorption. (Reznik et al. 2022) By binding copper ions in both oxidation states at distinct binding sites, MUC2 mitigates the formation of reactive oxygen species (ROS) and protects enterocytes from copper-induced toxicity. Moreover, MUC2 enhances copper bioavailability by promoting the reduction of Cu^2+^ to Cu^+^, the latter one being the oxidation state preferentially transported by most known copper transporters. (Lee et al. 2002) Additionally, under conditions of copper depletion in enterocytes, MUC2 can release bound copper ions supporting to maintain adequate intracellular levels. (Reznik et al. 2022) The reduction of copper ions is further mediated by trans-plasma-membrane oxidoreductases, including duodenal cytochrome b reductase 1 (DCTYB) and members of the six-transmembrane epithelial antigen of the prostate (STEAP) family. DCTYB catalyses the reduction of Cu^2+^ and Fe^3+^ ions, as well as ascorbyl radicals, in the duodenum. (Lane et al. 2015) STEAP2, STEAP3, STEAP4 also participate in Cu^2+^ and Fe^3+^ reduction, while the physiological role of STEAP1 remains under debate (Ohgami et al. 2006; Rocha et al. 2021; Xu et al. 2022).

The primary apical copper ion transporter in enterocytes is copper transporter 1 (CTR1), also known as solute carrier family 31 member 1 (SLC31A1). (Lee et al. 2002) CTR1 directly transports Cu^+^, however, recent evidence indicates that it also contributes to the reduction of Cu^2+^ to Cu^+^ by binding Cu^2+^ ions at its extracellular N-terminal domain and stabilising the reduced Cu^+^ species in the presence of reductants such as ascorbate. (Kar et al. 2022) The abundance of CTR1 at the plasma membrane is regulated by intracellular copper levels, as Cu^+^ interacts with intracellular domains of CTR1 to modulate its trafficking. (Kar et al. 2022) In addition to its apical localisation, CTR1 is also expressed on the basolateral membrane of intestinal cells, where it mediates copper uptake from the blood stream under conditions of dietary copper deficiency. (Zimnicka et al. 2007) Divalent metal transporter 1 (DMT1), known for transporting Fe^2+^ and Mn^2+^ (Zimnicka et al. 2011), was initially proposed to facilitate Cu^+^ transport in intestinal cells. (Arredondo et al. 2003) Recent evidence, however, suggests that DMT1 may instead transport Cu^2+^. (Pezacki et al. 2022) Nonetheless, the role of DMT1 in copper uptake remains controversial, as DMT1-deficient mice exhibit normal copper absorption, (Shawki et al. 2015), and elevated iron or manganese levels did not diminish copper uptake in Caco-2 cells. (Zimnicka et al. 2011) Thus, while DMT1 may have the ability to transport Cu^2+^, it is unlikely to serve as the primary copper transporter. β-site APP-cleaving enzyme 1 (BACE1) and amyloid precursor protein (APP) are known to facilitate Cu^2+^ reduction and, upon trimerisation, mediate Cu^+^ transport in the brain. (Angeletti et al. 2005, Bethge, 2010, Multhaup et al. 1996) Structural analyses further support their roles as copper transporters. (Munter et al. 2013; Bittner et al. 2018) Although the involvement of BACE1 and APP in intestinal copper absorption has not yet been investigated, both proteins are expressed in Caco-2 cells and show moderate to low expression levels, respectively, in human intestinal tissue. (Lee et al. 2016, proteinatlas.org, 2025a, b, Puig et al. 2015) However, since protein expression alone does not confirm a functional role, this requires further investigation.

Cytosolic copper ion trafficking is tightly regulated to prevent copper-induced redox reactions. Copper ions are usually not present in an unbound state, but are transferred sequentially between binding partners along an affinity gradient (Banci et al. 2010) The main cytosolic copper-binding molecules include glutathione (GSH), metallothionein (MT), and the two copper chaperones: copper chaperone for superoxide dismutase (CCS) and antioxidant 1 copper chaperone (ATOX1). GSH plays a crucial role in maintaining copper homeostasis, serving as both the primary and strongest (Banci et al. 2010) copper-binding partner and the intracellular rate-limiting factor for copper uptake. (Freedman et al. 1989, Maryon et al. 2013) When the GSH pool becomes saturated, excess copper ions bind to MT for secure intracellular storage. (Freedman et al. 1989; Tapia et al. 2004) Copper ions are also transferred from GSH to CSS, which subsequently passes them on to superoxide dismutase 1 (SOD1), the only cytosolic cuproenzyme. (Palumaa 2013) ATOX1 acquires copper ions from either GSH or CTR1 (Kahra et al. 2016) and transfers them to the copper-transporting ATPases ATP7A and ATP7B, located in the Golgi apparatus, where they mediate copper incorporation into secretory pathway proteins. (Hamza et al. 2003).

Copper ions are imported into mitochondria, where the cuproenzyme COX resides. The mitochondrial copper pool within the matrix supports COX metalation, which occurs in the intermembrane space (IMS). (Boulet et al. 2018) This process requires a transport system capable of trafficking copper ions from the cytosol into the IMS, then into the matrix, and back into the IMS. However, until now this system remains poorly understood. Current models propose that copper ions bind to an unidentified, non-proteinaceous anionic ligand in the cytosol and diffuse through mitochondrial porins into the IMS (reviewed by Garza et al. 2023). From the IMS, copper ions can be transported into the matrix via solute carrier family 25 member 3 (SLC25A3). (Boulet et al. 2018) Alternatively, copper ions may enter the IMS bound to MT, subsequently associate with GSH and then be transported into the matrix (reviewed by Lindeque et al. 2010). To date, to our knowledge, no transport mechanisms have been identified for the transport of copper ions from the mitochondrial matrix back into the IMS. After re-entry into the IMS, copper ions are transferred to COX17 by a yet unidentified mechanism. This may occur through a direct interaction between COX17 and an unknown inner membrane transporter, analogous to the CTR1 and ATOX1 interaction (Kahra et al. 2016) or via copper transfer from GSH. (Lindeque et al. 2010; Banci et al. 2010) COX17 subsequently delivers copper ions to COX11 and synthesis of cytochrome c oxidase 1 (SCO1), which then transfer the metal to COX1 (Cu B site) and COX2 (Cu A site), respectively, mediating the maturation of the COX enzyme. (Nývltová et al. 2022) In addition to the COX enzyme, also SOD1 is localised in the mitochondria. Both CCS and SOD1 are imported into the IMS in an unfolded state via the translocator of the outer membrane (TOM) complex, where they fold and participate in copper ion transport, with mature SOD1 remaining within the IMS. (Trist et al. 2021) The source of copper ions for mitochondrial CCS remains uncertain; it may acquire copper ions from GSH or via direct interaction with an unidentified inner mitochondrial membrane transporter.

Copper ion efflux is mediated by the ATPases ATP7A and ATP7B, which have distinct physiological roles across different tissues. After copper ions bind to ATOX1, the metal ion is transported to the Golgi apparatus by ATP7A and ATP7B. (Padilla-Benavides et al. 2013) In the intestine, ATP7A is the predominant isoform, transporting copper ions into the Golgi lumen where cuproproteins are loaded with the metal ion. (Petris et al. 1996; Polishchuk and Lutsenko 2013) When intracellular copper ion concentrations are elevated, ATP7A translocates to vesicles that sequester copper ions and subsequently fuse with the basolateral membrane (Petris et al. 1996; Nyasae et al. 2007); or ATP7A directly integrates into the basolateral membrane to enable continuous copper ion efflux. (Pierson and Lutsenko 2019) Once intracellular copper ion levels normalise, ATP7A relocates back to the Golgi apparatus. (Nyasae et al. 2007) Through this mechanism, absorbed copper ions enter the portal circulation, where they bind to albumin and Cu(II)-His-Xaa ternary complex. (Kirsipuu et al. 2020) The role of ATP7B in the intestine remains less well defined, however, it has been proposed to enable intracellular sequestration under conditions of elevated copper ions by translocating to vesicles that accumulate copper ions without fusing with the plasma membrane. (Pierson and Lutsenko 2019) In the liver, ATP7B mediates copper ion transport into the Golgi apparatus, where it supports cuproproteins metallation, vesicle formation, and the incorporation of ATP7B into the basolateral membrane to enable copper ion effluxeither into the bile for excretion or into blood for systemic distribution. (Roelofsen et al. 2000, Pierson and Lutsenko 2019) For biliary excretion, copper ions are transported into the bile, where they are found in the micellar fraction, complexed with small molecules, and partially bound to MT or ceruloplasmin (reviewed by Linder et al. 2020). For systemic distribution, copper is incorporated into ceruloplasmin within the Golgi apparatus of hepatocytes, where each ceruloplasmin can bind up to seven copper atoms. (Campbell et al. 1981).

In the bloodstream, copper ions are bound to several different plasma transport proteins. The majority of copper ions derived from the liver (~ 75%) is tightly bound in a nonexchangeable equilibrium to ceruloplasmin. The remaining copper ions exists in an exchangeable pool, bound primarily to albumin (~ 25%) or present as Cu(II)-His-Xaa complexes (~ 0.2%), which originate mainly from intestinal absorption. (Kirsipuu et al. 2020) Alpha-2-macroglobulin, also termed transcuprein, has been proposed as an additional potential plasma copper ion carrier (Weiss and Linder 1985), although more recent evidence indicates a minor role in copper ion transport. (Kirsipuu et al. 2020) Ceruloplasmin represents the primary copper ion donor for peripheral tissues (Campbell et al. 1981). Nevertheless, in case of ceruloplasmin deficiency, alternative transport mechanisms maintain systemic copper homeostasis. (Meyer et al. 2001, Miyajima, 2014) Cellular uptake of ceruloplasmin-bound copper ions involves reduction of reduced to Cu^2+^ to Cu^+^, followed by import into cells through CTR1 (Ramos et al. 2016) Albumin-bound copper ions can also be delivered to cells via the interaction with the CTR1 transporter. (Shenberger et al. 2015; Schulte et al. 2023).

### Copper toxicity

#### Molecular mechanisms of copper toxicity

**Fig. 4:**
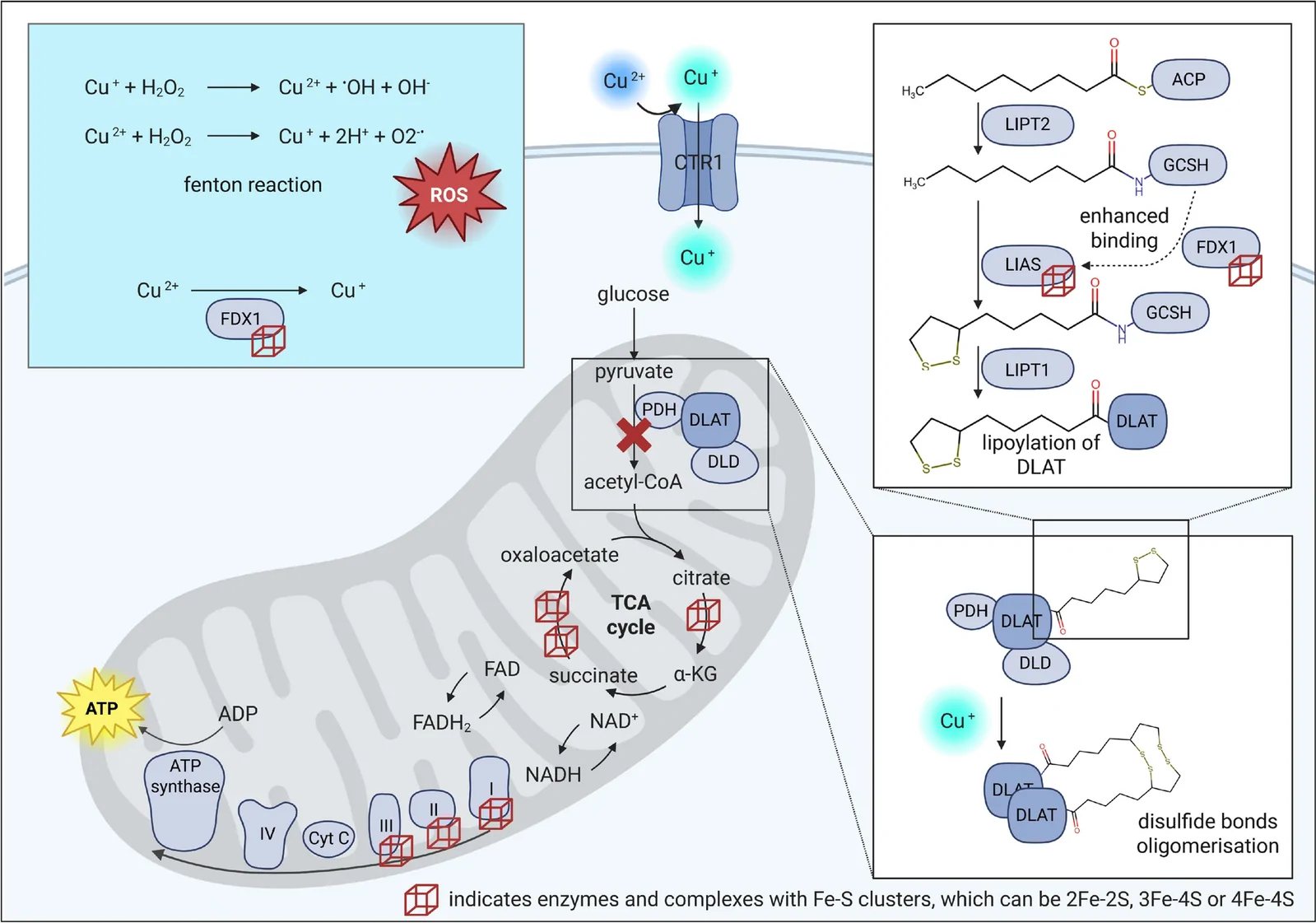
Copper toxicity is mediated by DLAT oligomerisation, interference with Fe-S cluster integrity, and production of ROS. α-KG: alpha-ketoglutarate; ACP: acyl carrier protein; ATP/ADP: adenosine triphosphate/adenosine diphosphate; Cyt C: cytochrome c; I/II/III/IV: complex I/II/III/IV of the electron transport chain; DLAT: dihydrolipoamide acetyltransferase; DLD: dihydrolipoamide dehydrogenase; FADH_2_/FAD: flavin adenine dinucleotide; FDX1: ferredoxin 1; GCSH: glycine cleavage system H protein; LIAS: lipoic acid synthetase; LIPT1/2: lipoyltransferase 1/2; NADH/NAD^+^: nicotinamide adenine dinucleotide; PDH: pyruvate dehydrogenase; ROS: reactive oxygen species; TCA: tricarboxylic acid. Molecules were drawn with Marvin, Product version 17.21.0, Chemaxon (https://www.chemaxon.com); Figure was created in BioRender. Scholz, S. (2026) https://BioRender.com/a70cymy

Tsvetkov et al. recently identified a novel molecular mechanism of copper-induced cytotoxicity. Their work demonstrated that excess intracellular copper ions trigger a distinct, apoptosis-independent form of regulated cell death, termed cuproptosis. (Tsvetkov et al. 2022) Herein, the primary molecular target is the pyruvate dehydrogenase (PDH) complex, specifically, the dihydrolipoamide acetyltransferase (DLAT) subunit. Under physiological conditions, DLAT is lipoylated by lipoic acid synthetase (LIAS) as part of PDH maturation. However, in presence of elevated copper levels, lipoylated DLAT undergoes aberrant oligomerisation, leading to disruption of the tricarboxylic acid (TCA) cycle and impairment of proteostasis (Fig. 4). Such DLAT oligomers are likely stabilised by disulfide bonds between the lipoyl-moieties (Tsvetkov et al. 2022), consistent with earlier studies demonstrating that copper catalyses the formation of disulfide bonds. (Zischka et al. 2011; Eben and Imlay 2023; Zubčić et al. 2020).

Copper also interferes with the formation of Fe–S clusters, by binding to Fe–S assembly enzymes, a process that can further compromise proteostasis. (Du et al. 2023) Because Fe–S clusters serve as ubiquitous cofactors for numerous enzymes, the downstream consequences of this disruption are likely multifaceted, which require further investigations. (Vallières et al. 2024) All mammalian Fe–S clusters are formed in mitochondria before being distributed to other cellular compartments. (Lebigot et al. 2017) Within mitochondria, several key enzymes depend on Fe–S clusters, including aconitase, succinate dehydrogenase, and fumarase of the TCA cycle (Li et al. 2017), as well as in complexes I, II, III of the ETC. (Read et al. 2021) Functional effects of copper on the ETC were also assessed by Seahorse experiments. These studies revealed no direct impact on the ATP synthase activity or basal respiration, but demonstrated a reduced spare respiratory capacity, indicating impaired ETC function. These effects are interpreted as secondary to TCA cycle disruption caused by DLAT oligomer formation, rather than a direct effect of copper on the ETC. (Tsvetkov et al. 2022).

Furthermore, the direct effects of copper on DLAT and its indirect effects via interference with Fe–S cluster formation may be mechanistically interconnected. Both ferredoxin 1 (FDX1) and LIAS, which are key enzymes required for of DLAT lipoylation, are themselves Fe–S cluster proteins. (Tsvetkov et al. 2022) However, the relationship of copper with the lipoylation pathway appears more complex than a simple disruption of Fe–S cluster biogenesis (reviewed by Lutsenko et al. 2025). For instance, it was demonstrated that the presence and functionality of FDX1 determines the function of LIAS, by promoting LIAS binding to the lipoyl-group carrier protein, i.e., glycine cleavage system H protein (GCSH), thereby influencing the overall lipoylation pathway. (Dreishpoon et al. 2023) Notably, although functional lipoylation is required for cuproptosis, knockout of *FDX1* – and the subsequent loss of LIAS activity, confers protection against copper toxicity. (Tsvetkov et al. 2022) Copper exposure also reduces the abundance of FDX1 and LIAS protein, raising the question of how a lipoylation-dependent mechanism remains operative under high levels of copper. (Tsvetkov et al. 2022; Du et al. 2023) The proposed model suggests that FDX1-regulated lipoylation enables a toxic-gain-of-function mechanism, however the molecular details of this mechanism remain to be resolved (reviewed by Lutsenko et al. 2025) Despite these uncertainties, current evidence clearly demonstrates that copper directly interferes with the TCA cycle via DLAT oligomerisation and with Fe-S cluster proteins, thereby affecting both TCA cycle function and, directly or indirectly, the ETC. Collectively, these findings highlight cellular energy metabolism as a central target of copper.

While cuproptosis has recently captured much attention as a distinct form of copper-induced cell death, thereby expanded our understanding on copper toxicity mechanisms, it is important also to recognise established molecular mechanisms associated with copper overload. Many of the effects are driven by oxidative stress, characterised by excessive formation of ROS (reviewed by Pizzino et al. 2017). Copper ions participate in Fenton-like reactions that generate highly reactive species such as hydroxyl radicals, which can damage DNA, membranes, lipids, and proteins. (Yang et al. 2019; White et al. 2005; Pradhan et al. 2020) Mitochondria are particularly vulnerable to oxidative injury, as ROS are continuously generated as by-products of oxidative phosphorylation and must be efficiently detoxified by mitochondrial antioxidant systems. (Murphy 2009) Given that cuproptosis also affects mitochondria, these organelles represent a central site in copper-induced toxicity. Beyond oxidative stress-mediated injury and cuproptosis, additional mechanisms have been reported, including proteasome inhibition (Hindo et al. 2009), direct DNA damage by copper complexes (Icsel and Yilmaz 2014), and perturbation of the homeostasis of other essential metals (Doguer et al. 2018). For a more comprehensive coverage of those and further details we refer readers to existing review articles (Gaetke et al. 2003, Sailer et al. 2024, Lutsenko et al. 2025 and Li et al. 2023).

Copper deficiency can also lead to pathological outcomes, reflecting its indispensable role as an essential micronutrient. Numerous enzymes require copper for proper function (reviewed by Schreiber et al. 2013) and insufficient copper availability results in reduced activity of such cuproenzymes and associated molecular impairments. For example, a reduced SOD1 activity caused by copper deficiency weakens cellular antioxidant defences and can consequently contribute to oxidative stress. (Li et al. 2024b).

#### Clinical manifestations

The pathological consequences of copper deficiency are particularly evident in Menkes disease, a genetic disorder caused by mutations of *ATP7A* that impair copper export from the enterocyte into the blood stream (Fig. 3). (Vulpe et al. 1993) As a result, systemic copper deficiency develops already in infants, which is associated with a range of severe symptoms, such as abnormal hair, jaundice, seizures and multiple developmental impairments. (Tümer and Møller 2010) Even with early copper histidine supplementation, most patients die in childhood, underscoring the importance of adequate copper supply. (Fujisawa et al. 2022) Dietary copper deficiency may occur primarily by insufficient intake, or secondary, through antagonistic interactions, such as excessive zinc intake. (Scheiber et al. 2013) Clinical manifestations in these cases are typically less severe than in Menkes disease, reflecting the milder degree of deficiency, and include hematologic and bone abnormalities (reviewed by Halfdanarson et al. 2008 and Zhang et al. 2024a, b).

In contrast, copper overload also leads to serious clinical symptoms and may result from acute or chronic excess copper intake or from genetic disorders. Human data on acute copper intoxication are available from accidental overdoses or suicidal attempts (reviewed by Sailer et al. 2024). Affected individuals commonly experience haemolysis as well as renal and liver injury. (Agarwal et al. 1975) An acute oral dose of 1 g CuSO_4_ can be lethal. (Stein et al. 1976) Chronic excessive copper intake, for example via contaminated drinking water, primarily affects the liver, although anaemia and neurotoxicity have also been described (National Research Council et al. 2000).

Similar to Menkes disease, Wilson disease is also linked with dysfunction of a copper transporter. In this disorder, ATP7B, the transporter responsible for exporting copper from hepatocytes into the bile for excretion, is impaired (Fig. 3). (Bull et al. 1993) As a result, copper accumulates in the liver, frequently leading to chronic liver failure and, in severe cases, the need for liver transplantation. (Schilsky 2014) Neurological manifestation, including tremor, dystonia, rigidity and ataxia, typically appear later in life and are largely reversible upon appropriate treatment (reviewed by Dusek et al. 2019 and Litwin et al. 2017).

## Toxicological relevance and analytical challenges of nanoparticles

As outlined above, copper homeostasis is tightly regulated, and both deficiency and overload can result in adverse effects. However, the mechanisms discussed thus far largely derived from studies using ionic copper. In contrast, copper oxide NPs such as CuO and Cu_2_O may elicit additional or distinct effects arising from their nano-specific properties. These potential differences in behaviour and toxicity are still not fully understood, and despite more than two decades of nanotoxicology research, substantial analytical challenges continue to limit mechanistic insight.

In the following sections, we introduce the broader toxicological relevance of NPs in general and basic principles of nanotoxicology, including the analytical challenges associated with their detection, characterisation, and fate in biological systems. These considerations provide an integrated framework that supports interpretation of the existing evidence base and underpins the subsequent systematic and bibliometric evaluation of copper oxide NPs toxicity.

### Toxicological relevance of nanoparticles

**Fig. 5:**
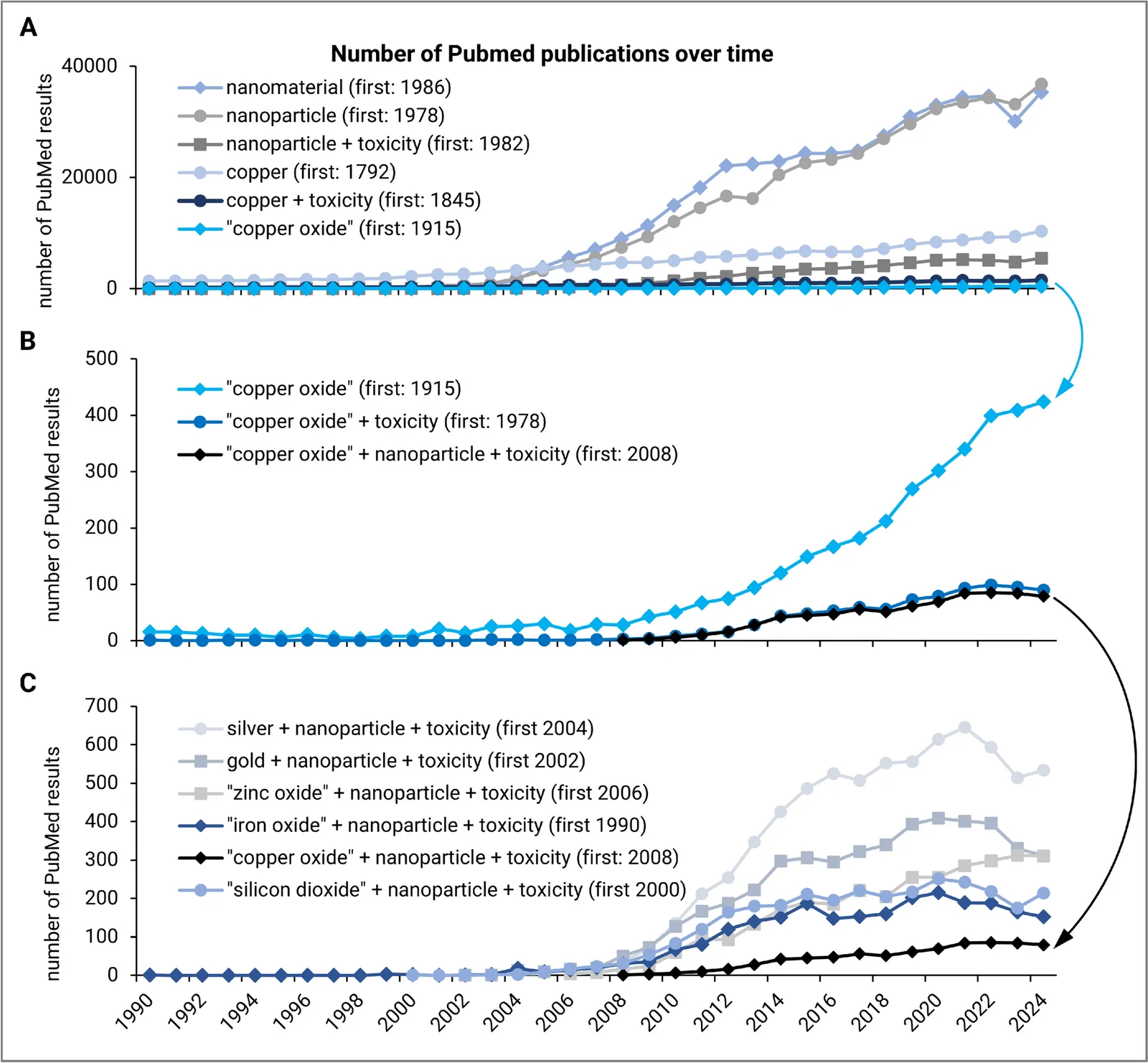
Number of Pubmed publications over time as an indicator on scientific interest in nanomaterial (NM) and nanoparticle (NP) toxicology. **A** Number of PubMed results per year for the search terms “nanomaterial”, “nanoparticle”, “nanoparticle toxicity”, “copper”, “copper toxicity”, and “copper oxide”. **B** Trends in publications using the term “copper oxide” in combination with “toxicity” and “nanoparticle”. **C** Comparison of publication number for various metal and metal oxide nanoparticles in toxicological contexts. All data were extracted from PubMed, May 2025

Starting in the early 2000s, interest in the research of NPs increased markedly, driven by advances in analytical technologies that enabled the detection and characterisation of ever-smaller particles. (Oberdörster et al. 2005) Still, investigations of very small particles existed well before this period, although they appeared under different terminology, such as “colloid research” or “fine particulate matter”. (Wallace 1996) Definitions of NPs have also evolved over time. The most recent European definition, provided by Rauscher et al. 2022, classifies a material as a NM, when at least 50% of its particles have at least one external dimension smaller than 100 nm (see section EU regulation of copper, copper oxides, and their nanoforms) (EC 2022). Trends in scientific focus are reflected in the number of PubMed results for different search terms over the years (Fig. 5). Approximately six years after the rise in publications about NPs, a clear increase in studies addressing NPs toxicity becomes evident (Fig. 5A). While research interest in NPs and their toxicological properties remains high, publication number for some metal NPs have begun to decline. An exception are copper oxide NPs, which remain among the least studied metal NPs and show a relatively stable number of publications per year (Fig. 5 C).

NPs exhibit distinct physicochemical properties, such as altered melting points, fluorescence, electrical conductivity, and surface reactivity compared with the same materials in bulk form or, for metals, with their ionic counterparts. (Asha and Narain 2020) Even the colour of a material may differ, a well-known example is the use of gold NPs to impart a red tint to glass. (Ruivo et al. 2008) These specific characteristics have supported their growing use across various industries, for example as catalysts or as coatings for textiles (reviewed by Malik et al. 2023). As noted above for CuO and Cu_2_O, NPs can also arise unintentionally or originate from natural sources (Raut et al. 2024).

The unique physicochemical properties of NPs may also result in distinct toxicological outcomes. For example, metal-derived ions can generate ROS and contribute to oxidative stress, as described for copper above. Because NPs have a higher surface-to-volume ratio, their rate and extent of ion release, can be greater, potentially enhancing oxidative stress. (Hedberg et al. 2019) Another important difference concerns the likely route of exposure. Unlike fluids or bulk metals, NPs are typically handled as powders, making inhalation a plausible route of exposure. This contributed to an early emphasis on pulmonary toxicity within nanotoxicology research (Oberdörster 2000).

Oral exposure to NPs, which is in the focus of this review, can also lead to distinct effects. It has been shown that NPs can be internalised by cells through endocytosis, phagocytosis or macropinocytosis and can also be exocytosed as intact particles (reviewed by Oh et al. 2014). These uptake and transport mechanisms are relevant for the intestine, which has a large surface area and a high rate of NP translocalisation (reviewed by Fröhlich et al. 2016). Robust evidence indicates that, under certain conditions, orally ingested NPs can be taken up by intestinal cells and subsequently distributed systemically to sensitive tissues (reviewed by Krug et al. 2014).

### Analytical challenges in nanotoxicology

Studying the toxicity of NPs presents specific challenges, both in particle characterisation and in selecting appropriate assays to measure effects, especially when applying NAMs. Comprehensive characterisation requires multiple analytical approaches. Beyond determining particle size and shape, parameters such as surface charge, chemical composition, solubility, dissolution and agglomeration behaviour must also be assessed. (Fadeel et al. 2017) To address these needs, a broad range of analytical techniques has been developed or adapted, including transmission electron microscopy (TEM) (Verleysen et al. 2019), scanning electron microscopy (SEM) (Crouzier et al. 2019), dynamic light scattering (DLS) (Braun et al. 2011), nanoparticle tracking analysis (NTA) (Hole et al. 2013), small angle X-ray scattering (SAXS) (Pauw et al. 2017), and single particle inductively coupled plasma mass spectrometry (spICP-MS) (Loeschner et al. 2013).

Each of these methods offers specific strengths and faces distinct limitations. TEM provides exceptional spatial resolution down to the sub-nanometre scale making it one of the most widely used techniques for NPs imaging (reviewed by Aziz et al. 2025). In complex biological matrices, however, TEM often requires complementary techniques such as energy-dispersive X-ray spectroscopy (EDX) to obtain elemental information. (Zheng et al. 2010) A further limitation is that TEM provides limited insight into surface morphology, which can be analysed more effectively with SEM, albeit with substantially lower resolution (reviewed by Aziz et al. 2025). For determining the hydrodynamic diameter of NPs in liquid suspension, DLS or NTA are the preferred methods, although their accuracy decreases with polydispersity and sample complexity increase. (Hole et al. 2013).

Furthermore, NPs may undergo transformation, such as protein corona formation, development of organic and hydrate layers, or partial dissolution. These processes depend strongly on the surrounding environment, including conditions encountered in different regions of the gastrointestinal tract, which adds further complexity to analytical characterisation. (Böhmert et al. 2020; Bove et al. 2017; Monopoli et al. 2020) Such dynamic interactions can alter the apparent particle size, surface charge, and bioavailability. Consequently, it is important to assess both the core size (e.g., by TEM) and the hydrodynamic diameter (e.g., via DLS) and to compare these measurements. (Filippov et al. 2023) When designing toxicological studies, additional considerations are required, especially when using NAMs. Even with an optimised dispersion protocol to ensure uniform NP application at the beginning of an in vitro experiment, particles may agglomerate, sediment, dissolve, or become inaccessible due to size-dependent diffusion limitations during the exposure period. (Teeguarden et al. 2007) Therefore, characterising NPs over time in relevant media and accounting for dosimetry is essential. Dosimetric approaches provide a more accurate description of NP exposure in vitro by considering particle number, surface area, and mass. (Böhmert et al. 2018; DeLoid et al. 2017) Accurate and reliable dosimetry is essential for understanding the actual cellular exposure and for interpreting results from in vitro toxicity studies.

Finally, results from effect-based toxicological assays must be evaluated carefully and interpreted with caution, as NPs can interfere with several commonly used endpoints (reviewed by Ong et al. 2014). Such interferences have been reported for numerous assays, including, luminescence-based assays (Lehmann et al. 2016), the lactate dehydrogenase (LDH) assay (Han et al. 2011), the 3-(4,5-dimethylthiazol-2-yl)-2,5-diphenyltetrazolium bromide (MTT) assay (Holder et al. 2012), the comet assay (Ferraro et al. 2016), and even standard methods such as RT-qPCR (Sanabria and Gulumian 2017). Although some studies provide strategies to mitigate such effects, assay interference remains an additional analytical challenge to nanotoxicology research.

### Systematic literature analysis on copper oxide NP toxicology

After addressing the first key question, i.e., (i) whether CuO and Cu_2_O NPs are sufficiently covered by EU regulations to prevent unintentional oral exposure via the food and feed chain, and setting the stage for the subsequent parts by introducing general aspects of copper exposure, homeostasis, and toxicity, we now turn to a systematic and bibliometric evaluation of the available toxicological literature. This analysis serves as the foundation for addressing the remaining two key questions: (ii) whether CuO and Cu_2_O NPs are capable of crossing the intestinal barrier as intact particles and subsequently accumulating in sensitive tissues, and (iii) whether these NPs elicit distinct biological responses, including specific cell death mechanisms such as cuproptosis.

### Bibliometric analysis

#### Formal analysis of the dataset

We conducted a systematic review on the toxicity of CuO and Cu_2_O NPs using a comprehensive search string that combined relevant synonyms for “nanoparticles” with terms for CuO and Cu_2_O and toxicological endpoints (Supplementary Table 2). Searches were performed in four major databases, yielding 649 relevant studies that were analysed in detail (see Methods section in Supplementary Information and Supplementary Excel file).

**Fig. 6:**
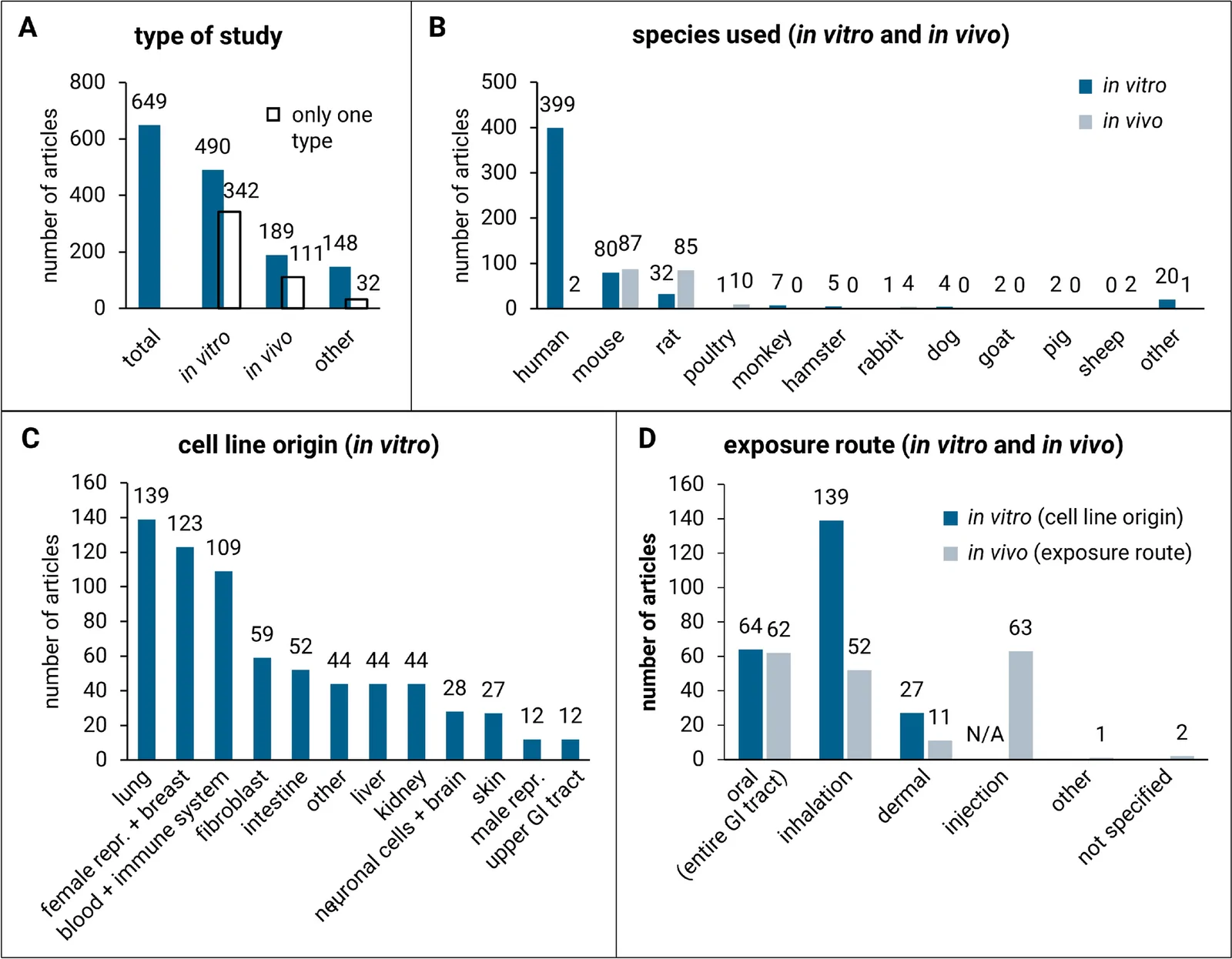
Overview of study types and experimental models used in articles investigating the effects of CuO or Cu_2_O nanoparticles. **A** A total of 649 articles were categorised based on the use of in vitro, in vivo, and other approaches. Most of the articles employed only one type of study (open black bars). **B** Comparison of species used in in vitro and in in vivo experiments. **C** Categorisation of the organ origin of the cell line. **D** Comparison of investigated exposure routes in in vitro and in in vivo experiments. GI: gastrointestinal N/A: not applicable; repr.: reproductive system; the category “blood” also includes cells of the vascular system

Firstly, we examined the types of studies and experimental models used (Fig. 6). The categorisation by study type showed that most research on CuO and Cu_2_O NPs was conducted in in vitro models accounting for 76% (490/649) of the articles (Fig. 6A). In addition, 29% (189/649) of the articles included in vivo analyses, and 23% (148/649) employed methods that do not fall into either category but were still relevant for assessing CuO and Cu_2_O NPs toxicity, such as investigating NPs behaviour in fluids or interactions with other molecules. Overall, 75% (485/649) of the articles focused exclusively on in vitro, in vivo, or other methods, while 25% (164/649) combined two or all three approaches.

Most in vitro experiments used human cells (81%, 399/490), whereas in vivo studies predominantly used mice (46%, 87/189) and rats (45%, 85/189) (Fig. 6 B). Other species were used only to a limited extent, with poultry being the only additional species with notable usage in in vivo experiments (5%, 10/189). The scarcity of studies in other species limits the ability to draw conclusions regarding potential effects in farm animals and the transfer of CuO or Cu_2_O NPs into meat or other animal products. However, given the use of CuO and Cu_2_O in feed additives, as introduced above, this remains a relevant area of investigation.

We also analysed the representation of organs and exposure routes. In in vitro studies, the respiratory system (28%, 139/490), female reproductive/breast tissue (25%, 123/490), and blood/immune cells (22%, 109/490) were investigated most frequently and to a similar extent. The fewest articles focused on male reproductive tissue or the upper GIT , including mouth oesophagus, and stomach (Fig. 6C). The high use of cells originating from the respiratory system is a known characteristic of NP toxicity research, in contrast to toxicity research in general. (Ivask et al. 2015) In our analysis, the respiratory system was also the most frequently investigated exposure route in vitro, whereas in vivo studies showed a more balanced distribution of exposure routes. The only notable exception was dermal exposure in vivo, which was examined in only 6% of the articles (11/189) (Fig. 6D). These differences may reflect the general relevance of inhalation exposure to NPs, contrasted with the technical challenges associated with in vivo inhalation studies. However, in this review the primary focus was on the oral exposure, as this represents the main exposure route of CuO and Cu_2_O NPs for the general population as described above.

**Fig. 7:**
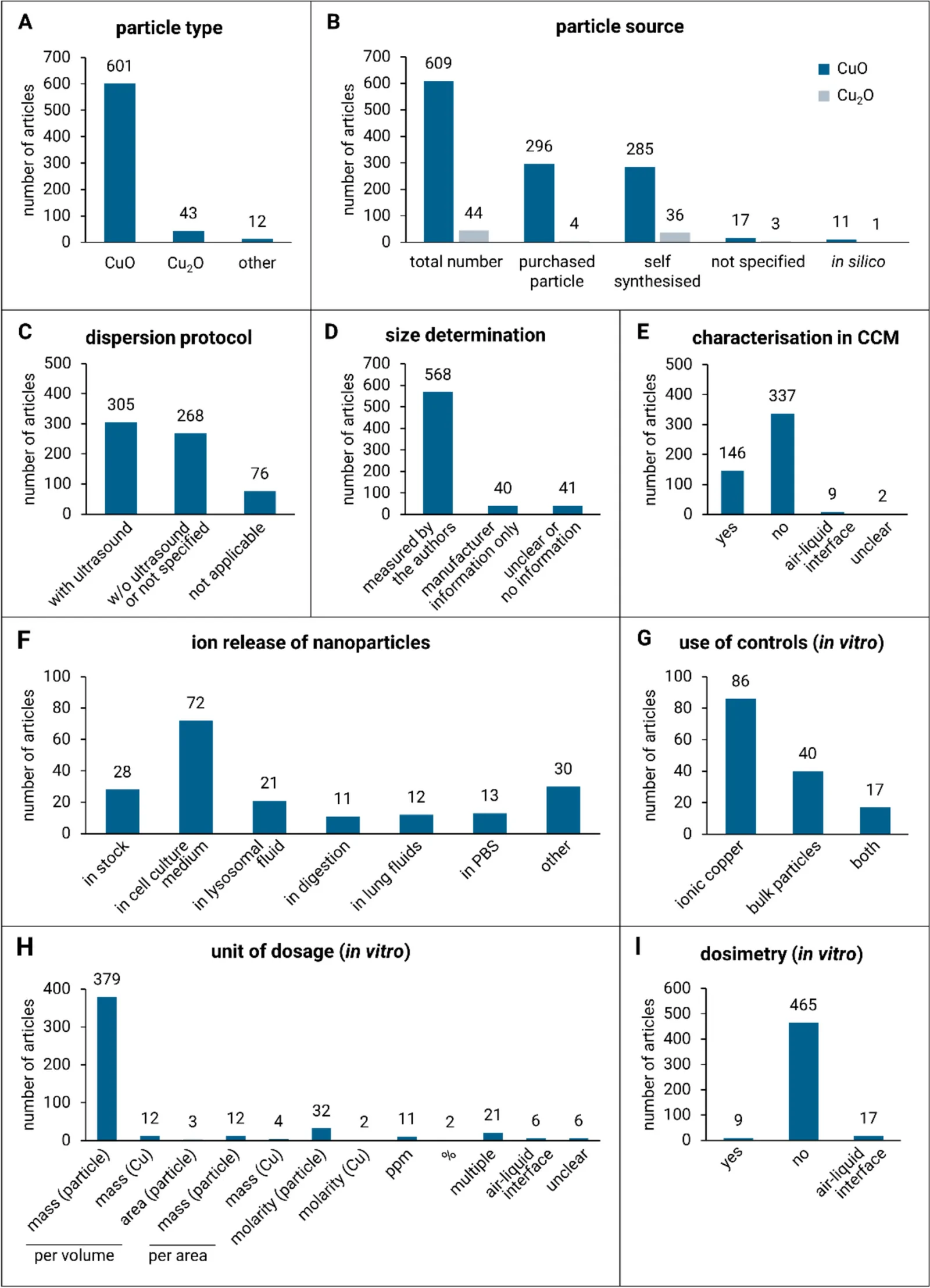
Overview of article distributions on particle type and characterisation methods, quality control measures, and dosimetric considerations in studies investigating effects of CuO or Cu_2_O nanoparticles (NPs). **A** Distribution of articles analysing different types of NPs. **B** Distribution according to the source of NPs by oxidation state. **C** Distribution of articles reporting the use of ultrasound in the dispersion protocol. **D** Distribution of articles reporting methods for NP size determination. **E** Distribution of articles addressing particle characterisation in cell culture media. **F** Article distribution on ion release measurements in different media. **G** Distribution of articles describing the types of used controls in in vitro experiments **H** Distribution of articles reporting unit of dosage in in vitro experiments per volume of CCM or per area of cell culture dish **I** Distribution of articles applying dosimetric calculations for in vitro experiments. CCM: cell culture media; PBS: phosphate buffered saline; w/o: without

Next, we described the types of NPs used, their physiochemical characterisation, quality control measures, and dosimetric considerations, all of which are essential for interpreting the toxicological outcomes reported in the reviewed studies (Fig. 7). Most articles (93%, 601/649) investigated CuO NPs, whereas only 7% (43/649) focused on Cu_2_O (Fig. 7A). Consequently, the current body of research predominantly addresses the effects of CuO NPs while data on Cu_2_O NPs remain limited. A small number of articles fell into the “other” category, which includes particles that do not strictly meet the criteria for CuO or Cu_2_O, but are still relevant due to close chemical or structural similarity. Thus, these include mixed oxidation-state formulations and particles with functional coatings, such as gold-core/copper oxide shells. Their limited presence reflects the specificity of the search strategy.

For CuO, studies were evenly divided between commercially obtained materials (49%, 296/609) and particles synthesised by the authors (47%, 285/609) (Fig. 7 B). In contrast, most Cu_2_O particles were synthesised in-house (82%, 36/44) with only four studies relying on commercial sources. A small number of publications did not specify the particle source, and twelve studies (< 2%, 12/649) conducted exclusively in silico modelling without the use of physical particles. The choice between commercial and synthesised particles may affect reproducibility and comparability, as synthesis procedures can yield particles with divergent physicochemical characteristics, including variations in size or morphology.

Because NPs need to be homogeneously dispersed in liquid media for most biological assays, we analysed dispersion methods reported in the literature. Particular attention was given to the use of ultrasound, as it is known to substantially reduce agglomeration and is recommended for NP dispersion. (Hartmann et al. 2015) Overall, 47% of the studies (305/649) reported the use of ultrasound for dispersion (Fig. 7 C). In contrast, 41% (268/649) either did not use ultrasound or did not specify their approach used. For 12% of the studies (76/649), a dispersion protocol was not applicable, e.g., due to the nature of the experimental setup, such as non-liquid exposure systems. These results indicate that data compatibility across studies is limited and that interpretations should be made cautiously. The analysis of this category was further complicated by frequent interchangeable use of the terms “dissolved” and “dispersed” despite their distinct physical meanings. Thus, dispersed particles retain their nanoscale nature, while dissolved particles release ions and no longer maintain particulate properties. (EFSA 2021).

The analysis of articles with regard to the particle characterisation focused on a basic size characterisation and the evaluation of ion release in different fluids only, even though relevant guidances specify a broader set of characterisation. (EFSA 2021) A basic characterisation was performed in 88% (568/649) of the studies, typically using TEM, DLS or other appropriate methods (Fig. 7D). Only 6% (40/649) relied exclusively on manufacturer specifications, the reliability of which can vary. Another 6% (41/649) either did not report their approach or provided insufficient details to allow a solid conclusion. Among studies conducting in vitro experiments, only 30% (146/490) also characterised their particles in cell culture media (CCM) (Fig. 7E). This represents a critical but often neglected aspect, as NP properties can change substantially in biological matrices due to processes such as protein corona formation, pH driven alterations, agglomeration, or dissolution as introduced. (Böhmert et al. 2020; Bove et al. 2017; Monopoli et al. 2020).

With respect to dissolution and copper ion release, we identified 29% of articles (187/649) that included an assessment of these processes in various fluids (Fig. 7F). Most commonly, ion release was examined in CCM, followed by stock dispersions and lysosomal fluid. Additional matrices, such as phagolysosomal simulant fluid, simulated body fluid, serum, artificial interstitial fluid, and synthetic sweat, were also used and are grouped under “other”. Because NPs differ substantially in concentration, size, shape and agglomeration behaviour, the results from studies characterising particles or ion release in CCM in other fluids cannot be directly compared or transferred across systems. Nevertheless, the number of available studies and the broad range of tested fluids provide a useful basis for identifying most relevant conditions for future assessment.

The release of copper ions from CuO or Cu_2_O NPs is an important determinant of their toxicity, because copper ions themselves contribute to adverse effects as reviewed above. For this reason, the use of an ionic copper control, which is matched to the copper mass applied via the NPs, is advisable together with the measurement of ion release. Likewise, the inclusion of non-nano particle (i.e., bulk particle) control, can help differentiate nano-specific effects from other particle-related influences, such as mechanical stress. Overall, only 13% of articles (86/649) included ionic controls, 6% (40/649) used bulk particle controls, and 3% (17/649) applied both types of controls in at least in one experiment (Fig. 7G). Consequently, in the majority of the studies the observed effects cannot be clearly attributed to particle-specific or non-nano specific mechanisms, which substantially limits the interpretability of the respective findings.

Dosing is another critical aspect of experimental design, particularly in in vitro studies, where particle sedimentation over time complicates exposure conditions. Among the articles using in vitro methods, 77% (379/490) report the dose as particle mass per incubation volume (Fig. 7H). However, most articles did not provide the incubation volume or well-plate-format, information needed to convert dosing to surface-area basis and enable comparison across studies. Considering the role of copper ions in toxicity, quantifying the actual copper mass and adjusting concentrations accordingly would also be essential. This was performed in only 2% of articles (12/490) on a volume basis and in < 1% (4/490) on a surface-area basis. Previous work has demonstrated that the elemental metal content of NPs can differ markedly from values calculated based on chemical formula. (Singh et al. 2019; Nouren et al. 2024) As a result, much of the available CuO and Cu_2_O NP toxicity data and its interpretation, may be biased due to the lack of consideration of actual copper content. The current state-of-the-art approach for expressing NP dose involves dosimetric calculations that account for sedimentation, effective density and diffusion to estimate the fraction of particles actually reaching the cell surface over time. Only 2% (9/490) of the in vitro studies applied such dosimetric calculations (Fig. 7 I). Although such approaches provide a more realistic basis for interpreting cellular exposure and toxicities, they remain rarely used due to their methodological complexity.

**Fig. 8:**
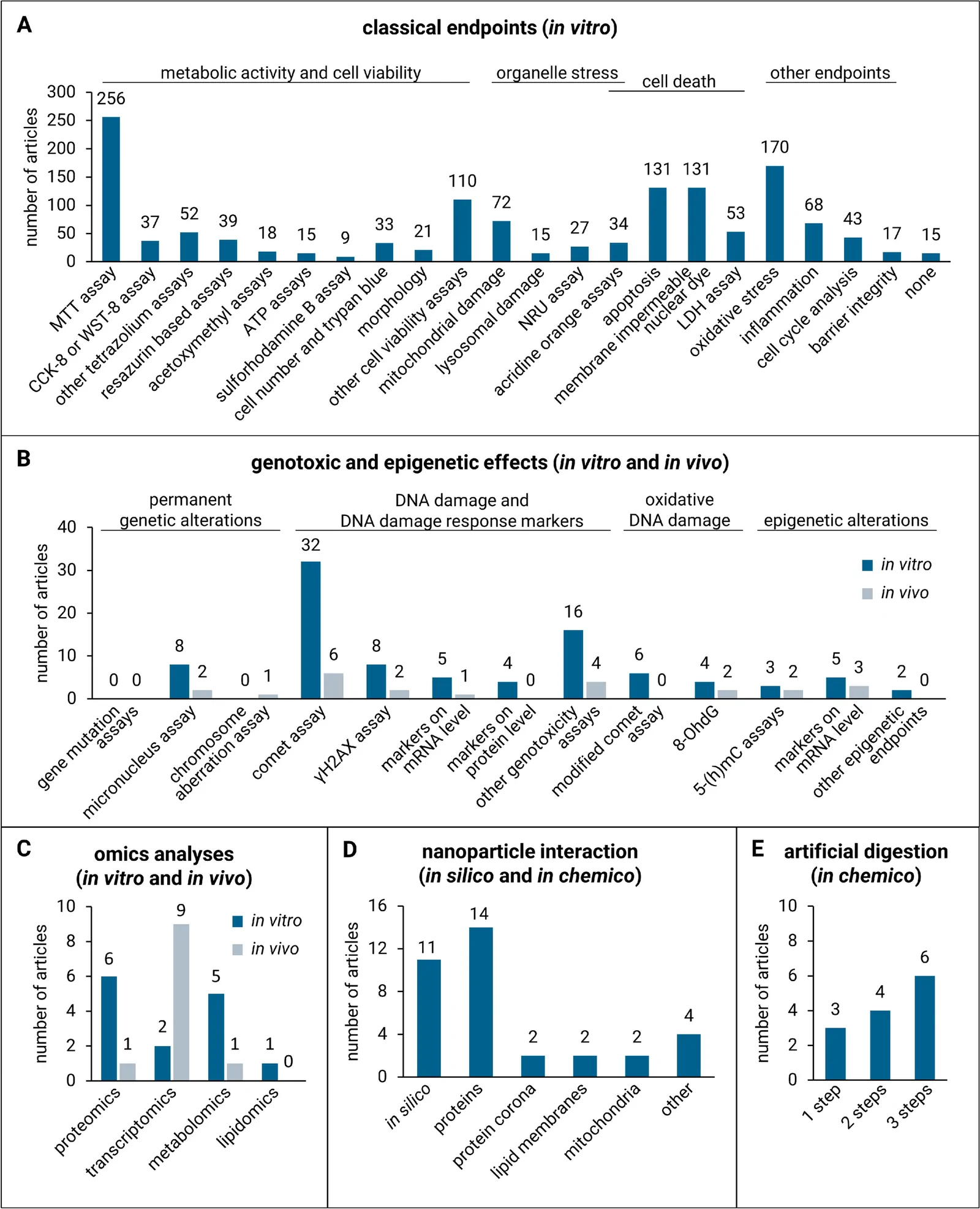
Distribution of articles across endpoint categories in studies investigating effects of CuO or Cu_2_O nanoparticles **A** Distribution of articles using classical in vitro toxicological endpoints, grouped into metabolic and cell viability assays, organelle stress, cell death and other endpoints. **B** Distribution of articles applying assays for permanent genetic alterations, DNA damage and DNA damage response markers, oxidative DNA damage, or epigenetic alterations in in vitro and in vivo studies. **C** Distribution of articles employing omics approaches in in vitro and in vivo experiments. **D** Distribution of articles investigating nanoparticle interactions with molecules or biological structures in in silico and in chemico experiments. **E** Distribution of articles assessing effects of artificial digestion by one-step, two-step, or three-step in in chemico experiments. 5-(h)mC: DNA methylation or hydroxymethylation; 8-OhdG: 8-oxo-7,8-dihydro-2′-deoxyguanosine; ATP: adenosine triphosphate; CCK-8: cell counting kit 8; LDH: lactate dehydrogenase; MTT: 3-(4,5-dimethylthiazol-2-yl)-2,5-diphenyltetrazolium bromide; NRU: neural red uptake; WST-8: water-soluble tetrazolium 8

We additionally analysed the endpoints assessed in the included articles. The analysis focused on the types of cell viability assays and other commonly analysed in vitro endpoints, the types of genotoxicity assays, and the use of omics approaches in both, in vitro and in vivo studies, as well as particle interaction and artificial digestion in studies classified under “other” (Fig. 8). Because the mechanisms underlying CuO and Cu_2_O NPs-induced toxicity for classical endpoints have been reviewed extensively in recent years, our evaluation was limited to describing the available literature and identifying data gaps (reviewed by Sajjad et al. 2023 and Naz et al. 2020 and Ameh et al. 2019).

Among metabolic activity assays, the MTT assay was the most frequently used, as applied in 52% of the articles using in vitro methods (256/490) (Fig. 8A). Furthermore, 8% (37/490) of articles used the water-soluble tetrazolium 8 (WST-8) or cell counting kit 8 (CCK-8) assay, which differ in naming but rely on the same underlying chemistry. A further 11% (52/490) employed other tetrazolium-based assays, including the 3-(4,5-dimethylthiazol-2-yl)-5-(3-carboxymethoxyphenyl)-2-(4-sulfophenyl)-2H-tetrazolium (MTS) or water-soluble tetrazolium 1 (WST-1) assays. However, several groups have reported that copper interferes with formazan salts, the key metabolic product quantified in these assays. (Semisch and Hartwig 2014; Perez et al. 2017) Given that the tetrazolium-based assays were the most abundantly used type of assay, the available research on CuO and Cu_2_O NPs may be affected by artefacts arising from copper-induced interference with formazan formation and therefore affecting cell viability data. Other established metabolic activity assays, such as resazurin- or acetoxymethyl-based assays were used in 8% (39/490) and 4% (18/490) of the articles, respectively. Both have been reported not to be influenced by CuO NPs, rendering them preferable alternatives, however the choice of fluorescent conjugate of acetoxymethyl-based assays requires careful considerations. (Galbis-Martínez et al. 2018; Gupta et al. 2022) We also identified several additional assays to assess cell viability, including ATP quantification, sulforhodamine B staining, cell counting, and trypan blue exclusion, and morphological characterisations. Additionally, 22% of the articles (110/490) employed other assays or methods that provide information on cellular health but did not fit into any of the predefined categories. Among those were colony forming assays, cellular impedance-based measurements, skin sensitisation models, cell differentiation assays, patch clamp recording, wound healing assays, and cell migration assays.

We also identified 15% of the articles (72/490) that specifically investigated mitochondrial effects, using approaches such as JC-1 or Rhodamine 123 staining, analysis of markers including PTEN-induced kinase 1 (PINK1), or measurements of cellular oxygen consumption. Lysosomal damage was assessed by 3% of the articles (15/490) by methods such as LysoTracker or as analysis of lysosome-associated membrane protein 1 (LAMP1), and 6% (27/490) used the neutral red uptake (NRU) assay. In addition, 7% of articles (34/490) employed acridine orange staining, which can indicate either lysosomal membrane integrity or apoptotic processes, depending on the specific experimental set-up—(N.B. these studies were not categorised further).

We further analysed the articles for assessment of general cell death markers. Apoptosis was investigated in 27% (131/490) of the articles, using approaches such as caspase activity assays, DNA fragmentation analysis, or the evaluation of markers including Bcl-2-associated X protein (BAX) and apoptotic protease-activating factor 1 (APAF1). Loss of membrane integrity, which is used as a marker of necrosis, was assessed in 27% of the articles (131/490) through membrane impermeable nuclear dyes, while 11% (53/490) performed an LDH assay. Notably, potential interference of CuO NPs with the LDH assay has been reported. (Boyadzhiev et al. 2021) Other endpoints that were assessed included oxidative stress (35%, 170/490), inflammation (14%, 68/490), cell cycle alterations (9%, 43/490) and barrier integrity (3%, 17/490). A small portion of the studies (3%, 15/490) performed in vitro experiments that did not assess any of these endpoint categories.

Genotoxicity represents another key endpoint, as it is central to the risk assessment of physical and chemical agents, including CuO and Cu_2_O NPs. We examined how this endpoint is addressed in the literature in more detail in both, in vitro and in vivo studies, with a particular focus on the use of OECD test guidelines (TGs), given their regulatory relevance (Fig. 8B). Genotoxicity is assessed through three primary endpoints, i.e. (i) small gene mutations, (ii) structural chromosomal aberrations, and (iii) numerical chromosomal aberrations. Since no single test covers all three endpoints simultaneously, a test battery is required to comprehensively evaluate the genotoxic potential of CuO or Cu_2_O NPs. (EFSA 2021).

Base mutations such as insertion-deletion mutations (i.e., indels) and single base substitutions (i.e., SBS) can be detected using several OECD TG methods, including the in vitro bacterial reverse gene mutation assay (“Ames test”, OECD TG 471), the in vitro mammalian cell gene mutation assays (OECD TG 476 and 490), the in vivo Pig-a gene mutation assay (OECD TG 470), and the in vivo transgenic rodent assay (OECD TG 488). The bacterial reverse gene mutation assay is generally *not* recommended for NP testing because bacterial cells show limited capacity to internalise NPs and NPs may exert bactericidal effects. (Woodruff et al. 2012; Verdon et al. 2022) Additional approaches to detect gene mutations such as in vitro and in vivo error-corrected next-generation sequencing, in vitro Pig-a gene mutation assay and in vitro transgenic gene mutation assays are under development but are not yet covered by an OECD TG. (Rees et al. 2017; White et al. 2019; Marchetti et al. 2023) Structural chromosome aberrations can be detected using the in vitro and in vivo chromosome aberration assay (OECD 473 & 475) and the in vitro and in vivo micronucleus assays (OECD TG 487 & 474). The micronucleus assay offers an advantage over the chromosome aberration test as it can detect numerical chromosome as well as structural chromosomal aberrations. (OECD, 2016c, OECD 2016b) These OECD TGs were developed for conventional chemicals and nano-specific adaptations are recommended for NPs testing. However, such adaptations have so far not been included in the TGs themselves, including OECD TG 487 and OECD TG 473. (Gutleb et al. 2025, OECD 2016a, c) Yet, for the in vitro micronucleus assay (OECD TG 487) a preliminary guidance document for NP-specific adaptations is available. (OECD 2022).

Assays that detect “primary DNA damage and DNA damage response markers” (i.e., DNA lesions that may not necessarily result into permanent genetic alterations), are also available. These indicator tests include the in vitro and in vivo comet assay, the γH2AX assay, and the ToxTracker assay. (García-Rodríguez et al. 2019; Karlsson et al. 2014) An OECD TG exists only for the in vivo comet assay (OECD TG 489), although TG development efforts for several of these approaches are ongoing at the OECD level. Of note, also interferences of NPs with the comet assay are reported, leading to false positive results when no adjustments are made to the protocol. (Ferraro et al. 2016).

In this review, 12% of the in vitro studies (58/490) and 9% of the in vivo studies (17/189) evaluated genotoxicity of CuO and Cu_2_O NPs. Several articles reported results from more than one in vitro and/or in vivo assays. None of the reviewed articles investigated the induction of gene mutations following exposure to CuO or Cu_2_O NPs. In contrast, several studies examined their potential to induce chromosomal aberrations. The micronucleus assay was used in 14% of the in vitro studies (8/58) and 12% of the in vivo studies (2/17). The chromosome aberration assay was applied only once, in a single in vivo study (6%, 1/17). Among the methods assessing DNA strand breaks or alkali-labile sites, the comet assay was the most frequently used approach in both in vitro and in vivo studies (55%, 32/58 and 35%, 6/17). Detection of the DNA damage response marker γH2AX was the second most common method representing 14% of the in vitro studies (8/58) and 12% of the in vivo studies (2/17). Additionally, DNA damage response markers were investigated only in in vitro studies using Western blotting—they accounted for 7% of the in vitro studies (4/58).

Because CuO and Cu_2_O NPs are likely to induce DNA damage through oxidative stress, an enzyme-modified comet assay, incorporating formamidopyrimidine DNA glycosylase (Fpg) or endonuclease III (Endo-III), was used in 10% of the in vitro studies (6/58). The oxidative DNA damage marker 8-oxo-7,8-dihydro-2′-deoxyguanosine (8-OhdG) was analysed in 7% of the in vitro studies (4/58) and 12% of the in vivo studies (2/17). Gene expression analysis of DNA damage-related genes was included in 9% of the in vitro studies (5/58) and 6% of the in vivo studies (1/17). The remaining 28% of the in vitro (16/58) and 24% of the in vivo (4/17) studies employed less commonly used genotoxicity assays, such as the alkaline unwinding assay (Hartwig et al. 1993) and the random amplified polymorphic DNA (RAPD) assay. (Katsnelson et al. 2007) (Katsnelson et al. 2009) Among studies using assays covered by OECD TGs only 18% (3/17) explicitly stated that the guideline was followed. Several of the reviewed studies also examined epigenetic alterations associated with CuO and Cu_2_O NP exposure. These included analysis of DNA methylation (5-mC) and hydroxymethylation (5-hmC), conducted in 5% of the in vitro studies (3/58) and 12% of the in vivo studies (2/17) as well as gene expression analyses related to epigenetic regulation, performed in 9% of the in vitro studies (5/58) and 18% of the in vivo studies (3/17).

Omics approaches are valuable tools for elucidating mechanisms underlying toxic outcomes. In the articles included in this review, such approaches were applied in 3% of the in vitro studies (14/490) and 6% of the in vivo studies (11/189) (Fig. 8C). Proteomics was the most commonly used omics approach used in vitro, whereas transcriptomics predominated in vivo. Among the in vitro studies, five included ionic controls in their omics analysis, all of those articles focused on CuO NPs. Three studies applied microarray-based transcriptomics (Jang et al. 2016; Hanagata et al. 2011; Boyadzhiev et al. 2021) and two used 2D electrophoresis for proteomic profiling (Triboulet et al. 2013, 2015). These studies provide opportunities to compare mechanistic differences between ionic copper and CuO NP toxicity. In contrast, none of the in vivo studies employing omics approaches included ionic copper controls, limiting the ability to distinguish effects specific to the NP form.

In 23% of the articles (148/649), approaches other than classical in vitro or in vivo methods were used, including in silico or in chemico analyses of particle interaction (Fig. 8D). Among these, nine studies used in silico molecular docking, and two relied on other theoretical calculations. Within the in chemico category, studies most frequently examined particle-proteins interaction, commonly involving bovine serum albumin (BSA) or specific proteins such as β-galactosidase. We also evaluated the use of artificial digestion methods for CuO and Cu_2_O NPs, which were described in 13 articles (Fig. 8E). In these experiments, particles were exposed to gastrointestinal-like conditions using relevant enzymes and specific pH levels. Dissolution and other physical chemical properties are typically assessed to investigate the fate of particles throughout the GIT. (Ferraris et al. 2023; Sieg et al. 2017) Most of the artificial digestion studies followed a three step approach encompassing the oral, gastric, and intestinal phases.

In this first bibliometric part of the systematic review, we have shown that a substantial number of articles address a broad spectrum of toxicological relevant endpoints. However, many studies lack a comprehensive particle characterisation and appropriate controls, which limits the reliability of their findings. Additionally, a notable data gap remains for Cu_2_O NPs.

### Content analysis regarding cellular uptake and barrier crossing of copper NPs

**Fig. 9:**
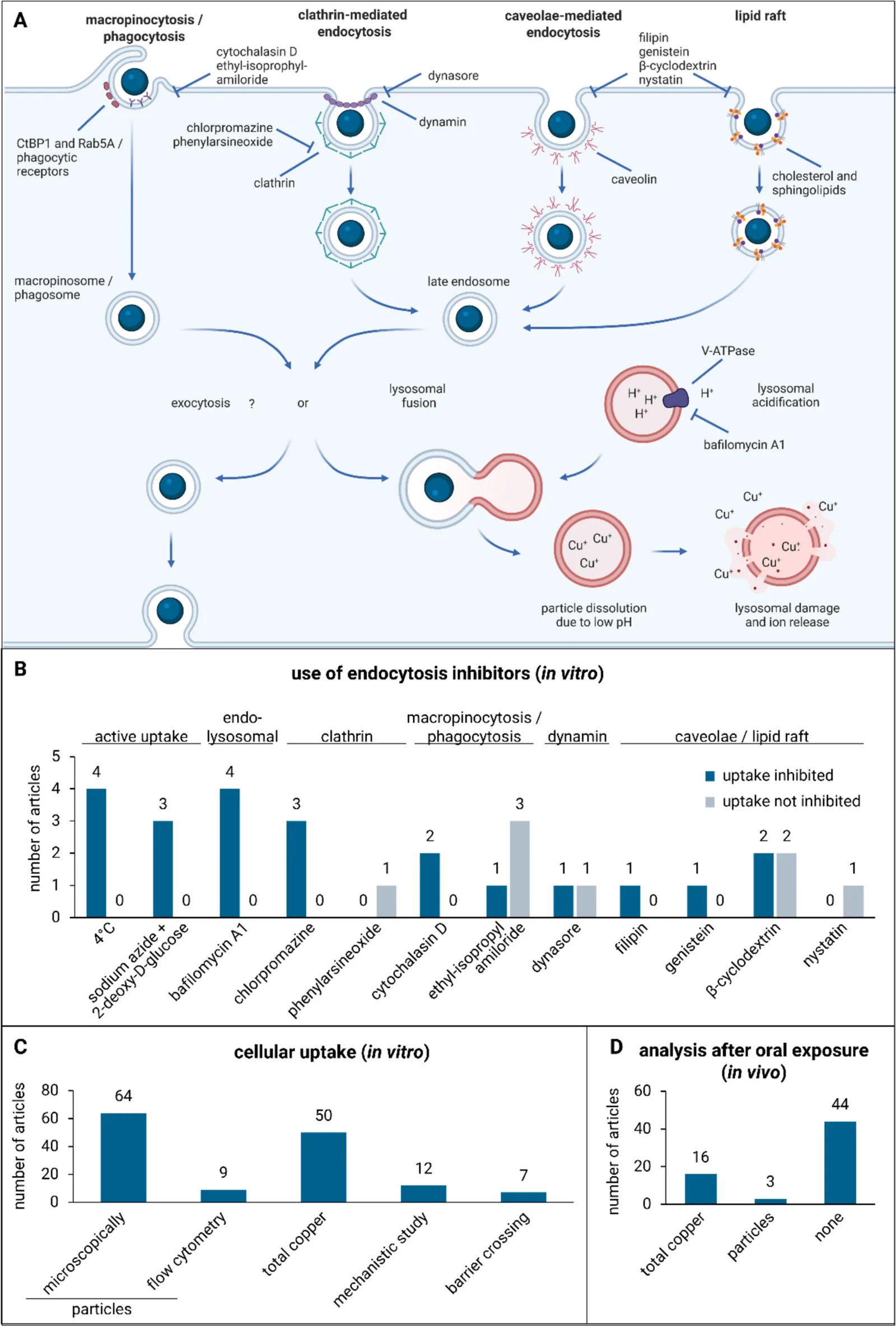
Overview of cellular uptake mechanisms and barrier translocation of CuO or Cu_2_O nanoparticles. **A** Schematic representation of macropinocytosis / phagocytosis, clathrin-mediated endocytosis, caveolae-mediated endocytosis, lipids raft-associated uptake and lysosomal processing, including commonly used chemical inhibitors. **B** Overview evaluation of twelve studies investigating uptake pathways in vitro; multiple inhibitors were often applied within a single study. **C** Overview of number of studies assessing cellular uptake in vitro*,* measuring either particle-associated or total copper. **D** Overview of number of studies assessing cellular uptake in vivo, measuring either particle-associated or total copper. CtBP1: C-terminal binding protein 1; Rab5A: member RAS oncogene family. Panel A was created in BioRender. Scholz, S. (2026) https://BioRender.com/954ybe3

Following the bibliometric overview of existing toxicity data for CuO and Cu_2_O NPs and the identification of overarching data gaps, we address the second key question of this review: (ii) Can CuO and Cu₂O NPs cross the intestinal barrier as intact particles and accumulate in sensitive tissues? To this end, we examined several aspects of particle uptake and barrier translocation. We first evaluated, whether biologically plausible mechanisms for cellular uptake of CuO and Cu_2_O NPs have been described. For this purpose, we analysed studies that assessed cellular uptake in vitro using various chemical inhibitors or low temperature conditions to elucidate active endocytotic processes (Fig. 9 A, B and Table 2). Of the twelve articles identified, only two investigated Cu_2_O NPs, therefore, the available evidence is only sufficient to draw conclusions for CuO NPs.

**Table 2:** Overview of articles investigating endocytosis or active cellular uptake of CuO and Cu_2_O nanoparticles (NPs)

Study	Cell line	Type of copper oxide nanomaterials	Exposure scenario	Type of effector	Selected results	Interpretation
(Wang et al. 2012)	A549 (Human, adenocarcinoma, alveolar basal epithelia)	CuO NPs, purchased, self-characterised, < 100 nm (TEM, sonicated in ethanol)	CuO NPs: 5 and 15 mg/L Cu^2+^: 2.5 mg/L 12 h	NaN_3_ (10 mM) with 2DG (50 mM)	CuO NPs: Intracellular copper is significantly lower in cells treated with inhibitors than in control cells (GF-AAS) Cu^2+^: Intracellular copper in cells treated with inhibitor is equal to control cells (GF-AAS)	Active uptake (NaN_3_/2DG) is mainly responsible for Cu uptake from CuO NPs, but not from ionic copper
(Moschini et al. 2013)	A549 (Human, adenocarcinoma, alveolar basal epithelia)	CuO NPs, purchased,self-characterised, 34.38 ± 0.76 nm (TEM, sonicated in water, albumin stabilised) 300.04 ± 10.08 nm (DLS, sonicated in water, albumin stabilised)	10 and/or 100 µg/mL 3 and 24 h	BafA1 (50 nM)	Cell viability is significantly higher in cells treated with inhibitor than in control cells, in after 24 h, but not after 3 h (MTT assay) Abundance of intracellular copper ions is lower in cells treated with inhibitor than in control cells after 24 h, but not after 3 h (rhodamine stain, microscopically, not quantified)	Acidification of lysosomes (BafA1) is involved in liberation of copper ions and cytotoxicity Uptake via endo-lysosomal route
(Bulcke et al. 2014)	Primary astrocytes (Rat, astrocytes)	CuO NPs, self-synthesised and characterised, 5 nm (TEM, dispersed in water) 136 ± 4 nm (DLS, in water) 178 ± 12 nm (DLS, in cell culture media with serum)	CuO NPs, Cu(NO_3_)_2_ and CuCl_2_: 100, 300 and 1000 µM Cu 3 h	4 °C	CuO NPs, Cu(NO_3_)_2_ and CuCl_2_: Intracellular copper is significantly lower in cells treated at 4 °C compared to control cells, dose-dependency (GF-AAS) CuO NPs, Cu(NO_3_)_2_ and CuCl_2_: Cell viability is significantly higher in cells treated at 4 °C compared to control cells, dose-dependency (MTT assay) Intracellular copper is significantly higher in cells treated with CuO NPs than in cells treated with ionic copper (GF-AAS)	Active uptake is involved in Cu uptake from all three tested forms of copper and cytotoxicity Uptake differs between CuO NPs and ionic copper
(Chen et al. 2015)	Caco-2 (Human, colorectal adenocarcinoma)	CuO NPs, purchased, self-characterised, 10–20 nm (TEM)	62.5 µM 1 h	CPZ (10 µg/mL) β-CD (10 µg/mL) EIPA (2.5 mM) CD (10 mM) NaN_3_ (10 mM) with 2DG (50 mM) 4°C	Intracellular copper is significantly lower in cells treated with inhibitors or at 4 °C than in control cells (ICP-MS) 4 °C and NaN_3_/2DG: 60–90% lower; CD and β-CD: 40–50% lower; CPZ & EIPA: 20% lower	Active uptake (4°C, NaN_3_/2DG) is mainly responsible for Cu uptake from CuO NPs Probably macropinocytosis and phagocytosis (CD) as well as lipid raft/caveolae (β-CD) are involved Macropinocytosis (EIPA) and clathrin-mediated-endocytosis (CPZ) are only slightly involved
(Song et al. 2015)	C918 (Human, uveal melanoma)	Cu_2_O NPs, self-synthesised and characterised, 45.4 ± 6.9 nm (TEM, as-synthesised) 34.1 ± 9.0 nm (TEM, in cell culture media)	10 µg/mL 3 h	CPZ (25 µM) β-CD (5 mM) EIPA (100 µM) Filipin (4 µg/mL)	Intracellular copper is significantly lower in cells treated with the inhibitors CPZ, β-CD and Filipin than in control cells (ICP-MS) Intracellular copper in cells treated with the inhibitor EIPA is equal to control cells (ICP-MS) β-CD: 60% lower, Filipin: 50% lower, CPZ: 20% lower	Active uptake probably responsible for Cu uptake from Cu_2_O NPs Lipid raft/caveolae (β-CD and Filipin) is involved Clathrin-mediated-endocytosis (CPZ) is only slightly involved Macropinocytosis (EIPA) is not involved
(Yu et al. 2017)	A375 (Human, melanoma)	Cu_2_O NPs, self-synthesised and characterised, 40–110 nm (TEM, in water)	3.5 μg/mL 48 h (CCK8) 3,6,9 h (GF-AAS)	CPZ (0.6–5 µM or 5 µM) β-CD (1.2–10 µM) EIPA (25–100 µM) Dynasore (40–160 µM)	Cell viability is significantly higher in cells treated with the inhibitor CPZ than in control cells (CCK-8 assay) Cell viability in cells treated with the inhibitors β-CD, EIPA or dynasore is equal to control cells (CCK-8 assay) Intracellular copper is significantly lower in cells treated with the inhibitor CPZ in all time points (GF-AAS)	Clathrin-mediated-endocytosis (CPZ) is mainly responsible Lipid raft/caveolae (β-CD), macropinocytosis (EIPA) and dynamin-dependent pathways (dynasore) are not involved
(Joshi et al. 2019)	C6 (Rat, glioma)	CuO NPs self-synthesised and characterised, 191 ± 33 nm (DLS, in albumin containing buffer)	CuO NPs and CuCl_2_: 500 µM 1 h	4°C Cu chelator BCS (100 µM)	CuO NPs and CuCl_2_: Intracellular copper is significantly lower in cells treated at 4°C compared to control cells (GF-AAS) CuO NPs: Intracellular copper in cells treated with Cu chelator BCS is equal to control cells at 37°C (GF-AAS) CuO NPs: Intracellular copper is significantly lower in cells treated at 4°C compared to control cells in the presence of Cu chelator BCS (GF-AAS) CuCl_2_: Intracellular copper is 50% lower in cells treated with Cu chelator compared to control cells (GF-AAS) CuO NPs and CuCl_2_: Cell viability is significantly higher in cells treated at 4°C than in control cells (LDH assay)	Active uptake is involved in Cu uptake from CuO NPs and ionic copper and in cytotoxicity Active uptake of copper from CuO NPs is independent of copper ion release
(Strauch et al. 2020)	BEAS-2B (Human, bronchial epithelium)	CuO NPs purchased, self-characterised, 20—200 nm (TEM) CuO MPs purchased,self-characterised, 0.5—10 µm (TEM)	CuO NPs: 10 µg/mL CuO MPs: 50 µg/mL CuCl2: 630 µM (= 50 µg/mL CuO) 24 h (cell number) 8 h and 24 h (GF-AAS)	BafA1 (100 nM) Dynasore (100 µM)	CuO NPs and CuO MPs: Cell viability is significantly higher in cells treated with the inhibitor BafA1 than in control cells (cell number) CuCl_2_: Cell viability in cells treated with the inhibitor BafA1 is equal to control cells (cell number) CuO NPs: Intracellular copper is significantly lower in cells treated with inhibitors dynasore or BafA1 compared to control cells (GF-AAS) CuO MPs: Intracellular copper is significantly lower in cells treated with the inhibitor dynasore and not affected with treated with the inhibitor BafA1 compared to control cells (GF-AAS) CuCl_2_: Intracellular copper in cells treated with inhibitors dynasore or BafA1 is equal to control cells (GF-AAS) CuO NPs: dynasore: 50% lower, BafA1: 40% lower; CuO MPs: dynasore: 80% lower	Acidification of lysosomes (BafA1) is involved in cytotoxicity from CuO NPs and CuO MPs, but not from CuCl_2_ Uptake via endo-lysosomal route of CuO NPs Dynamin-dependent pathways (dynasore) are involved in CuO NPs and CuO MPs uptake, but not CuCl_2_
(Joshi et al. 2020)	C6 (Rat, glioma)	CuO NPs self-synthesised and characterised, 235 ± 35 nm in albumin containing buffer (DLS)	1 mM 0.5 h	BafA1 (100 nM)	CuO NPs: Cell viability is significantly higher in cells treated with the inhibitor BafA1 than in control cells (LDH activity) CuCl_2_: Cell viability in cells treated with the inhibitor BafA1 is equal to control cells (LDH activity)	Acidification of lysosomes (BafA1) is involved in cytotoxicity from CuO NPs but not from CuCl_2_ Uptake via endo-lysosomal route of CuO NPs
(Geppert et al. 2021)	RTgutGC (Fish, intestinal)	CuO NPs purchased, self-characterised, 188 ± 16 nm in culture media (DLS)	10 mg/L 4 and 24 h Transwell system	β-CD (10 mM) Nystatin (30 µM) Genistein (100 µM) PAO (20 µM) EIPA (20 µM) 4 °C	Apical and basolateral copper content in cells treated with any inhibitor is equal to control cells (ICP-MS) Basolateral copper content is significantly lower in cells treated at 4 °C compared to control cells (ICP-MS) Intracellular copper content is significantly lower in cells treated with the inhibitor genistein and at 4 °C compared to control cells after 4 h but not 24 h (ICP-MS) Genistein: 33% lower, 4 °C: 25% lower	Active processes (4 °C) are partially responsible for intracellular Cu uptake and basolateral transport Lipid raft/caveolae (genistein) is involved Lipid raft/caveolae (β-CD, nystatin), macropinocytosis (EIPA) and clathrin-mediated-endocytosis (PAO) are not involved
(Moschini et al. 2023)	A549 (Human, adenocarcinoma, alveolar basal epithelia)	CuO NPs 33.3 ± 10.7 nm and 24.0 ± 3.5 nm dispersed in isopropylic alcohol (TEM)	25 and 50 µg/mL 3 h	CD (4 µM) BafA1 (50 nM)	Cell viability is significantly higher in cells treated with the inhibitor CD or BafA1 than in control cells (MTT assay)	Acidification of lysosomes (BafA1) is involved in cytotoxicity from CuO NPs Uptake via endo-lysosomal route of CuO NPs Macropinocytosis and phagocytosis (CD) are involved in CuO NPs uptake
(Zhao et al. 2023)	HaCaT (Human, keratinocyte)	CuO NPs purchased,manufacturer information only, < 50 nm	10 mg/L 10 h	NaN_3_ (10 mM) with 2DG (50 mM)	Abundance of a proposed biomarker for metal NPs toxicity, p-H3S10 protein, is lower in cells treated with the inhibitor than in control cells (WB, not quantified)	Active uptake (NaN_3_/2DG) is involved in toxicity from CuO NPs

2DG: 2-Deoxy-D-glucose; BafA1: bafilomycin A1; β-CD: β-cyclodextrin; CCK-8: cell counting kit 8; CD: cytochalasin D; CPZ: chlorpromazine; DLS: dynamic light scattering; EIPA: ethyl-isopropyl amiloride; GF-AAS: graphite furnace atomic absorption spectroscopy; ICP-MS: inductively coupled plasma mass spectrometry; LDH: lactate dehydrogenase; MTT: 3-(4,5-dimethylthiazol-2-yl)-2,5-diphenyltetrazolium bromide; PAO: phenylarsine oxide; TEM: transmission electron microscopy; WB: Western blot

Across the reviewed articles, active uptake of CuO NPs was clearly demonstrated by experiments conducted at 4 °C or presence of metabolic inhibitors such as NaN_3_ and 2-Deoxy-D-glucose (2DG). In these experiments, blocking energy-dependent processes led to a marked decrease in intracellular copper or rescued the cells from toxicity originated from CuO NPs, indicating that internalisation is primarily mediated by active, energy-dependent pathways. For ionic copper, two studies reported evidence of active uptake, whereas another one did not. The evidence for active uptake of CuO NPs is unlikely to represent a false positive outcome, as one study demonstrated that the observed copper uptake occurred independent of dissolved copper ions (Table 2).

The involvement of the endo-lysosomal pathway emerges as another consistent finding across the reviewed literature. In all studies applying bafilomycin A1 (BafA1), an inhibitor of lysosomal acidification and subsequent dissolution of CuO or Cu_2_O NPs, treatment resulted in a clear rescue of cell viability. Moreover, most studies provided evidence supporting the contribution of clathrin-mediated endocytosis. Three studies reported significant reductions in intracellular copper following NPs incubation with parallel inhibition with chlorpromazine (CPZ), whereas one study using phenylarsine oxide (PAO) observed no effect. Collectively these findings suggest that clathrin-mediated pathways play an important, although possibly cell-type-specific, role in CuO NP internalisation (Table 2).

Inhibitors of macropinocytosis and phagocytosis, including cytochalasin D (CD) and ethyl-isopropyl amiloride (EIPA) were applied, in two and four studies, respectively. In both studies using CD, inhibition resulted in reduced uptake, indicated by reduced intracellular copper or rescued cell viability. In contrast three of the four studies employing EIPA reported no effect, and only minor effects were observed in one study (Table 2). Closer examination of the experimental conditions further questions the contribution of macropinocytosis. Notably, one study using CD employed A549 cell line, which exhibited elevated basal micropinocytosis activity, and may therefore overestimate the relevance of this pathway. (Rennick et al. 2021) Overall, the involvement of macropinocytosis or phagocytosis in CuO NP internalisation remains uncertain.

Studies examining dynamin-dependent mechanisms, using dynasore, yielded inconsistent findings: one article reported reduced intracellular copper levels following inhibition, whereas the other observed no rescuing effect on cell viability. Similarly, evidence for lipid raft—or caveolae-dependent internalisation was variable. These pathways were evaluated together because the inhibitors applied, filipin, genistein, β-cyclodextrin (β-CD), and nystatin, affect both pathways. (Rennick et al. 2021) Four studies reported reduced uptake or rescued cell viability upon inhibition, while three found no significant effects (Table 2). Notably, both filipin and nystatin possess well-documented off-target effects through disruption of cholesterol content, which can affect multiple membrane-associated processes, therefore limit the interpretability of findings derived from their use. (Rennick et al. 2021; Smart and Anderson 2002).

The analysis of available literature provides strong evidence that active uptake is a central mechanism in the internalisation of CuO NPs, supported by the consistent decline in intracellular copper upon inhibition of energy-dependent processes. The endo-lysosomal pathway appears particularly important, as bafilomycin A1 treatment consistently mitigated nanoparticle-induced toxicity. The involvement of clathrin-mediated endocytosis also seems plausible based on the available evidence. In contrast, the roles of other specific endocytosis pathways, such as macropinocytosis or phagocytosis, dynamin-dependent or lipid raft/caveolae dependent mechanisms remain uncertain due to inconsistent results and the known off-target effects of various inhibitors used to probe these processes. The overall variability across studies, including differences in cell models, NP characteristics, exposure conditions, and analytical approaches, underscores the need for more standardised experimental designs and further investigations to clarify the uptake mechanisms of CuO and Cu_2_O NPs.

Next, we analysed the selected literature with regards, whether intact CuO and Cu_2_O NPs can be detected within cells and which analytical approaches have been used to demonstrate their presence. Among all studies employing in vitro methods, 13% (64/490) used microscopic techniques, such as scanning transmission electron microscopy (STEM) or TEM, STEM/TEM–EDX, dark-field microscopy, or the reflection-mode laser scanning microscopy to visualise intracellular particles (Fig. 9 C). In many cases, only a single TEM image was provided. However, TEM alone cannot confirm elemental composition of the particle, which is essential to avoid false positive results arising from other electron-dense structures that are no Cu-based NPs. Flow cytometry side-scatter analysis, which offers more quantitative estimate of particle uptake but likewise lacks elemental specificity, was applied in only 2% (9/490) of the in vitro studies. Despite, these methodological limitations, the available evidence indicates that CuO and Cu_2_O NPs are internalised by cells and at least partly present intracellularly as an intact particulate form.

We identified seven articles investigating barrier crossing in vitro. The majority of barrier crossing experiments were performed in intestinal cell models. Four of these employed the Caco-2 cell line, used either as a monoculture (Li et al. 2020; Chen et al. 2015; Ude et al. 2017) or in a co-culture system (Ude et al. 2019). Caco-2 cells are considered an appropriate model for assessing intestinal barrier permeability (Yee 1997). In three of these studies, ionic copper controls were included, and the findings suggest that CuO NPs result to more copper transport across the barrier than ionic copper. One study used fish intestinal cells (Geppert et al. 2021), although no ionic control was included. The other two studies applied co-culture on-chip models representing the placenta or the endothelial barrier. (Cao et al. 2024; Lu et al. 2021) In six of the seven studies, only total copper was measured on the basolateral side, preventing conclusions regarding, whether intact particles crossed the barrier. Evidence for particulate transport was provided only in the endothelial barrier model, in which TEM images indicate NP internalisation by endothelial cells and subsequent presence in cardiac tissue. (Lu et al. 2021).

Because transport across the intestinal barrier was of particular interest for this review, we also analysed in vivo studies in which CuO or Cu_2_O NPs were administered orally. Out of the 62 identified articles, 15 quantified total copper in different tissues or blood, two used microscopy to detect particles, and one combined both approaches (Fig. 9D). One of the studies using microscopy to detect particle in liver tissue reports the same data set as published elsewhere. (Canli et al. 2019) In the original report, particles were detected in the brain, kidney and liver following a 14-day oral exposure of rats to CuO NPs. (Canli, 2020) In the third article, which assessed the presence of particles in tissues, only sections of the intestinal system, i.e., the duodenum, jejunum, ileum and the colon, were analysed. (Mortensen et al. 2021) With this study design, however, transport across the intestinal barrier into the systemic circulation or other organs cannot be evaluated. Among the 16 studies that quantified total copper in tissues or blood, three included ionic copper controls and one additionally incorporated a bulk material control. (Min et al. 2022; Kadammattil et al. 2018; Lopes et al. 2024) The two studies conducted in mammals demonstrated higher systemic levels of total copper following exposure to CuO or Cu_2_O NPs compared with ionic copper. In sheep, total blood copper concentrations after 15 days of oral Cu_2_O NPs administration were more than twofold higher of as compared to CuSO_4_ exposure. (Min et al. 2022) In mice, total copper levels were consistently higher across all examined organs after three days of oral administration of CuO NPs as compared to CuSO_4_. In intestine and liver, concentrations exceeded a twofold increase; in brain and kidney levels were approximately twofold; and in spleen the levels were less than twofold higher. (Kadammattil et al. 2018) A study performed in birds, reported higher total copper content in the tibia after oral administration of CuO NPs compared with CuSO_4_, whereas CuSO_4_ administration led to higher total copper levels in the liver. Administration of bulk CuO particles did not alter total copper levels in liver nor in tibia. (Lopes et al. 2024) Although these findings do to not prove a transport of intact particles across the intestinal barrier, they indicate NP uptake and/or transport may involve distinct mechanisms that differ between species. Overall, only one study provides evidence for intestinal barrier crossing of intact CuO particles, which is insufficient to support definitive conclusions. Nevertheless, the consistently higher total copper levels in tissues after exposure to CuO and Cu_2_O NPs compared with ionic copper point to a distinct mechanism. Inclusion of ionic copper controls in barrier-crossing experiments remains essential, as de novo formation of NPs from ionic forms during digestion has been demonstrated for other metals. (Juling et al. 2016; Sieg et al. 2017).

### Content analysis of involved cell death mechanisms

**Fig. 10:**
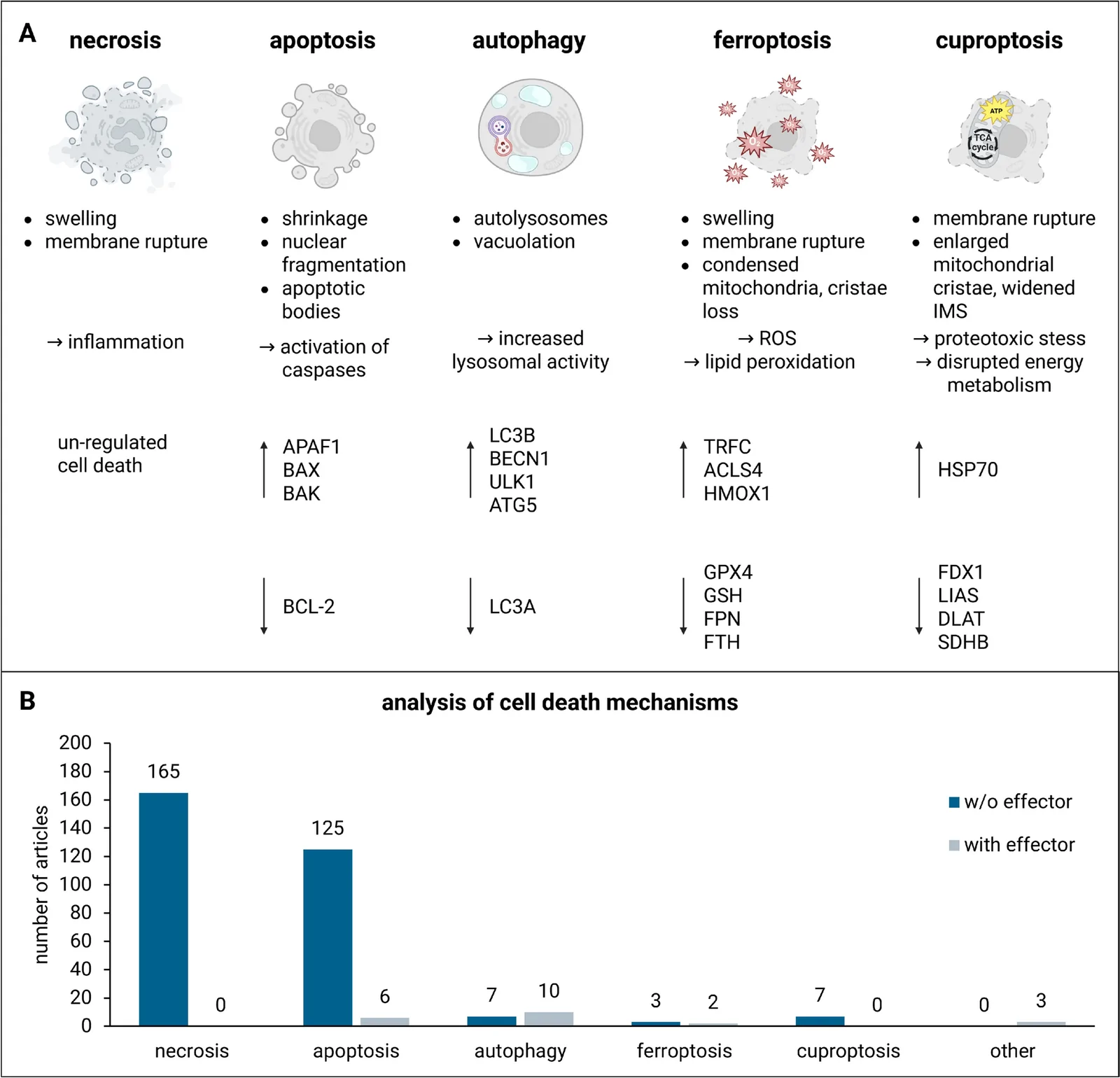
Overview of articles investigating cell death mechanisms induced by CuO or Cu_2_O nanoparticles. **A** Representative morphological characteristics and key molecular regulators of major cell death pathways. (Chen et al. 2023; Saxena et al. 2024; Tsvetkov et al. 2022; Zischka et al. 2011) **B** Number of articles investigating each cell death mechanism, with or without the use of functional effectors such as chemical inhibitors, gene silencing, or knock-out approaches. Individual studies often investigated more than one death pathway. ACLS4: Acyl-CoA synthetase long-chain family member 4; APAF1: apoptotic protease-activating factor 1; ATG5: autophagy related 5; BAK: Bcl-2 homologous antagonist/killer; BAX: Bcl-2-associated X protein; BCL-2: B-cell lymphoma 2; BECN1: beclin 1; DLAT: dihydrolipoamide acetyltransferase; FDX1: ferredoxin 1; FPN: ferroportin; FTH: ferritin heavy chain; GPX4: glutathione peroxidase 4; GSH: glutathione; HMOX1: heme oxygenase 1; HSP70: heat shock protein 70; LC3A/B: microtubule-associated protein 1 light chain 3 alpha/beta; IMS: intermembrane space; LIAS: lipoic acid synthetase; ROS: reactive oxygen species; SDHB: subunit of succinate dehydrogenase (complex II); TRFC: transferrin receptor; ULK1: Unc-51 like autophagy activating kinase 1; w/o: without. Panel A was created in BioRender. Scholz, S. (2026) https://BioRender.com/mvsdq42

The third main key questions to be addressed in this review was to determine whether CuO and Cu₂O NPs exert distinct biological and cellular responses, specifically with regards to cell death mechanisms such as cuproptosis, a recently identified form of cell death (Tsvetkov et al. 2022) To address this, we systematically analysed all in vitro studies that investigated relevant cell death modalities (Fig. 10A). Among the reported mechanisms, apoptosis and necrosis were the most frequently assessed. We further analysed studies that used functional modulators of cell death, such as pathway-specific inhibitors or inducers, or genetic approaches such as gene silencing and genetic knock-outs, because these studies provide strong mechanistic evidence (Fig. 10B). Only six studies used such methods to investigate apoptosis, a remarkably low number, considering the large number of publications examining apoptosis-related endpoints. In contrast, autophagy-related investigations more often included effector-based approaches. For ferroptosis, three studies assessed the pathway without the use of effectors and two with effectors, while cuproptosis was examined in seven studies without pathway modulation, additionally three alternative mechanisms were studied using effector-based methods. Given the evolving terminology and mechanistic understanding of regulated cell death mechanisms over time, it is likely, that some earlier studies analysed relevant endpoints without explicitly defining as being relevant for these specific pathways.

To differentiate the cell death mechanisms by CuO and Cu_2_O NP, we analysed the main findings of studies using functional effectors; for cuproptosis and ferroptosis, we additionally considered studies assessing pathway-associated markers only, due to limited number of articles using such interventions (Tables 3, 4, 5, 6). Across the reviewed literature, six studies investigated apoptosis using the same pan-caspase inhibitor, benzyloxycarbonyl-Val-Ala-Asp(OMe)-fluoromethylketone (zVAD-fmk), at concentrations of 20 to 50 µM (Table 3). The studies employed a variety of cell lines, including A549, HeLa/B16-F10, HUVEC, HCT-116, K562, and RAW 264.7 and in all but one utilised CuO NPs with particle sizes typically below 50 nm, at concentration of 1 to 30 µg/mL over exposure periods of 24–48 h. Only one publication reported that zVAD-fmk treatment restored cell viability, suggesting the involvement of apoptosis. In all other cases, inhibition of apoptosis had no effect on cell viability, and one article was difficult to interpret due to limitations in the study design (Table 3). Overall, these findings suggest that apoptosis is unlikely to represent the primary mechanism of CuO or Cu_2_O NP-induced cell death. The frequent increases in apoptosis-associated markers observed in a large number of studies, lacking inhibitor experiments, likely reflect secondary or downstream effects rather than a causal role in cell death.

**Table 3:** Overview of studies investigating apoptosis induction by CuO and Cu_2_O nanoparticles (NPs) using specific effectors

Study	Cell line	Type of copper oxide nanomaterials	Exposure scenario	Type of effector	Selected results	Interpretation
(Sun et al. 2012)	A549 (Human, adenocarcinoma, alveolar basal epithelia)	CuO NPs, purchased, manufacturer information only, < 50 nm	30 µg/mL 24 h	Inhibitor (zVAD-fmk, 20 µM)	Cell viability in cells treated with inhibitor is equal to control cells (MTS assay)	No involvement of apoptosis
(Wang et al. 2013)	Either HeLa or B16-F10	Cu_2_O NPs, self-synthesised and characterised, 40—110 nm	2.5, 5 and 10 µg/mL 24 and 48 h	Inhibitor (zVAD-fmk, 20µM)	Cell viability is significantly higher in cells treated with inhibitor than in control cells for all tested time-dose combinations except 5 µg/mL after 24 h (MTT assay)	Involvement of apoptosis
(Zhang et al. 2018b)	HUVEC (Human, umbilical vein, endothelial)	CuO NPs, purchased, self-characterised, < 50 nm (TEM) 183.70 nm in culture medium (DLS)	20 µg/mL 24 h	Inhibitor (zVAD-fmk, 50 µM)	Cell viability in cells treated with inhibitor is equal to control cells (MTS assay) Abundance of cleaved PARP and cleaved caspases 3, 6 and 7 proteins in cells treated with inhibitor is equal to cells treated without inhibitor and to medium control cells (WB, not quantified)	No involvement of apoptosis
(Soria et al. 2019)	HCT-116 (Human, colorectal carcinoma)	CuO NPs, purchased, self-characterised < 50 nm (unclear) 220 nm in culture medium (DLS)	2.5 and 5 µg/mL	Inhibitor (zVAD-fmk)	Cell viability in cells treated with inhibitor is equal to control cells (MTT assay)	No involvement of apoptosis
(Tsymbal et al. 2021)	K562 (Human, lymphoblast, myelogenous leukemia)	CuO NPs, self-synthesised and characterised, 80 ± 20 nm (DLS)	1 µg/mL CuO NPs and 1mM NAC 24h	Inhibitor (zVAD-fmk, 50 µM)	Number of apoptotic and necrotic cells is lower in cells treated with inhibitor than in control cells (Flow cytometry, Phycoerythrin and AnnexinV)	Involvement of apoptosis, unclear influence of co-incubated NAC
(Gupta et al. 2022)	RAW.264.7 (Mouse, macrophage)	CuO NPs, purchased, self-characterised, 152 ± 2 nm in culture medium	CuO NPs: 25 and 41 µg/mL CuCl_2_: 55 µg/mL	Inhibitor (zVAD-fmk, 20 µM)	CuO NPs and CuCl_2_: Cell viability in cells treated with inhibitor is equal to control cells (Alamar blue assay)	No involvement of apoptosis (CuO NPs, CuCl_2_)

DLS: dynamic light scattering; MTS: 3-(4,5-dimethylthiazol-2-yl)-5-(3-carboxymethoxyphenyl)-2-(4-sulfophenyl)-2H-tetrazolium; MTT: 3-(4,5-dimethylthiazol-2-yl)-2,5-diphenyltetrazolium bromide; NAC: N-acetylcysteine; TEM: transmission electron microscopy; WB: Western blot; zVAD-fmk: benzyloxycarbonyl-Val-Ala-Asp(OMe)-fluoromethylketone

**Table 4:** Overview of studies investigating autophagy induction by CuO and Cu_2_O nanoparticles (NPs) using specific effectors

Study	Cell line	Type of copper oxide nanomaterials	Exposure scenario	Type of effector	Selected results	Interpretation
(Sun et al. 2012)	A549 (Human, adenocarcinoma, alveolar basal epithelia)	CuO NPs, purchased,manufacturer information only, < 50 nm	30 µg/mL 24 h (Wortmannin, 3-MA) 18 h (BafA1)	Inhibitors (BafA1, 50 nM Wortmannin, 1 µM, 3-MA, 3 mM)	Cell viability is significantly higher in cells treated with inhibitor than in control cells (MTS assay)	Autophagy involved in cell death
			30 µg/mL 24 h	Knock-down (ATG5)	Cell viability in KD is significantly higher than in control cells (MTS assay)	Autophagy involved in cell death
(Laha et al. 2014)	MCF7 (Human, breast cancer)	CuO NPs, self-synthesised and characterised, 30 ± 3.5 nm	0–30 µg/mL 24 h	Inhibitor (3-MA, 5 mM)	Cell viability is significantly lower in cells treated with inhibitor than in control (MTT assay)	Autophagy has a protective role
			Unclear	Knock-down (BECN1)	Abundance of apoptotic protein p-Bad is lower, and of cleaved-caspase 3 higher, than in control cells (WB, quantified)	Autophagy has a protective role
(Song et al. 2015)	C918 (Human, uveal melanoma)	Cu_2_O NPs, self-synthesised and characterised, 45.4 ± 6.9 nm (TEM, as-synthesised) 34.1 ± 9.0 nm (TEM, in cell culture media)	10 µg/mL 3 h	Inhibitor (3-MA, 2 mM)	Abundance of autophagy related proteins ATG5 and beclin 1 is lower, and of LC3A and LC3B is higher, in cells treated with inhibitor than in control cells (WB, not quantified)	Inconsistent results / data not helpful
(Zhang et al. 2018a)	HUVEC (Human, umbilical vein, endothelial)	CuO NPs, purchased, self-characterised, < 50 nm	10 and/or 20 µg/mL 12 or 24 h	Knock-out (*ATG5*)	Cell viability is significantly lower in KO than in WT control (MTS, Calcein acetoxymethyl ester assays)	Autophagy has a protective role
(Zhang et al. 2018b)	HUVEC (Human, umbilical vein, endothelial)	CuO NPs, purchased, self-characterised, < 50 nm	20 µg/mL 24 h	Inhibitor (BafA1, 100 nM)	Cell viability is significantly higher in cells treated with inhibitor than in control (MTS assay)	Autophagy involved in cell death * Inhibitor not specific, acidification of lysosomes
			Unclear	Inhibitor (3-MA and Wortmannin)	Cell viability in cells treated with inhibitor is equal to control cells (MTS assay)	Autophagy not causative for cell death
			Unclear	Knock-down (BECN1)	Cell viability in KD is equal to control cells (MTS assay)	Aautophagy not causative for cell death
(Jiang et al. 2019)	MCF7(Human, breast cancer)	CuO NPs,self-synthesised and characterised, 5.4 ± 1.2 nm	10 µg/mL	Inhibitor (3-MA, 3 mM)	Colony formation in cells treated with inhibitor is higher than in control cells (colony forming assay)	Autophagy involved in cell death
(Du et al. 2022)	SH-SY5Y(Human, neuroblastoma)	CuO NPs, purchased,Self-characterised, 20– 70 nm	WB: 50 µg/ml MTT: 25, 50, 100 µg/mL	Inhibitor (BafA1, 300 nM)	Cell viability is significantly higher in cells treated with inhibitor than in control cells in all concentrations (MTT assay)	Autophagy involved in cell death * Inhibitor not specific, acidification of lysosomes
			WB: 50 µg/ml MTT: 25, 50, 100 µg/mL	Inhibitor (3-MA, 5 mM)	Cell viability is significantly higher in cells treated with inhibitor than in control cells in the highest concentration (MTT assay)	Autophagy involved in cell death
(Gupta et al. 2022)	RAW.264.7 (Mouse, macrophage)	CuO NPs, purchased, self-characterised,152 ± 2 nm in culture medium	25 and 41 µg/mL	Inhibitor (BafA1, 10 nM)	Cell viability is significantly higher in cells treated with inhibitor than in control cells (Alamar blue assay)	Autophagy involved in cell death * Inhibitor not specific, acidification of lysosomes
			CuO NPs: 25 and 41 µg/mL CuCl_2_: 65 and 100 µg/mL	Inhibitor (Wortmannin, 1 µM)	Cell viability in cells treated with inhibitor is equal to control cells (Alamar blue assay)	Autophagy not causative for cell death
(Li et al. 2022)	HUVEC (Human, umbilical vein, endothelial)	CuO NPs, purchased,no information	20 µg/mL 12 h	Inhibitors (3-MA, 5 mM Wortmannin, 2.5 µM CQ, 10 µM)	Gene expression of *HMOX1* is significantly lower in in cell treated with inhibitors than in control cells (qPCR) Abundance of protein HMOX1 in cells treated with inhibitors CQ and 3-MA is significantly lower than in control cells (WB, quantified)	Autophagy leads to oxidative stress response
			20 µg/mL 6 and 9 h	Knock-out (*ATG5*)	Gene expression of *HMOX1* is significantly lower in KO cells than in control cells after 9 h (qPCR) Abundance of protein HMOX1 is significantly lower in KO cells than in control cells after 9 h (WB, quantified)	Autophagy leads to oxidative stress response
(Cao et al. 2024)	hTSCs-derived placental model (Human, trophoblast)	CuO NPs, purchased, self-characterised, 38.69 ± 7.30 nm	20 µg/mL 48 h	Inhibitor (3-MA, 5 mM)	Number of Annexin V and Propidium iodine positive cells is equal in cells treated with inhibitor and in control cells (microscopically, not quantified)	Autophagy not causative for cell death
				Inhibitor (BafA1, 20 nM)	Number of AnnexinV and Propidium iodine positive is higher in cells treated with inhibitor than in control cells (microscopically, not quantified)	Autophagy has a protective role

3-MA: 3-methyladenine; ATG5: autophagy related 5; BafA1: bafilomycin A1; BECN1: beclin 1; CQ: chloroquine; HMOX1: heme oxygenase 1; KD: knock-down; KO: knock-out; LC3A/B: microtubule-associated protein 1 light chain 3 alpha/beta; MTS: 3-(4,5-dimethylthiazol-2-yl)-5-(3-carboxymethoxyphenyl)-2-(4-sulfophenyl)-2H-tetrazolium; MTT: 3-(4,5-dimethylthiazol-2-yl)-2,5-diphenyltetrazolium bromide; p-BAD: phosphorylated Bcl-2-associated death promoter; TEM: transmission electron microscopy; WB: Western blot; WT: wild type

**Table 5:** Overview of studies investigating ferroptosis induction by CuO and Cu_2_O nanoparticles (NPs) with and without using specific effectors

Study	Cell line	Type of copper oxide nanomaterials	Exposure scenario	Type of effector	Selected results	Interpretation
(Gupta et al. 2022)	RAW.264.7 (Mouse, macrophage)	CuO NPs, purchased, self-characterised, 152 ± 2 nm in culture medium	CuO NPs: 25 and 41 µg/mL CuCl_2_: 55 µg/mL	Inhibitor (Fer-1, 10 µM)	CuO NPs: Cell viability in cells treated with inhibitor is equal to control cells (Alamar blue assay) CuCl_2_: Cell viability in cells treated with inhibitor is lower than in control cells (Alamar blue assay)	Ferroptosis not involved in cell death (CuO NPs) Ferroptosis has a protective role (CuCl_2_)
			CuO NPs: 41 µg/mL CuCl_2_: 65 µg/mL	/	Abundance of GPX4 is lower than in medium control cells, but equal to CuCl_2_ (WB, not quantified)	Involvement of ferroptosis, but no nano specific effect
(Chen et al. 2022)	4T1 (Mouse, breast cancer)	CuO_2_-polyvinylpyrrolidone-stabilised, self-synthesised and characterised, 186.9 nm in PBS (DLS)	50 ppm 24 h	/	Abundance of GPX4 protein is significantly lower in cells treated with CuO_2_-PVP NPs than in medium control cells (WB, quantified)	Involvement of ferroptosis
(Su et al. 2023)	HTR-8/SVneo (Human, trophoblast)	CuO NPs, purchased, self-characterised,138.9 ± 2.85 nm in PBS	200 µg/mL 4, 8, 12, 24 h	/	Gene expression of *GPX4* is lower in cells treated with CuO NPs than in medium control cells 8 h after treatment, time-dependency (qPCR) Abundance of GPX4 protein is slightly lower in cells treated with CuO NPs than in medium control cells, time-dependency (WB, not quantified) Abundance of oxidative stress markers HMOX1 and ACLS4 is higher in cells treated with CuO NPs than in medium control cells 4 h after treatment, time-dependency (qPCR and WB, not quantified)	Involvement of oxidative stress 4h after treatmentInvolvement of ferroptosis 8 h after treatment
(Jiang et al. 2024)	RAW.264.7 (Mouse, macrophage)	CuO NPs, purchased, self-characterised, < 200 nm	20 µg/mL 12 h	Inducer (Erastin, 10 µM)	Cell viability is significantly lower in cells treated with inducer than in cells treated with CuO NPs and slightly lower than in cells treated with inducer only (CCK-8) Gene expression of *GPX4* is significantly lower in cells treated with inducer than in cells treated with CuO NPs and lower than in cells treated with inducer only (qPCR)	Induction of ferroptosis leads to less cell viability and less GPX4 expression Ferroptosis is inducible in CuO NPs treated cells
			5, 10, 20 µg/mL 12 h	/	Abundance of GPX4 protein is significantly lower in cells treated with CuO NPs than in medium control cells, dose-dependency (WB, quantified) Gene expression of *FPN* and *FTH* is significantly higher, and of *GPX4* significantly lower in cells treated with CuO NPs than in medium control cells, dose-dependency (qPCR)	Inconsistent results / trend towards involvement of ferroptosis
			20 µg/mL 12 h	/	Effects of CuO NPs on gene expression of *FTH* and *GPX4* are significantly reversed with copper chelator (TTM, 100 µM) (qPCR) Effects of CuO NPs on protein abundance of GPX4 are significantly reversed with copper chelator (TTM, 100 µM) (WB, quantified)	Effects at least partly dependent of free copper ions
(Lin et al. 2024)	CT26	Cu_2_O NPs, self-synthesised and characterised size unclear	Concentration unclear 24 h	/	Activity of GPX4 is lower in cells treated with Cu_2_O than in PBS control cells (GPX4 activity assay kit) Concentration of GSH is lower in cells treated with Cu_2_O than in PBS control cells (GSH assay kit) Lipid peroxidation is higher in cells treated with Cu_2_O than in PBS control cells (microscopically, not quantified)	Involvement of ferroptosis

ACLS4: Acyl-CoA synthetase long-chain family member 4; CCK-8: cell counting kit 8; DLS: dynamic light scattering; Fer-1: ferrostatin-1; FPN: ferroportin; FTH: ferritin heavy chain; GPX4: glutathione peroxidase 4; GSH: glutathione; HMOX1: heme oxygenase 1; PBS: phosphate buffered saline; TTM: tetrathiomolybdate; WB: Western blot

**Table 6:** Overview of studies investigating cuproptosis induction by CuO and Cu_2_O nanoparticles (NPs) without using specific effectors

Study	Cell line	Type of copper oxide nanomaterials	Exposure scenario	Selected results	Interpretation
(Su et al. 2023)	HTR-8/SVneo (Human, trophoblast)	CuO NPs, purchased, self-characterised, 138.9 ± 2.85 nm in PBS	200 µg/mL 4, 8, 12, 24 h	Abundance of DLAT protein oligomers is higher in cells treated with CuO NPs than in medium control cells from 4h after treatment (WB, not quantified) Gene expression of *DLAT* is higher in cells treated with CuO NPs than in medium control cells, time-dependency (qPCR) Abundance of FDX1 protein is slightly lower in cells treated with CuO NPs than in medium control cells (WB, not quantified) Gene expression of *FDX1* peaks 4 h after treatment with CuO NPs compared to medium control cells (qPCR)	Involvement of cuproptosis 4h after treatment
(Jiang et al. 2024)	RAW.264.7 (Mouse, macrophage)	CuO NPs, purchased, self-characterised, < 200 nm	5, 10, 20 µg/mL 12 h	Abundance of DLAT protein oligomers is higher in cells treated with CuO NPs than in medium control cells, dose-dependency (WB, not quantified) Gene expression of *DLAT*, *LIAS*, *FDX1* and *HSP70* is significantly higher in cells treated with CuO NPs than in medium control cells, dose-dependency (qPCR) Concentration of pyruvate is significantly higher in cells treated with CuO NPs than in medium control cells, dose-dependency (colorimetric assay)	Involvement of cuproptosis
(Li et al. 2024a)	Lenvatinib resistant Huh7 or Hep 3B (Human, hepatocellular carcinoma)	CuO, NPs, self-synthesised and characterised, 150 nm	0.8 µM (0.0636 µg/mL)	Abundance of DLAT, LIAS and FDX1 protein in cells treated with CuO NPs is equal to medium control cells (WB, quantified)	No involvement of cuproptosis
(Lu et al. 2024)	B16 (Mouse, melanoma)	CuO NPs, purchased, self-characterised, 104 nm	CuO NPs: 0.4 µg/mL (5µM) CuCl_2_: 5 µM (0.6722 µg/mL) 2h	Abundance of DLAT protein oligomers in cells treated with CuO NPs or CuCl_2_ is equal to medium control cells (immunocytochemistry, not quantified) Abundance of FDX1 protein is slightly lower in cells treated with CuO NPs and CuCl_2_ than in medium control cells (WB, quantified)	Inconsistent results / only slight effects (CuO NPs and CuCl_2_)
(Luo et al. 2024)	mESC (Mouse, embryonic stem cells)	CuO NPs, purchased, self-characterised,274.2 ± 7.6 nm in culture medium	unclear	Concentration of α-KG is significantly lower in cells treated with CuO NPs than in medium control cells	Involvement of cuproptosis
(Wu et al. 2024)	4T1 (Mouse, breast cancer)	CuO NPs, self-synthesised and characterised, 31.15 ± 0.07 nm	unclear, likely below 2 µg/mL 24 h	Abundance of DLAT protein oligomers in cells treated with CuO NPs is equal to medium control cells (WB, not quantified)	No involvement of cuproptosis
(Zhang et al. 2024a)	HUVEC (Human, umbilical vein, endothelial)	CuO NPs, purchased, self-characterised, 11–27 nm	12 and 24 µg/mL	Abundance of DLAT, FDX1, SDHB protein is significantly lower in cells treated with CuO NPs than in medium control cells, dose-dependency (WB, quantified) Concentration of pyruvate is significantly higher in cells treated with CuO NPs than in medium control cells (colorimetric assay)	Involvement of cuproptosis

α-KG: alpha-ketoglutarate; DLAT: dihydrolipoamide acetyltransferase; FDX1: ferredoxin 1; HSP70: heat shock protein 70; LIAS: lipoic acid synthetase; PBS: phosphate buffered saline; SDHB: subunit of succinate dehydrogenase (complex II); WB: Western blot

We identified ten studies investigating autophagy, seven of which employed multiple inhibitors or combined genetic knock-out with chemical inhibitor (Table 4). Interpretating these data is especially challenging, because autophagy can function as a cytoprotective stress-response mechanism and a driver of cell death (reviewed by Das et al. 2012). The analysed data span a range of systems, including A549, MCF7, C918, HUVEC, RAW 264.7, SH-SY5Y, and trophoblast-derived placental models and predominantly concerned CuO NPs. Across studies, test concentration ranged from 10 to 30 µg/mL, and commonly used inhibitors included 3-methyladenine (3-MA), bafilomycin A1 (BafA1), and wortmannin. In addition, some studies used genetic suppression of autophagy via gene silencing or knock-out of autophagy-related proteins such as ATG5 or beclin 1 (BECN1). The overall evidence regarding the role of autophagy in CuO and Cu_2_O NP-induced cell death remains inconsistent. Some studies report a cytoprotective role of autophagy against NP-induced toxicity, whereas others find no measurable effect or described autophagy contributing to cell death (Table 4). Findings, based on BafA1 should be interpreted cautiously, as this compound also affects lysosomal acidification and may inhibit copper ion release from particles, indirectly improving cell viability (Fig. 9). No consistent trends were identified with respect to incubation times, concentration, cell model, or particle sizes that would account for the differing outcomes across studies. Interpretation is further complicated by the fact that cancer cell lines may exhibit altered autophagy behaviour, adding an additional layer of complexity to data evaluation. (Jin et al. 2017).

Furthermore, several studies examined the contribution of ferroptosis—an iron-dependent form of regulated cell death—to the toxicity of CuO and Cu_2_O NPs (Table 5). Across five studies, two mouse derived cell lines, i.e., RAW 264.7 and 4T1, and two human derived cell lines, i.e., HTR-8/SVneo and HUVEC, were exposed to CuO and Cu_2_O NPs with hydrodynamic diameter below 200 nm. The NP concentrations applied were generally below 20 µg/mL, although one study used a substantially higher concentration of 200 µg/mL. Exposure duration ranged from 4 to 24 h. The primary markers evaluated included glutathione peroxidase 4 (GPX4), ferroportin (FPN), ferritin heavy chain (FTH), Acyl-CoA synthetase long-chain family member 4 (ACSL4), and oxidative stress markers such as heme oxygenase 1 (HMOX1), all of which are commonly associated with ferroptosis. Initially, the results appear to support the involvement of ferroptosis in CuO and Cu_2_O NP- induced cell death, given the frequent observation of ferroptosis-consistent alterations, including GPX4 downregulation and TFRC upregulation. However, a more detailed review of the results indicates that these changes likely represent secondary responses of oxidative stress caused by free copper ions. For instance, one study reported increased expression of oxidative stress markers (HMOX1 and ACSL4) after 4 h of exposure, while GPX4 downregulation was not observed until 8 h post treatment, indicating that oxidative stress precedes the downreguation of GPX4. The hypothesis that copper ions underlie the observed effects, is supported by two further studies. One demonstrated that the changes in ferroptosis-associated markers were partly reversed by the copper chelator tetrathiomolybdate (TTM), while another demonstrated that treatment with CuCl_2_ resulted in a comparable reduction of GPX4 protein levels. Moreover, the causal involvement of ferroptosis appears unlikely, as the only study using an inhibitor found not protective effect of ferrostatin-1 (Fer-1) (Table 5).

All studies investigating cuproptosis were published after 2023, following its identification as a distinct cell death mechanism in 2022. (Tsvetkov et al. 2022) These studies were conducted exclusively with CuO NPs and primarily utilised mouse-derived cell lines, RAW 264.7, B16, mESC and 4T1, along with human-derived cell lines, HTR-8/SVneo, Huh7 or Hep 3B and HUVEC (Table 6). Experimental parameters varied widely with NP sizes ranging from 11 to 200 nm, concentrations from 0.0636 to 200 µg/mL, and exposure durations 2 to 24 h. Core indicators of cuproptosis such as DLAT oligomerisation, changes in protein abundance of LIAS, FDX1, or HSP70, as well as alterations in pyruvate, or α-KG levels were affected in the majority of studies, although some reported no significant changes in these markers. Notably, the studies that did not detect changes in cuproptosis markers generally used very low concentrations of copper oxide NPs or examined modified CuO NPs intended for pharmacological applications, with unmodified CuO NPs or CuCl_2_ serving as ineffective controls. Interestingly, several studies reported higher gene expression of the cuproptosis markers DLAT, LIAS and FDX1 (Table 6). However, reduced protein levels of these markers are considered a defining feature of cuproptosis. (Tsvetkov et al. 2022) Given that cuproptosis is a recently characterised mechanism and its regulatory dynamics remain incompletely understood, we have interpreted all studies determining positive or negative effects on gene or protein expression as indicative of involvement in this pathway. Further uncertainty arises from the lack of functional studies using molecular effectors. To date, no inhibitors for cuproptosis have been identified, and no there are also no studies reporting the utilisation of gene knock-out or silencing strategies. Finally, there remain a complete absence of studies employing Cu_2_O NPs in this context.

Taken together, the available evidence suggests that apoptosis is unlikely to play a causal role in CuO or Cu_2_O NPs-induced cell death, and that ferroptosis-associated mechanisms may largely reflect secondary consequences of copper ion-driven oxidative stress rather than primary death mechanism. The contribution of autophagy-related processes remains uncertain, likely because autophagy also serves as a cellular stress response mechanism that can promote cellular survival as well as cell death. Instead, among the examined pathways, cuproptosis appears mechanistically the most plausible one, however, the current interpretation remains constrained by the limited number of studies the absence of rigorously mechanistic investigations. Furthermore, distinguishing between the effects of copper ions and those of CuO or Cu_2_O NPs remains difficult, as only two studies used a soluble copper control for the relevant endpoints, and one of the studies used concentrations too low for meaningful interpretation. By contrast the study by Gupta et al. (2022), used CuCl_2_ and CuO at reasonable concentrations and systematically investigated several cell death mechanisms using specific inhibitors, rendering it a key reference for assessing NP-specific effects. (Gupta et al. 2022) In this study, none of the investigated mechanisms, autophagy, ferroptosis nor apoptosis contributed to cell death induced by either CuO NP nor CuCl_2_. Instead, the authors identified a cell death pathway characterised by protein aggregation, polyubiquitination and reduced proteasome activity. Interestingly, these characteristics are all associated with cuproptosis as reviewed by Wang et al. (2023), and the authors likewise propose cuproptosis as the most likely mechanism. (Wang et al. 2023) However, it remains uncertain whether the observed effects differ quantitatively from those induced by ionic copper. In in the study from Gupta et al. (2022), proteasomal activity was more strongly impaired by CuO NPs than by CuCl_2_, although protein aggregation and polyubiquitination were not evaluated in the CuCl_2_-treated cells. (Gupta et al. 2022) Moreover, this study did not examine key markers of cuproptosis such as FDX1, LIAS, and DLAT nor did it assess metabolites of the TCA cycle.

## Towards the development of an adverse outcome pathway

**Fig. 11:**
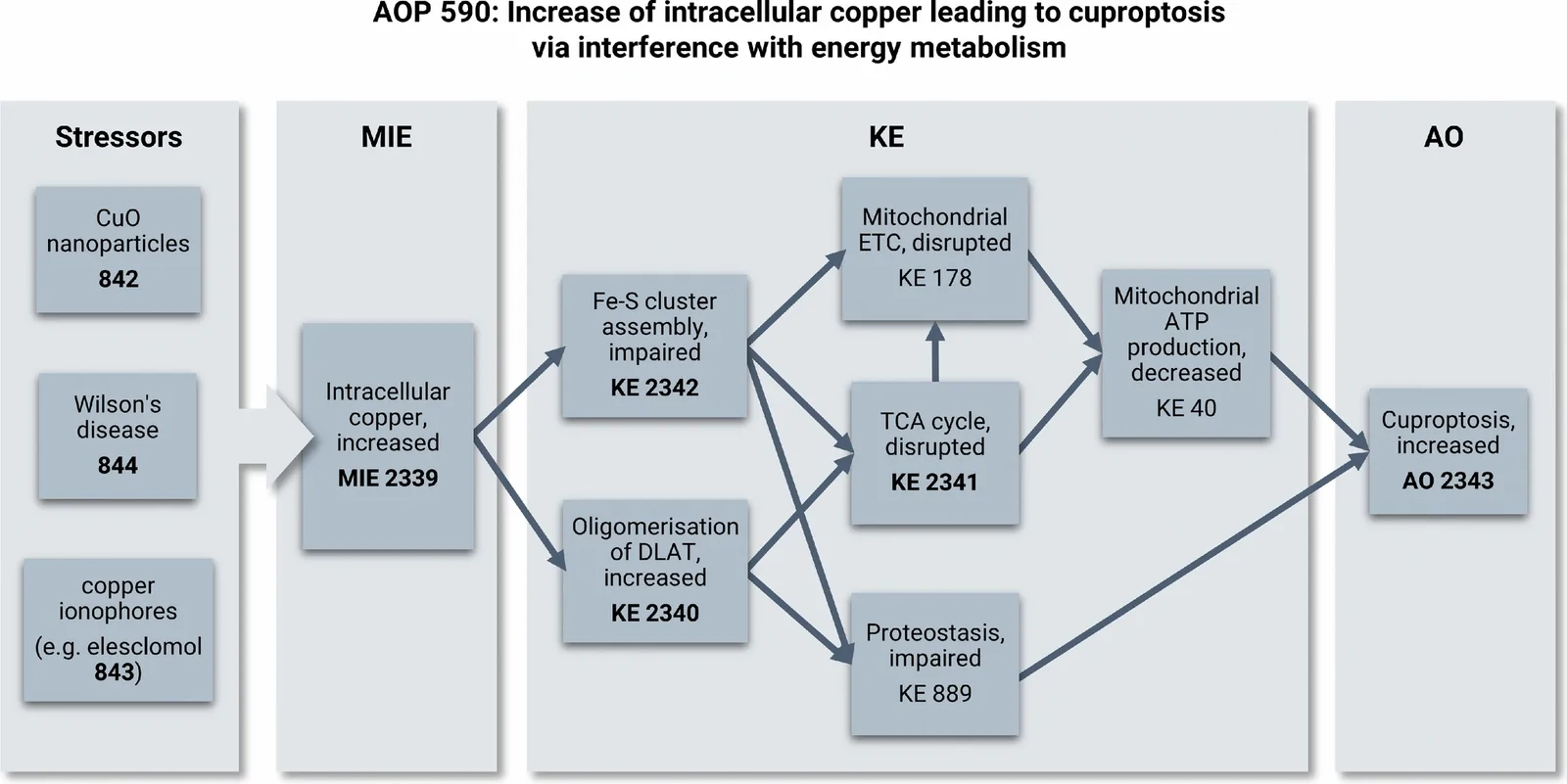
Proposed adverse outcome pathway (AOP) for cuproptosis induction by increased intracellular levels of copper. Various stressors can trigger the molecular initiating event (MIE), followed by a sequence of key events (KE), ultimately resulting in the adverse outcome (AO). Key event relationships (KERs) are indicated by arrows. The IDs assigned to the MIE, KEs and AOP correspond to entries IDs in the AOP Wiki. In bold denote elements newly created in the Wiki as part of this work. Supporting evidence is provided by Bull et al. 1993, Chen et al. 2015, Strauch et al. 2020, Moschini et al. 2013, Tsvetkov et al. 2022, Su et al. 2023 and Yu et al. 2017. ATP: adenosine triphosphate; DLAT: dihydrolipoamide acetyltransferase; ETC: electron transport chain; TCA: tricarboxylic acid

To synthesise the key events linking increased intracellular levels of copper to cuproptosis, we propose an adverse outcome pathway (AOP) based on the literature analysis performed in this work (Fig. 11). For broader accessibility and collaborative development, we uploaded this AOP to the AOP Wiki as AOP 590: *Increase of intracellular copper leading to cuproptosis *via* interference with energy metabolism*. (aopwiki.org 2025) The intention of this AOP is to provide an initial structural framework that will support the identification of related pathways, potential stressors, and mechanistic interactions, thereby advancing the current mechanistic understanding of cuproptosis. This is particularly relevant given that cuproptosis has only recently been defined as an adverse outcome (AO). (Tsvetkov et al. 2022) We classified the proposed AOP as “partially developed” according to the OECD guidance on AOP development, as we expect the identification of additional key events (KE) as research on cuproptosis advances. (OECD 2017) Even partially developed AOPs can already support hazard assessment, consistent with the overarching aim of this review on the evaluation of CuO and Cu₂O NP toxicity.

Even though additional KEs may remain to be identified in the future, the maturity and level of confidence of the proposed AOP can be assessed using the Bradford Hill criteria tailored for AOP assessment. (Hill 1965; Becker et al. 2015; Vinken et al. 2013) The main criteria are the support for key event relationships (KERs) and the essentiality of individual KEs (Supplementary Table 3). Further, we assess the strength and consistency of the association between molecular initiating event (MIE) and AO, and discuss the presence of alternative mechanisms and/or data gaps as suggested by the Handbook for AOP development from the OECD. (OECD 2018).

### Support for KERs

In order to identify the relationships between individual KEs and to identify which events are upstream and which are downstream of the AOP, evidence for dose–response concordance and temporal concordance is evaluated. Due to experimental complexity to assess this on the level of a single KER, the OECD guidance for AOP development recommends the use of prototypical stressors. Those KEs that are affected at lower doses, are expected to be the upstream KEs. For temporality, it is assessed which KE occurs earlier to identify the upstream KE. Further, the overall biological plausibility needs to be considered, e.g., a structural or functional relationship. (OECD 2018).

As prototypical stressors that lead to an increase of intracellular copper, we propose Wilson’s disease (stressor 844) and the copper ionophore elesclomol (stressor 843) (Supplementary Table 3). At the end of this section, we also summarise evidence from our systematic literature search for CuO and Cu_2_O NPs as possible stressors. Both, Wilson’s disease as well as elesclomol trigger the MIE 2339, increased intracellular copper levels and with the correct experimental set-up they also serve for analysis of dose–response relationships. Wilson’s disease models lead depending on severity of the disease to higher intracellular copper levels (MIE 2339) in vivo (Lichtmannegger et al. 2016) and elesclomol (stressor 843) leads dose-dependently to higher intracellular copper levels (MIE 2339) in vitro. (Garza et al. 2022).

Despite the possibility of using Wilson’s disease models for dose–response analysis, we could not identify a suitable study that assessed more than on KE of this AOP. Also, data using elescolomol is scarce and we could only identify a single study. It was demonstrated that both, reduction of Fe-S cluster proteins (KE 2342) and formation of DLAT oligomers (KE 2340) are triggered at lower concentrations than an increase proteotoxicity marker HSP70 (KE 889) is observed. (Tsvetkov et al. 2022) This supports the hypothesis that the effects of Fe-S cluster proteins (KE 2342) and DLAT oligomerisation (KE 2340) lead to impaired proteotoxicity (KE 889) downstream. These KERs are also biologically plausible. Misfolded or aggregated proteins are known to induce proteotoxic stress which can lead to the collapse of the proteostasis network, as reviewed by Hipp and Hartl 2024. 

Similarly, also data with temporality are very limited. One study used elesclomol and assessed the metabolome at several timepoints. In this dataset several TCA cycle metabolites and ATP were analysed and α-KG levels (KE 2341) were already affected at earlier timepoints than ATP levels (KE 40). The study also provides a biologically plausible mechanism via the ETC (KE 178), as no direct effects on ATP production capability were observed, but reduced maximal respiratory capacity. (Tsvetkov et al. 2022) This supports the hypothesis that effects on the TCA cycle (KE 2341) and the ETC (KE 178) are likely upstream of effects on the ATP level (KE 40). For all other KERs we could not identify experimental dose–response or temporality data. However, they are biologically plausible as we have reviewed above in the section about molecular mechanisms of copper toxicity.

### Essentiality of KEs

The essentiality of KEs is most effectively demonstrated through genetic knockout, gene silencing studies or the use of specific inhibitors. Using these approaches, which provide direct evidence, the essentiality of Fe-S cluster assembly (KE 2342) for normal TCA cycle function (KE 2341), ETC (KE 178), and maintenance of cellular ATP levels (KE 40) have been demonstrated both in vitro and in vivo. (Ling et al. 2021; Yang et al. 2025; Smíd et al. 2006) Since Fe–S cluster assembly (KE 2342) is required for the correct folding and maturation of functional proteins, intact Fe–S cluster assembly can be considered essential for proteostasis (KE 889) by definition, thus further assessment is not required. (Leal and Gomes 2010) Direct essentiality of a functional TCA cycle (KE 2341) for the ETC (KE 178), and ATP production (KE 40), as well as the dependence of mitochondrial ATP production (KE 40) on an intact ETC (KE 178), has been confirmed in vitro using knockout, knockdown or inhibition studies. (Park et al. 2016; Ryan et al. 2021; Gobelli et al. 2023).

In contrast, the essentiality of DLAT oligomerisation (KE 2340) for downstream KEs is experimentally more challenging to demonstrate, as it an effect on protein level that cannot be experimentally mimicked by knockout studies. Nevertheless, direct evidence for the essentiality of DLAT oligomer formation for proteotoxic stress during cuproptosis exists. In one study, the inhibitor N-acetylcysteine prevented DLAT oligomer formation (KE 2340) and was associated with significantly reduced expression of the proteotoxic stress marker heat shock protein 70 (HSP70) (KE 889). (Chen et al. 2025) Also, direct evidence exists for the essentiality of DLAT oligomerisation (KE 2340) for cell death via cuproptosis (AO 2343). The study demonstrates that a knockout of *FDX1*, the enzyme involved in normal lipoylation of DLAT, prevents DLAT oligomer formation (KE 2340) and cuproptosis (AO 2343) upon treatment with elesclomol (stressor 843). (Tsvetkov et al. 2022) However, we could not identify a suitable study that demonstrates the essentiality of DLAT oligomerisation (KE 2340) directly for disruption of TCA cycle function (KE 2341).

### Strength and consistency of the association

The strength of the association between increased intracellular copper levels (MIE 2339) and cuproptosis (AO 2343) is supported by multiple rescue experiments demonstrating a direct causal relationship. In a rat Wilson’s disease model, treatment with the novel copper chelator methanobactin resulted in dose-dependent reductions of hepatic and mitochondrial copper (MIE 2339), restoration of mitochondrial morphology and ATP levels (KE 40), and improved survival (AO 2343). (Einer et al. 2023) In an in vitro ATP7B-deficient HepG2 model of Wilson’s disease, chelation with D-penicillamine reduced intracellular copper (MIE 2339) and rescued cell viability (AO 2343). (Chandhok et al. 2014) Similarly, in mouse myoblasts treated with the copper ionophore elesclomol (MIE 2339), chelation with tetrathiomolybdate (TTM) restored ATP content (KE 40), mitochondrial respiratory complex I and III activity (KE 178), and cell viability (AO 2343). (Fu et al. 2024) Rescue of cell viability (AO 2343) by TTM was also observed when intracellular copper levels were increased (MIE 2339) via SLC31A1 overexpression in both HEK and ABC1 cells. (Tsvetkov et al. 2022) Together, these studies demonstrate that reduction of intracellular copper directly prevents downstream KEs and the AO, providing strong evidence for a causal link between the MIE and AO.

The consistency of this association is supported by the observation that comparable downstream effects and rescue responses are observed across multiple experimental systems, including in vitro and in vivo Wilson’s disease models, different cell types, and distinct mechanisms of copper accumulation, indicating a robust and biologically coherent relationship between increased intracellular copper and cuproptosis that is stressor unspecific.

### Alternative mechanisms and data gaps

The available data provide support for dose–response concordance and temporal concordance between some of the KEs in this AOP which are also biologically plausible. This allows to establish upstream–downstream relationships between these KEs. However, the data were very limited and do not allow to establish quantitative thresholds or precise temporal sequences. On the other hand, we could identify several studies demonstrating the essentiality for almost all KEs for one or more of the proposed downstream KEs. Only the direct essentiality of DLAT oligomerisation for TCA cycle disruption is not experimentally demonstrated yet. It was even demonstrated in a knockout study with *DLAT*, that other metabolic pathways can compensate the complete loss of DLAT as the impact on TCA cycle metabolites (KE 2341) is low. (Wang et al. 2025) Thus, it is possible that the observed effects on the TCA cycle may not be partially a result of DLAT oligomerisation, but of impaired Fe–S cluster assembly alone.

### CuO and Cu_2_O NPs as possible stressors

Given that the proposed KEs and KERs are supported by biological plausibility and limited empirical evidence using established copper-related stressors, we next assessed whether particulate copper forms, specifically CuO and Cu₂O NPs, can also act as stressors of this AOP. There was enough experimental data to draw conclusions for CuO NPs, however the evidence for Cu_2_O NPs remains limited due to scarcity of relevant data, although the underlying mechanism appears equally plausible. It was demonstrated that CuO NPs, can be internalised by cells via endocytosis (Chen et al. 2015; Yu et al. 2017), accumulate in lysosomes, and subsequently release copper ions (Moschini et al. 2013; Strauch et al. 2020). These distinct uptake mechanisms likely result in increased copper levels in several tissues as compared to ionic copper exposure, as shown in two mammalian in vivo studies. (Kadammattil et al. 2018; Min et al. 2022) This supports the hypothesis that CuO NPs can trigger the MIE 2339, increased intracellular copper. Additionally, there is evidence that three KEs are triggered. We identified two studies, reporting disruption of the TCA cycle (KE 2341) (Luo et al. 2024; Zhang et al. 2024a), two studies demonstrating DLAT oligomer formation (KE 2340) (Jiang et al. 2024; Su et al. 2023) and one study demonstrating impaired proteostasis (KE 889). (Jiang et al. 2024) Collectively, using the AOP framework and the systematic data analysis performed in this work, we synthesise available evidence and provide initial support that CuO NPs can trigger cuproptosis.

## Conclusions

This review addressed three key questions concerning copper oxide NPs in the food and feed context. First (i), we evaluated whether CuO and Cu₂O NPs are adequately addressed within EU regulations to prevent unintentional oral exposure. This was complemented by an overview of copper and copper oxide exposure, homeostasis, and toxicity, as well as the specific analytical and toxicological challenges related to NP detection. This established a basis for the subsequent sections, in which we then carried out a systematic bibliometric and content analysis of the toxicological literature to assess (ii) whether CuO and Cu_2_O NPs can cross the intestinal barrier as intact particles and accumulate in tissues, and (iii) whether these NPs elicit distinct biological responses, including regulated cell death mechanisms such as cuproptosis. Finally, to integrate the mechanistic evidence and key events linking intracellular copper accumulation to cuproptosis, we propose an AOP based on the findings identified and summarised in this review.

Regarding question (i), the regulatory status of CuO and Cu_2_O NPs within the EU legislation varies widely across the legal domains. Notably, we did not identify any field of law in which CuO and/or Cu_2_O are authorised as conventional materials and in which there are also sufficient legally binding documents to prevent unintentional presence and potential oral exposure of CuO and/or Cu_2_O in nanoforms. For intentionally produced CuO and Cu_2_O NMs, several Regulations already include specific data requirements for applicants. This suggests the possibility of unintended oral exposure to CuO and Cu_2_O NPs via the food and feed chain. However, no data are currently available on the actual presence of NPs or a nanoparticulate fraction in the regulated materials. This absence of empirical information constitutes a clear data gap.

Regarding question (ii) dealing with cellular uptake and translocation across the intestinal barrier, our systematic analysis indicates consistent evidence that CuO and Cu_2_O NPs, are internalised by cells and remain at least partially present as intact particles intracellularly. Uptake appears to involve active processes, with evidence for clathrin-mediated endocytosis, while evidence for additional uptake mechanisms remains inconclusive (Table 2). It should be noted that the use of chemical inhibitors may lead to off-target effects which warrants cautious interpretation of findings (reviewed by Rennick et al. 2021). Transport of intact particles across the intestinal barrier has been examined in only a single study, which is not sufficient to draw definite conclusions. Multiple studies have reported increased total copper content in tissues and cells, as well as an enhanced transport following NP exposure compared with ionic copper controls. (Chen et al. 2015; Kadammattil et al. 2018) These observations suggest that CuO NPs, and potentially also Cu_2_O NPs, may exhibit distinct biological behaviour compared to ionic copper, supporting the need for further targeted investigations. Furthermore, it is important to emphasise that most available data pertain to CuO NPs, with only limited studies addressing Cu₂O NPs.

Regarding question (iii), analysing distinct biological responses and specifically the mechanisms of cell death, our analysis suggests cuproptosis as the most likely causally involved pathway following CuO NP exposure (Table 6). This interpretation aligns with the newly developed AOP 590, which describes increased intracellular copper leading to cuproptosis through disruption of energy metabolism, and several of its key events were also observed following CuO NP treatment (Fig. 11). In contrast, apoptosis does not appear to play a causal role (Table 3), and ferroptosis-related markers may reflect secondary effects driven by oxidative stress induced by ionic copper (Table 5). The role of autophagy-related cell death remains uncertain (Table 4). Notably, these conclusions are limited by insufficient systematic comparison to ionic copper controls, leaving unresolved whether the observed effects differ quantitatively from those elicited by ionic copper exposure. Furthermore, Cu₂O NPs have been hardly investigated in the context of cell death mechanisms, and we did not identify a single article investigating cuproptosis induction by Cu_2_O NPs.

In conclusion, this review shows that current regulatory frameworks only partially address copper oxide NPs, and that critical uncertainties remain regarding their unintended presence in regulated materials and resulting exposure scenarios. The evidence gathered on cellular uptake indicates that CuO and Cu_2_O NPs can be internalised by cells and may behave differently from ionic copper, yet data on intestinal translocation and tissue accumulation remain limited. Furthermore, the available mechanistic findings point to cuproptosis as a key pathway for CuO NP toxicity, while the biological effects of Cu₂O NPs are still largely uncharacterised. Considering this, the proposed AOP provides an initial conceptual structure for organising existing evidence and identifying additional mechanistic links relevant to copper-mediated toxicity. Advancing this framework, along with filling the empirical data gaps on occurrence, uptake, and detailed understanding of toxicity mechanisms, will require concerted analytical, regulatory, and mechanistic research efforts to enable a sound risk assessment of CuO and Cu_2_O NPs in the food and feed context.

## Supplementary Information

Below is the link to the electronic supplementary material.

Supplementary file1 (XLSX 232 KB)

Supplementary file2 (PDF 430 KB)

## Abbreviations

I/II/III/IV: Complex I/II/III/IV of the electron transport chain
2DG: 2-Deoxy-D-glucose
3-MA: 3-Methyladenine
5-hmC: Hydroxymethylation
5-mC: DNA methylation
8-OhdG: 8-Oxo-7,8-dihydro-2′-deoxyguanosine
α-KG: Alpha-ketoglutarate
ACLS4: Acyl-CoA synthetase long-chain family member 4
ACP: Acyl carrier protein
ADI: Acceptable daily intake
ADME: Absorption, distribution, metabolism, and excretion
ANS: EFSA Scientific Panel on Food Additives and Nutrient Sources Added to Food
AO: Adverse outcome
AOEL: Acceptable operator exposure level
AOP: Adverse outcome pathway
APAF1: Apoptotic protease-activating factor 1
APP: Amyloid precursor protein
ATG5: Autophagy related 5
ATOX1: Antioxidant 1 copper chaperone
ATP/ADP: Adenosine triphosphate/adenosine diphosphate
ATP: Adaptation to technical progress
ATP7A/B: ATPase copper transporting alpha/beta
BafA1: Bafilomycin A1
BACE1: β-Site APP-cleaving enzyme 1
β-CD: β-Cyclodextrin
BAK: Bcl-2 homologous antagonist/killer
BAX: Bcl-2-associated X protein
BCL-2: B-cell lymphoma 2
BECN1: Beclin 1
BSA: Bovine serum albumin
bw: Bodyweight
CD: Cytochalasin D
CCK-8: Cell counting kit 8
CCS: Copper chaperone for superoxide dismutase
CCM: Cell culture media
CLP: Classification, labelling and packaging of substances and mixtures
COX: Cytochrome c oxidase
CPZ: Chlorpromazine
CQ: Chloroquine
CtBP1: C-terminal binding protein 1
CTR1: Copper transporter 1
CTR2: Copper transporter 2
Cyt C: Cytochrome c
DCTYB: Cytochrome b reductase 1
DLD: Dihydrolipoamide dehydrogenase
DLS: Dynamic light scattering
DMT1: Divalent metal transporter 1
DLAT: Dihydrolipoamide acetyltransferase
EC: European Commission
EDX: Energy-dispersive X-ray spectroscopy
EFSA: European Food Safety Authority
EIPA: Ethyl-isopropyl amiloride
Endo-III: Endonuclease III
ETC: Electron transport chain
EU: European Union
FADH_2_/FAD: Flavin adenine dinucleotide
FCM: Food contact materials
FEEDAP: EFSA Panel on Additives and Products or Substances used in Animal Feed
Fer-1: Ferrostatin-1
FDX1: Ferredoxin 1
FH: Fumarate hydratase
Fpg: Formamidopyrimidine DNA glycosylase
FPN: Ferroportin
FTH: Ferritin heavy chain
GCSH: Glycine cleavage system H protein
GF-AAS: Graphite furnace atomic absorption spectroscopy
GI: Gastrointestinal
GIT: Gastrointestinal tract
GPX4: Glutathione peroxidase 4
GSH: Glutathione
HBGVs: Health-based guidance values
HMOX1: Heme oxygenase 1
HSP70: Heat shock protein 70
ICP-MS: Inductively coupled plasma mass spectrometry
IMS: Intermembrane space
Isca1: Iron-sulfur cluster assembly 1 homolog
IscS: Cysteine desulfurase
IscU/2: Iron-sulfur cluster assembly scaffold protein/2
KE: Key event
KD: Knock-down
KO: Knock-out
LAMP1: Lysosome-associated membrane protein 1
LC3A/B: Microtubule-associated protein 1 light chain 3 alpha/beta
LDH: Lactate dehydrogenase
LIAS: Lipoic acid synthetase
LIPT1/2: Lipoyltransferase 1/2
MIE: Molecular initiating event
MT: Metallothionein
MTS: 3-(4,5-Dimethylthiazol-2-yl)-5-(3-carboxymethoxyphenyl)-2-(4-sulfophenyl)-2H-tetrazolium
MTT: 3-(4,5-Dimethylthiazol-2-yl)-2,5-diphenyltetrazolium bromide
MUC2: Mucin 2
NAC: N-acetylcysteine
NADH/NAD^+^: Nicotinamide adenine dinucleotide
NAMs: New approach methodologies
NMs: Nanomaterials
NPs: Nanoparticles
NTA: Nanoparticle tracking analysis
PAO: Phenylarsine oxide
p-BAD: Phosphorylated Bcl-2-associated death promoter
PBS: Phosphate buffered saline
PDH: Pyruvate dehydrogenase
PINK1: PTEN-induced kinase 1
PPE: Personal protective equipment PPP: Plant protection product
PPP: Plant protection product
PT: Product-type
RA: Risk assessment
Rab5A: Member RAS oncogene family
RAPD: Random amplified polymorphic DNA
REACH: Regulation, Evaluation, Authorisation and Restriction of Chemicals
RMMs: Risk mitigation measures
ROS: Reactive oxygen species
SAXS: Small angle X-ray scattering
SCCS: Scientific Committee on Consumer Safety
SCO1: Synthesis of cytochrome c oxidase 1
SDH: Succinate dehydrogenase
SDH A/B: Subunits A/B of succinate dehydrogenase (complex II)
SEM: Scanning electron microscopy
SLC25A3: Solute carrier family 25 member 3
SLC31A1: Solute carrier family 31 member 1
SLC31A2: Solute carrier family 31 member 2
SLC46A3: Solute carrier family 46 member 3
SML: Substance migration limit
SOD: Superoxide dismutase
SOD1: Superoxide dismutase 1
spICP-MS: Single particle Inductively coupled plasma mass spectrometry
STEAP: Six-transmembrane epithelial antigen of the prostate
STEM: Scanning transmission electron microscopy
TCA: Tricarboxylic acid
TEM: Transmission electron microscopy
TG: Test guideline
TOM: Translocator of the outer membrane
TR: Technical requirements
TRFC: Transferrin receptor
TTM: Tetrathiomolybdate
UL: Tolerable Upper Intake Level
ULK1: Unc-51 like autophagy activating kinase 1
w/o: Without
WB: Western blot
WST-1: Water-soluble tetrazolium 1
WST-8: Water-soluble tetrazolium 8
WT: Wild type
zVAD-fmk: Benzyloxycarbonyl-Val-Ala-Asp(OMe)-fluoromethylketone

## Declarations

### Disclaimer

This communication reflects only the authors' views. EFSA is not responsible for any use that may be made of the information it contains. AI support was used for language editing and linguistic polishing.
