# Supplementary material for: Copper oxide nanoparticles: from EU-regulatory landscape and mechanistic toxicity towards a cuproptosis-linked adverse outcome pathway
Source: Arch Toxicol. 2026 Jun 27;100(11):4855–906. doi: 10.1007/s00204-026-04465-1 (PMC13694461; doi:10.1007/s00204-026-04465-1)
Supplement: Supplementary file 1 [file 204_2026_4465_MOESM2_ESM.pdf]

Supplementary Information

Copper Oxide Nanoparticles:

From EU-Regulatory Landscape and Mechanistic Toxicity towards a Cuproptosis-Linked Adverse Outcome Pathway

Sophie Scholz[1,2], Julia Prinz[1], Linde Sevenants[3], Andrea Holzwarth[1], Birgit Mertens[3], Robert Pieper[1], Vera Ritz[1], Kirsten Schulz[1], Holger Sieg[1]*, Aswin Mangerich[2]*, Linda Böhmert[1]*

1 German Federal Institute for Risk Assessment, Max-Dohrn-Str. 8-10, 10589 Berlin, Germany
2 University of Potsdam, Institute of Nutritional Science, Nutritional Toxicology Group, Karl-Liebknecht-Str. 24-25, 14476 Potsdam, Germany
3 Sciensano (Belgian Institute for Health), J. Wytsmanstraat 14, 1050 Brussels, Belgium

E-Mail addresses and ORCID-ID:

Sophie Scholz	Sophie.Scholz@bfr.bund.de	0000-0003-3375-993X
Julia Prinz	Julia.Prinz@bfr.bund.de	
Linde Sevenants	Linde.Sevenants@sciensano.be	0009-0005-1681-2251
Andrea Holzwarth	Andrea.Holzwarth@bfr.bund.de	
Birgit Mertens	Birgit.Mertens@sciensano.be	0000-0001-8298-9104
Robert Pieper	Robert.Pieper@bfr.bund.de	0000-0002-0292-1482
Vera Ritz	Vera.Ritz@bfr.bund.de	
Kirsten Schulz	Kirsten.Schulz@bfr.bund.de	0000-0002-0090-761X
Holger Sieg	Holger.Sieg@bfr.bund.de	0000-0002-0593-0173
Aswin Mangerich	Mangerich@uni-potsdam.de	0000-0001-9742-2338
Linda Böhmert	Linda.Boehmert@bfr.bund.de	0000-0002-1153-2841

***Corresponding authors:**
Holger Sieg, German Federal Institute for Risk Assessment, Max-Dohrn-Str. 8-10, 10589 Berlin, Germany. Email: Holger.Sieg@bfr.bund.de
Aswin Mangerich, University of Potsdam, Institute of Nutritional Science, Nutritional Toxicology Group, Karl-Liebknecht-Str. 24-25, 14476 Potsdam, Germany. Email: mangerich@uni-potsdam.de
Linda Böhmert, German Federal Institute for Risk Assessment, Max-Dohrn-Str. 8-10, 10589 Berlin, Germany. Email: Linda.Boehmert@bfr.bund.de

Methods section for systematic review

Generation of dataset

A literature search was conducted on November 29, 2024 across four databases: Web of Science (Claviate), Embase, PubMed, and Google Scholar. These databases were selected based on their complementary coverage - Web of Science, Embase, and PubMed for their strong representation in toxicology-related journals, and Google Scholar its broad overall coverage. (Bramer et al., 2017, Gusenbauer, 2022) The search strategy comprised conceptional component: terms related to NPs, terms referring to copper oxides (CuO and Cu_2O) and related synonyms, and terms associated with toxicity. Relevant search terms were selected using MeSH terms from PubChem (PubChem, 2024a, PubChem, 2024b) and keyword clusters from a VOS viewer based on the network from Yan et al. 2023 for the search terms "nano" and "toxic". (Yan et al., 2023) To refine the search approach, we first analysed a preliminary dataset from August 28, 2023, which was generated using a comparable search string across all four databases but without exclusion terms. From this dataset, we derived suitable exclusion terms and evaluated their performance using the advanced search functionality in Endnote (Clarivate, Philadelphia, PA, USA). To ensure their specificity, we verified that these terms were absent in the "Any Field + PDF with Notes" search of relevant papers from the initial dataset (**Supplementary Tab. 1**). Based on this refinement, we constructed an updated search string and adapted it to the requirements and limitations of each database, including character limits, readable characters, and search operators (**Supplementary Tab. 2**).

Publications were compiled into a single library using Endnote (Clarivate, Philadelphia, PA, USA) and deduplicated automatically, followed by an additional manual deduplication step. All review articles were excluded. After deduplication, articles were screened for eligibility, defined as "any original research article that investigates toxicologically relevant aspects of CuO or Cu_2O NPs using suitable in vitro, in vivo and/or in silico methods, with a focus on mammals and birds". Screening was performed in a stepwise manner, beginning with the title, followed by the abstract, and in case of uncertainty the full text was reviewed. A flow chart summarising the article identification and screening process, prepared according to PRISMA guideline is shown in **supplementary Fig. 1**. (Page et al., 2021)

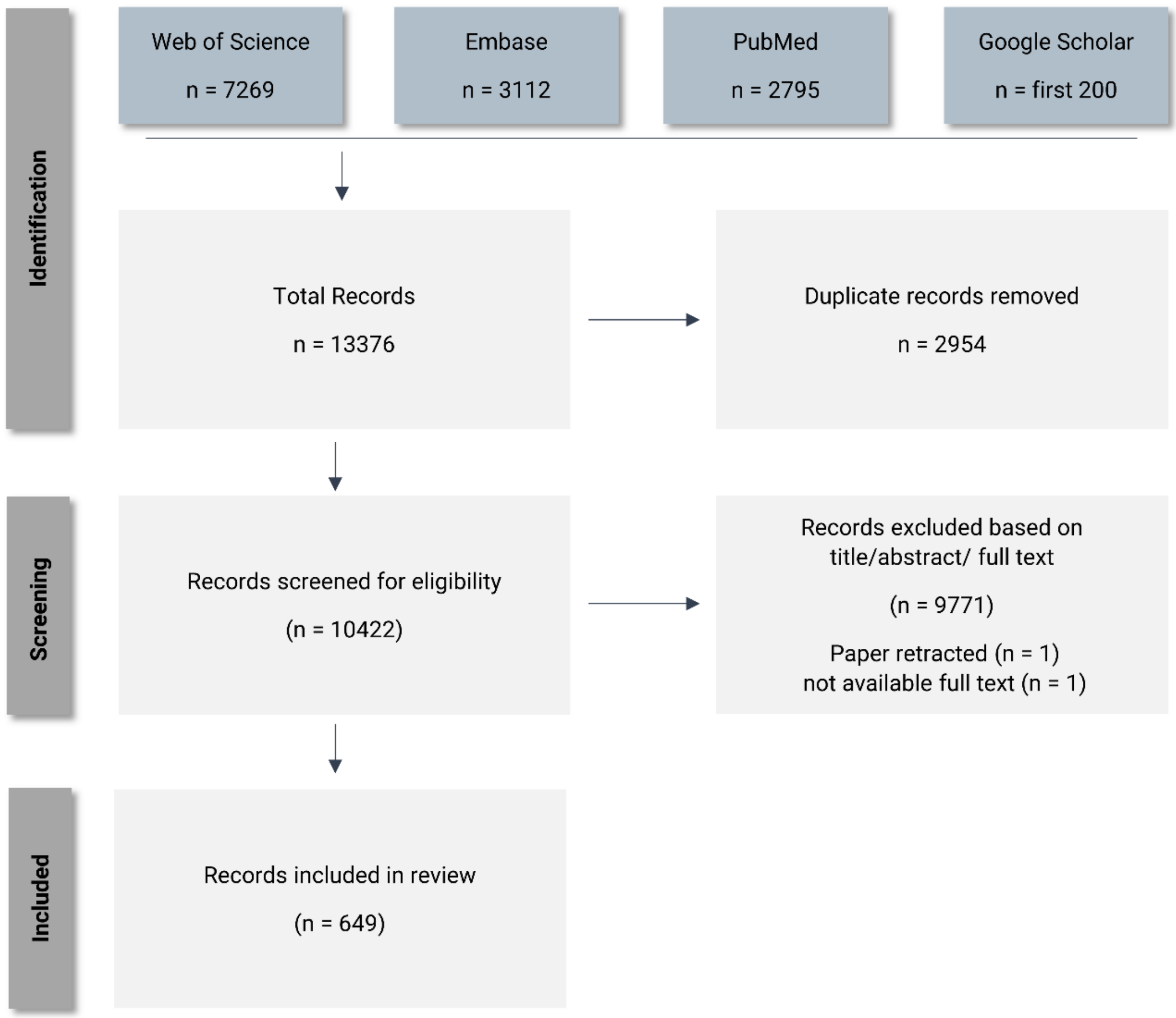

Supplementary Fig. 1 Identification and screening of literature for the systematic review. A literature search was performed in four different databases, selected for their comprehensive coverage of toxicological journals. After removing duplicate records, titles were screening for eligibility, followed by the abstract, and, when needed, full-text assessment. The Flow Chart is based on the PRISMA guideline by Page et al., 2021 (Page et al., 2021)

Analysis of dataset

The resulting dataset, comprising 649 articles, was analysed and systemically evaluated across several dimensions, briefly described below. Detailed categories and possible answer options used for the formal analysis are provided in supplementary Excel file. Most categories allowed multiple responses, for instance, an article assessing effects in both, cell line and animals, was categorised as in vitro and in vivo. Categories permitting multiple responses are marked with an asterix (*) in the supplementary Excel file. The analysis was carried out by three independent reviewers, each focusing on specific categories to ensure consistent assessment within their respective areas. Using Excel, all categorisations were documented and the graphs generated. Complete categorisations made for each article are provided in the supplementary Excel file.

All articles were broadly categorised by study type, indicating whether in vitro, in vivo and/or other methods were used. Additionally, each article was evaluated for the NPs applied and their main characteristics according to four categories: (i) copper oxidation state (CuO/Cu_2O/other), (ii) source of nanomaterial (purchased/self-synthesised), (iii) type of dispersion protocol and (iv) particle size

characterisation. Each article was also assessed for the inclusion of controls (ionic or bulk material) in at least one method.

For in vitro studies, the analysis also examined whether dosimetric considerations were addressed and whether particle size in cell culture media was characterised. Cell lines used in these studies were evaluated according to two categories: (i) species of origin and (ii) organ of origin. Toxicologically relevant assays and endpoints were grouped into 21 predefined categories, some representing single commonly used cytotoxicity assay (e.g., MTT or neural red uptake) and others representing broader endpoints, such as inflammation or oxidative stress, which can be assessed using various assays or methods. The complete list of considered endpoints is provided in the supplementary Excel file. Additionally, in-vitro articles were reviewed to determine whether they investigated cell death mechanisms (necrosis, apoptosis, autophagy, ferroptosis, cuproptosis, other) with and without the use of effectors (chemical inhibitors or inducers, knock-out, or knock-down approaches), performed OMICs analyses, or examined cellular uptake or transport of copper and/or particles.

We separately analysed the assays performed under the endpoint genotoxicity. These were specifically classified according to whether they followed using OECD test guideline (TG) methods (i.e., the in vitro Mammalian Cell Gene Mutation Test (TG 476) the in vitro Mammalian Chromosome Aberration Test (TG 473), the in vitro Mammalian Cell Micronucleus Test (TG 487)) or, the in vitro chromosome aberration assay, the in vitro micronucleus assay) or whether they employed other approaches (e.g., the in vitro Pig-a gene mutation assay, the in vitro transgenic gene mutation assays, the in vitro error-corrected next-generation sequencing, the in vitro comet assay, the γH2A.X assay, the ToxTracker). Genotoxicity was treated separately, because it is the only endpoint for which a substantial number of OECD TGs exist for in vitro testing. (Usmani et al., 2024)

For in vivo studies, articles were categorised by animal species and the route of exposure. Further, they were evaluated fore whether they assessed copper and/or particle uptake after oral exposure, performed omics analyses, or investigated genotoxicity. As with in vitro studies, genotoxicity in in vivo studies was examined with regard to the use of OECD TGs. Articles employing methods other than in vitro or in vivo approaches were reviewed for their assessment of artificial digestion and ion release of the NPs. Finally, articles focusing on in silico approaches or protein-NPs interactions were classified into a separate category.

In the following section, we present a comprehensive bibliometric overview of the CuO and Cu_2O NP literature identified using the methodology described above. A subsequent content-based evaluation of these publications was performed to derive meaningful insights relevant to our two remaining main research questions (ii) and (iii). The extracted experimental outcomes were systematically assessed and summarised in a structured table format.

Supplementary Tab.1 Exclusion terms. Identified using preliminary dataset from literature search in 2023.

Terms with no hits in preliminary dataset – suitable exclusion term		
• alloy formation	• laminar flow	• spectral engineering
• bacillus subtilis	• laminar pulsating flow	• surface modification
• bioaugmentation	• lattice Boltzmann simulation	• surface-plasmon-resonance
• carbon dots	• Li-ion batteries	• textile industry
• catalytic filter	• Lorentz forces	• thermal energy storage
• charge transfer	• nanofluid flow	• thermal performance
• convection flow	• nanofluid-cooled	• thin film
• dissipation energy	• natural water	• treatment of wastewater
• dual charge	• near infrared	• tribology film
• electrochemical performance	• nitrifying bacteria	• vertical rectangular duct
• entropy generation	• photocatalytic activity	• wastewater treatment
• Escherichia coli	• plant development	• zero-valent phase
• exfoliation technology	• plasmonic photocatalysts	• electrochemical Sensor
• fuel-cell	• pollution degradation	• geothermal
• gas detection	• quantum dots	• heteronanocrystals
• gas sensing	• solar cell	• hyperaccumulator
• graphene oxide	• solar collector	• nano-clay
• heat transfer	• solar energies	• superhydrophilic
• heated shallow cavity	• solar energy	• thermohydraulic
• high efficiency	• solar hydrogen production	• thermophoresis
• humic acid	• solar-driven	

Supplementary Tab. 2 Search strings for individual databases

Web of Science<br>https://www.webofscience.com/wos/woscc/advanced-search
((((AB=(nanoparticle*)) OR AB=(“nano particle*”)) OR AB=(nanomaterial*)) OR AB=(“nano material*”))<br>AND ((((((((((((ALL=("cuprous oxide")) OR ALL=("copper oxide")) OR ALL=("cupric oxide")) OR ALL=(CuO)) OR ALL=(Cu2O)) OR ALL=(“Copper(II) oxide”)) OR ALL=("Copper(I) oxide")) OR ALL=(thenorite)) OR ALL=(Cu(I)O)) OR ALL=(Cu(II)O)) OR ALL=(cuprite)) OR ALL=(perenox))<br>AND (((((((((((((((((((((((((((((((((((((((ALL=(toxicity)) OR ALL=(cytotoxicity)) OR ALL=("cell viability")) OR ALL=("cellular effect*")) OR ALL=(effect)) OR ALL=(impact)) OR ALL=(“oxidative stress”)) OR ALL=(stress)) OR ALL=(bioavailability)) OR ALL=(transport)) OR ALL=(uptake)) OR ALL=("Trojan horse")) OR ALL=(exposure)) OR ALL=(response)) OR ALL=(genotoxicity)) OR ALL=(inflammation)) OR ALL=(assay)) OR ALL=(accumulation)) OR ALL=(expression)) OR ALL=(inhibition)) OR ALL=(mechanism)) OR ALL=(“cell death”)) OR ALL=(apoptosis)) OR ALL=(autophagy)) OR ALL=(“in silico”)) OR ALL=("in vitro")) OR ALL=("in vivo")) OR ALL=(human)) OR ALL=(mammals)) OR ALL=(rat)) OR ALL=(mice)) OR ALL=(dog)) OR ALL=(pig)) OR ALL=(rabbit)) OR ALL=(animal)) OR ALL=(monkey)) OR ALL=(growth)) OR ALL=(porcine)) OR ALL=(cuproptosis))<br>NOT (((ALL=(“heat transfer”)) OR ALL=(“graphene oxide”)) OR ALL=(“photocatalytic activity”)) OR ALL=(“Escherichia coli”)) OR ALL=(“thermal performance”)) OR ALL=(“nanofluid flow”)) OR ALL=(“entropy generation”)) OR ALL=(“solar energy”)) OR ALL=(“charge transfer”)) OR ALL=(“thin film”)) OR ALL=(“gas sensing”)) OR ALL=(“surface-plasmon-resonance”)) OR ALL=(“quantum dots”)) OR ALL=(“wastewater treatment”)) OR ALL=(“thermal energy storage”)) OR ALL=(“laminar flow”)) OR ALL=(“electrochemical performance”)) OR ALL=(“surface modification”)) OR ALL=(“solar collector”)) OR ALL=(“high efficiency”)) OR ALL=(“bacillus subtilis”)) OR ALL=(“solar cell”)) OR ALL=(“near infrared”)) OR ALL=(“convection flow”)) OR ALL=(“humic acid”)) OR ALL=(“fuel-cell”)) OR ALL=(thermophoresis)) OR ALL=(“Lorentz forces”)) OR ALL=(“gas detection”)) OR ALL=(“textile industry”)) OR ALL=(“natural water”)) OR ALL=(geothermal)) OR ALL=(“plant development”)) OR ALL=(“Li-ion batteries”))OR ALL=(“carbon dots”)) OR ALL=(“solar-driven”)) OR ALL=(“treatment of wastewater”)) OR ALL=(“alloy formation”)) OR ALL=(thermohydraulic)) OR ALL=(hyperaccumulator)) OR ALL=(“nitrifying bacteria”)) OR ALL=(bioaugmentation)) OR ALL=(“dissipation energy”)) OR All=("Electrochemical Sensor")) Or all=("nano-clay"))
**Embase<br>https://www.embase.com/**
((nanoparticle) OR (nanoparticles) OR ("nano particle") OR (nano-particle) OR ("nano particles") OR (nano-particles) OR (nanomaterial) OR (nanomaterials) OR (nano-material) OR (nano-materials) OR ("nano material") OR ("nano materials"))<br>AND (("cuprous oxide") OR ("copper oxide") OR ("cupric oxide") OR (CuO) OR (Cu2O) OR (tenorite) OR (cuprite) OR (perenox))<br>AND ((toxicity) OR (cytotoxicity) OR ("cell viability") OR ("cellular effect*") OR (effect) OR (impact) OR (“oxidative stress”) OR (stress) OR (bioavailability) OR (transport) OR (uptake) OR ("Trojan horse") OR (exposure) OR (response) OR (genotoxicity) OR (inflammation) OR (assay) OR (accumulation) OR (expression) OR (inhibition) OR (mechanism) OR (“cell death”) OR (apoptosis) OR (autophagy) OR (“in silico”) OR ("in vitro") OR ("in vivo") OR (human) OR (mammals) OR (rat) OR (mice) OR (dog) OR (pig) OR (rabbit) OR (animal) OR (monkey) OR (growth) OR (porcine) OR (cuproptosis))<br>NOT ((“bacillus subtilis”) OR (“carbon dots”) OR (“charge transfer”) OR (“electrochemical performance”) OR (“entropy generation”) OR (“Escherichia coli”) OR (“exfoliation technology”) OR (“gas detection”) OR (“gas sensing”) OR (“graphene oxide”) OR (“heat transfer”) OR (“high efficiency”) OR (“humic acid”) OR (“laminar flow”) OR (“Li-ion batteries”) OR (“nitrifying bacteria”) OR (“photocatalytic activity”) OR (“plant development”) OR (“quantum dots”) OR (“solar cell”) OR (“solar energy”) OR (“surface modification”) OR (“textile industry”) OR (“thermal performance”) OR (“thin film”) OR (“treatment of wastewater”) OR (“wastewater treatment”) OR (“alloy formation”) OR (bioaugmentation) OR (“catalytic filter”) OR (“convection flow”) OR (“dissipation energy”) OR (“dual charge”) OR (“fuel-cell”) OR (geothermal) OR (“heated shallow cavity”) OR (heteronanocrystals) OR (hyperaccumulator) OR (“laminar pulsating flow”) OR (“lattice Boltzmann simulation”) OR (“Lorentz forces”) OR (“nanofluid flow”) OR (“nanofluid-cooled”) OR (“near infrared”) OR (“plasmonic photocatalysts”) OR (“pollution degradation”) OR (“solar collector”) OR (“solar energies”) OR (“solar hydrogen production”) OR (“solar-driven”) OR (“spectral engineering”) OR (superhydrophilic) OR (“surface-plasmon-resonance”) OR (“thermal energy storage”) OR (thermohydraulic) OR (thermophoresis) OR (“tribology film”) OR (“vertical rectangular duct”) OR (“zero-valent phase”) OR (“natural water”) OR (“Electrochemical Sensor”) OR (“nano-clay”))
**PubMed<br>https://pubmed.ncbi.nlm.nih.gov/**
((nanoparticle[Title/Abstract]) OR (nanoparticles[Title/Abstract]) OR ("nano particle"[Title/Abstract]) OR (nano-particle[Title/Abstract]) OR ("nano particles"[Title/Abstract]) OR (nano-particles[Title/Abstract]) OR (nanomaterial[Title/Abstract]) OR (nanomaterials[Title/Abstract]) OR (nano-material[Title/Abstract]) OR (nano-materials[Title/Abstract]) OR ("nano material"[Title/Abstract]) OR ("nano materials"[Title/Abstract]))<br>AND (("cuprous oxide") OR ("copper oxide") OR ("cupric oxide") OR (CuO) OR (Cu2O) OR (“Copper(II) oxide”) OR ("Copper(I) oxide") OR (tenorite) OR (Cu(I)O) OR (Cu(II)O) OR (cuprite) OR (perenox))<br>AND ((toxicity) OR (cytotoxicity) OR ("cell viability") OR ("cellular effect*") OR (effect) OR (impact) OR (“oxidative stress”) OR (stress) OR (bioavailability) OR (transport) OR (uptake) OR ("Trojan horse") OR (exposure) OR (response) OR (genotoxicity) OR (inflammation) OR (assay) OR (accumulation) OR (expression) OR (inhibition) OR (mechanism) OR (“cell death”) OR (apoptosis) OR (autophagy) OR (“in silico”) OR ("in vitro") OR ("in vivo") OR (human) OR (mammals) OR (rat) OR (mice) OR (dog) OR (pig) OR (rabbit) OR (animal) OR (monkey) OR (growth) OR (porcine) OR (cuproptosis))<br>NOT ((“bacillus subtilis”) OR (“carbon dots”) OR (“charge transfer”) OR (“electrochemical performance”) OR (“entropy generation”) OR (“Escherichia coli”) OR (“exfoliation technology”) OR (“gas detection”) OR (“gas sensing”) OR (“graphene oxide”) OR (“heat transfer”) OR (“high efficiency”) OR (“humic acid”) OR (“laminar flow”) OR (“Li-ion batteries”) OR (“nitrifying bacteria”) OR (“photocatalytic activity”) OR (“plant development”) OR (“quantum dots”) OR (“solar cell”) OR (“solar energy”) OR (“surface modification”) OR (“textile industry”) OR (“thermal performance”) OR (“thin film”) OR (“treatment of wastewater”) OR (“wastewater treatment”) OR (“alloy formation”) OR (bioaugmentation) OR (“catalytic filter”) OR (“convection flow”) OR (“dissipation energy”) OR (“dual charge”) OR (“fuel-cell”) OR (geothermal) OR (“heated shallow cavity”) OR (heteronanocrystals) OR (hyperaccumulator) OR (“laminar pulsating flow”) OR (“lattice Boltzmann simulation”) OR (“Lorentz forces”) OR (“nanofluid flow”) OR (“nanofluid-cooled”) OR (“near infrared”) OR (“plasmonic photocatalysts”) OR (“pollution degradation”) OR (“solar collector”) OR (“solar energies”) OR (“solar hydrogen production”) OR (“solar-

driven”) OR (“spectral engineering”) OR (superhydrophilic) OR (“surface-plasmon-resonance”) OR (“thermal energy storage”) OR (thermohydraulic) OR (thermophoresis) OR (“tribology film”) OR (“vertical rectangular duct”) OR (“zero-valent phase”) OR (“natural water”) OR (“Electrochemical Sensor”) OR (“nano-clay”))
Google Scholar *no exclusion terms were used due to character limitation of search engine<br>https://scholar.google.com/
nanoparticle|nanoparticles|“nano particle”|nano-particle|“nano particles”|nano-particles|nanomaterial|nanomaterials|nano-material|nano-materials|“nano material”|“nano materials” "cuprous oxide"|"copper oxide"|"cupric oxide"|CuO|Cu2O|“Copper(II) oxide”|"Copper(I) oxide"|tenorite|Cu(I)O|Cu(II)O|cuprite|perenox toxicity|cytotoxicity|"cell viability"|"cellular effect"|effect|impact|“oxidative stress”|stress|bioavailability|transport|uptake|"Trojan horse"|exposure|response|genotoxicity|inflammation|assay|accumulation|expression|inhibition|mechanism|“cell death”|apoptosis|autophagy|“in silico”|"in vitro"|"in vivo"|human|mammals|rat|mice|dog|pig|guinea pig|rabbit|hare|animal|monkey|growth|porcine|cuproptosis

Supplementary Tab. 3 Experimental evidence for prototypical stressors of adverse outcome pathway (AOP) 590 and evaluation of maturity and confidence level of the AOP. Overview of studies supporting [**A**] Wilson's disease and elesclomol as prototypical stressors [**B**] Key Event Relationships (KERs) and [**C**] essentiality of Key Events (KEs) based on tailored Bradford Hill criteria (Becker et al., 2015, Vinken et al., 2013, OECD, 2018) ATP: adenosine triphosphate; DLAT: dihydrolipoamide acetyltransferase; ETC: electron transport chain; FDX1: ferredoxin 1; FH: fumarate hydratase; HSP70: heat shock protein 70; Isca1: Iron-sulfur cluster assembly 1 homolog; IscS: Cysteine desulfurase; IscU/2: Iron-sulfur cluster assembly scaffold protein/2; MIE: molecular initiating event; SDH: succinate dehydrogenase; SDH A and B: subunits of succinate dehydrogenase (complex II); TCA: tricarboxylic acid

[A] Prototypical stressors	
Wilson's disease	• Patients with Wilson's disease (stressor 844) have altered abundance of TCA cycle metabolites (KE 2341). (Sarode et al., 2019)<br>• Wilson's disease models (stressor 844) lead to altered abundance of TCA cycle and glycolytic metabolites (KE 2341) *in vivo*. (Wooton-Kee et al., 2020)<br>• Patients with Wilson's disease (stressor 844) have lower enzymatic activity of complex I-IV (KE 178), and lower citrate synthase and aconitase activity (KE 2341). 4 of the 6 enzymes are Fe-S cluster proteins (KE 2342). (Gu et al., 2000)<br>• Wilson's disease model (stressor 844) leads to lower abundance of Fe-S cluster proteins (KE 2342) *in vivo*. (Tsvetkov et al., 2022)<br>• Wilson's disease model (stressor 844) leads to impaired Fe-S cluster assembly (KE 2342) *in vitro* and *in vivo*. (Du et al., 2023)
Elesclomol	• Elesclomol (stressor 843) leads to the formation of DLAT oligomers (KE 2340) and the reduction of Fe-S cluster proteins (KE 2342), induces changes in metabolites of the TCA cycle (KE 2341), as well as a decrease in cellular ATP levels (KE 40) *in vitro*. (Tsvetkov et al., 2022)<br>• Elesclomol (stressor 843) leads to the reduction of Fe-S cluster proteins (KE 2342) *in vitro*. (Barbi de Moura et al., 2012)<br>• Elesclomol (stressor 843) leads to the reduction of Fe-S cluster proteins (KE 2342), a decrease in cellular ATP levels (KE 40), the formation of DLAT oligomers (KE 2340) and a reduced activity of complex I and III (KE 178) *in vitro*. (Fu et al., 2024)
[B] Relationships between KEs (KERs)	
Dose-dependency	• Elesclomol (stressor 843) leads dose-dependently to higher intracellular copper levels (MIE 2339) *in vitro*. (Garza et al., 2022)<br>• Wilson's disease models (stressor 844) lead depending on severity to higher intracellular copper levels (MIE 2339) *in vivo*. (Lichtmannegger et al., 2016)<br>• DLAT aggregates (KE 2340) are formed at lower concentrations than proteotoxicity marker HSP70 (KE 889) is increased *in vitro* (western blot analysis in two cell lines treated with elesclomol). (Tsvetkov et al., 2022)<br>• Abundance of Fe-S cluster proteins (KE 2342) is reduced at lower concentrations than proteotoxicity marker HSP70 (KE 889) is increased *in vitro* (western blot analysis in two cell lines treated with elesclomol). (Tsvetkov et al., 2022)
Temporality	• Alpha-ketoglutarate levels (KE 2341) are reduced at earlier timepoints than ATP levels (KE 40) *in vitro* (metabolomic analysis after treatment with elesclomol in ABC1 cells), while no direct effects on

	ATP production capability was observed, but reduced maximal respiratory capacity, indicating a mechanism via ETC (KE 178) (seahorse assay after treatment with elesclomol in ABC1 cells). (Tsvetkov et al., 2022)
**[C] Essentiality of KEs in the pathway**	
KE 2342<br>Fe-S cluster assembly, impaired	• Impaired Fe-S cluster assembly (KE 2342) leads to lower complex I and II activity (KE 178) and lower ATP concentration (KE 40) *in vivo* (*Isca1* heterozygous knockout in rat). (Ling et al., 2021)<br>• Impaired Fe-S cluster assembly (KE 2342) leads to lower complex III activity (KE 178) and lower ATP production (KE 40) *in vivo* (IscS and IscU knockout in *T. brucerei*). (Smíd et al., 2006)<br>• Impaired Fe-S cluster assembly (KE 2342) leads to lower abundance of TCA cycle enzymes, and changes of several TCA cycle metabolites (KE 2341) *in vitro* (IscU2 knockdown in cell line). (Yang et al., 2025)
KE 2340<br>Oligomerisation of DLAT, increased	• Prevention of DLAT oligomer formation (KE 2340) leads to significantly reduced expression of the proteotoxic stress marker HSP70 (KE 889) *in vitro* (N-acetylcystein treatment in cell line). (Chen et al., 2025)
KE 178<br>Electron transport chain, disrupted	• Disrupted ETC (KE 178) leads to lower mitochondrial ATP production (KE 40) *in vitro* (*Sdha* and *b* knockout in cell line). (Gobelli et al., 2023)
KE 2341<br>TCA cycle, disrupted	• Disrupted TCA cycle (KE 2341) leads to lower ATP/ADP ratio (KE 40) and lower oxygen consumption rate (KE 178) *in vitro* (FH and SDH-inhibition in cell line). (Ryan et al., 2021)<br>• Disrupted TCA cycle (KE 2341) leads to lower oxygen consumption and reduced protein abundance of complex I, III and IV of the ETC (KE 178) *in vitro* (IDH2 knockdown in cell line). (Park et al., 2016)
KE 889<br>Proteostasis, impaired	No experimental evidence applicable as the downstream event is the AO 2343, increased cuproptosis. This cell death modality is by definition characterised by impaired proteostasis and effects on mitochondrial energy metabolism. (Tsvetkov et al., 2022)
KE 40<br>Mitochondrial ATP production, decreased	

References

BARBI DE MOURA, M., VINCENT, G., FAYEWICZ, S. L., BATEMAN, N. W., HOOD, B. L., SUN, M., SUHAN, J., DUENSING, S., YIN, Y. & SANDER, C. 2012. Mitochondrial respiration-an important therapeutic target in melanoma.

BECKER, R. A., ANKLEY, G. T., EDWARDS, S. W., KENNEDY, S. W., LINKOV, I., MEEK, B., SACHANA, M., SEGNER, H., VAN DER BURG, B. & VILLENEUVE, D. L. 2015. Increasing scientific confidence in adverse outcome pathways: application of tailored Bradford-Hill considerations for evaluating weight of evidence. *Regulatory Toxicology and Pharmacology,* 72**,** 514-537.

BRAMER, W. M., RETHLEFSEN, M. L., KLEIJNEN, J. & FRANCO, O. H. 2017. Optimal database combinations for literature searches in systematic reviews: a prospective exploratory study. *Systematic reviews,* 6**,** 1-12.

CHEN, Y., NAN, Y., XU, L., DAI, A., ORTEG, R. M. M., MA, M., ZENG, Y. & LI, J. 2025. Polystyrene nanoplastics exposure induces cognitive impairment in mice via induction of oxidative stress and ERK/MAPK-mediated neuronal cuproptosis. *Particle and Fibre Toxicology,* 22**,** 13.

DU, J., HUANG, Z., LI, Y., REN, X., ZHOU, C., LIU, R., ZHANG, P., LEI, G., LYU, J. & LI, J. 2023. Copper exerts cytotoxicity through inhibition of iron-sulfur cluster biogenesis on ISCA1/ISCA2/ISCU assembly proteins. *Free Radical Biology and Medicine,* 204**,** 359-373.

FU, D.-G., HE, J.-Z., MU, Q.-C., HUO, Y.-F., ZHANG, N.-M., ZHANG, L., HUA, S. & GAO, B.-Q. 2024. Inhibition of CTR1 expression improves hypoxia/reoxygenation-induced myoblast injury by blocking cuproptosis. *Biochemical and Biophysical Research Communications,* 735**,** 150804.

GARZA, N. M., ZULKIFLI, M. & GOHIL, V. M. 2022. Elesclomol elevates cellular and mitochondrial iron levels by delivering copper to the iron import machinery. *Journal of Biological Chemistry,* 298**,** 102139.

GOBELLI, D., SERRANO-LORENZO, P., ESTEBAN-AMO, M. J., SERNA, J., PÉREZ-GARCÍA, M. T., ORDUÑA, A., JOURDAIN, A. A., MARTÍN-CASANUEVA, M. Á., DE LA FUENTE, M. Á. & SIMARRO, M. 2023. The mitochondrial succinate dehydrogenase complex controls the STAT3-IL-10 pathway in inflammatory macrophages. *Iscience,* 26.

GU, M., COOPER, J., BUTLER, P., WALKER, A., MISTRY, P., DOOLEY, J. & SCHAPIRA, A. 2000. Oxidative-phosphorylation defects in liver of patients with Wilson's disease. *The Lancet,* 356**,** 469-474.

GUSENBAUER, M. 2022. Search where you will find most: Comparing the disciplinary coverage of 56 bibliographic databases. *Scientometrics,* 127**,** 2683-2745.

LICHTMANNEGGER, J., LEITZINGER, C., WIMMER, R., SCHMITT, S., SCHULZ, S., KABIRI, Y., EBERHAGEN, C., RIEDER, T., JANIK, D. & NEFF, F. 2016. Methanobactin reverses acute liver failure in a rat model of Wilson disease. *The Journal of clinical investigation,* 126**,** 2721-2735.

LING, Y., MA, J., QI, X., ZHANG, X., KONG, Q., GUAN, F., DONG, W., CHEN, W., GAO, S. & GAO, X. 2021. Novel rat model of multiple mitochondrial dysfunction syndromes (MMDS) complicated with cardiomyopathy. *Animal Models and Experimental Medicine,* 4**,** 381-390.

OECD 2018. Users' handbook supplement to the guidance document for developing and assessing AOPs. *Series on Testing & Assessment No. 233, Series on Adverse Outcome Pathways No. 1*.

PAGE, M. J., MCKENZIE, J. E., BOSSUYT, P. M., BOUTRON, I., HOFFMANN, T. C., MULROW, C. D., SHAMSEER, L., TETZLAFF, J. M., AKL, E. A. & BRENNAN, S. E. 2021. The PRISMA 2020 statement: an updated guideline for reporting systematic reviews. *bmj,* 372.

PARK, J.-B., NAGAR, H., CHOI, S., JUNG, S.-B., KIM, H.-W., KANG, S. K., LEE, J. W., LEE, J. H., PARK, J.-W. & IRANI, K. 2016. IDH2 deficiency impairs mitochondrial function in endothelial cells and endothelium-dependent vasomotor function. *Free Radical Biology and Medicine,* 94**,** 36-46.

PUBCHEM 2024a. National Center for Biotechnology Information (2024). PubChem Compound Summary for CID 164827, Cupric Oxide. Retrieved November 29, 2024

https://pubchem.ncbi.nlm.nih.gov/compound/cupric%20oxide.

PUBCHEM 2024b. National Center for Biotechnology Information (2024). PubChem Compound Summary for CID 10313194, Cuprous Oxide. Retrieved November 29, 2024

https://pubchem.ncbi.nlm.nih.gov/compound/Cuprous-Oxide.

RYAN, D. G., YANG, M., PRAG, H. A., BLANCO, G. R., NIKITOPOULOU, E., SEGARRA-MONDEJAR, M., POWELL, C. A., YOUNG, T., BURGER, N. & MILJKOVIC, J. L. 2021. Disruption of the TCA cycle reveals an ATF4-dependent integration of redox and amino acid metabolism. *Elife,* 10**,** e72593.

SARODE, G. V., KIM, K., KIEFFER, D. A., SHIBATA, N. M., LITWIN, T., CZLONKOWSKA, A. & MEDICI, V. 2019. Correction to: Metabolomics profiles of patients with Wilson disease reveal a distinct metabolic signature. *Metabolomics: Official Journal of the Metabolomic Society,* 16**,** 3-3.

SMÍD, O., HORÁKOVÁ, E., VILÍMOVÁ, V., HRDÝ, I., CAMMACK, R., HORVÁTH, A., LUKEŠ, J. & TACHEZY, J. 2006. Knock-downs of iron-sulfur cluster assembly proteins IscS and IscU down-regulate the active mitochondrion of procyclic Trypanosoma brucei. *Journal of Biological Chemistry,* 281**,** 28679-28686.

TSVETKOV, P., COY, S., PETROVA, B., DREISHPOON, M., VERMA, A., ABDUSAMAD, M., ROSSEN, J., JOESCH-COHEN, L., HUMEIDI, R. & SPANGLER, R. D. 2022. Copper induces cell death by targeting lipoylated TCA cycle proteins. *Science,* 375**,** 1254-1261.

USMANI, S. M., BREMER-HOFFMANN, S., CHEYNS, K., CUBADDA, F., DUMIT, V. I., ESCHER, S. E., FESSARD, V., GUTLEB, A. C., LÉGER, T. & LIU, Y. C. 2024. Review of new approach methodologies for application in risk assessment of nanoparticles in the food and feed sector: Status and challenges. *EFSA Supporting Publications,* 21**,** 8826E.

VINKEN, M., LANDESMANN, B., GOUMENOU, M., VINKEN, S., SHAH, I., JAESCHKE, H., WILLETT, C., WHELAN, M. & ROGIERS, V. 2013. Development of an adverse outcome pathway from drug-mediated bile salt export pump inhibition to cholestatic liver injury. *Toxicological Sciences,* 136**,** 97-106.

WOOTON-KEE, C. R., ROBERTSON, M., ZHOU, Y., DONG, B., SUN, Z., KIM, K. H., LIU, H., XU, Y., PUTLURI, N. & SAHA, P. 2020. Metabolic dysregulation in the Atp7b−/− Wilson's disease mouse model. *Proceedings of the National Academy of Sciences,* 117**,** 2076-2083.

YAN, X., YUE, T., WINKLER, D. A., YIN, Y., ZHU, H., JIANG, G. & YAN, B. 2023. Converting nanotoxicity data to information using artificial intelligence and simulation. *Chemical Reviews,* 123**,** 8575-8637.

YANG, X., LIANG, N., LIU, D., YAN, J., YANG, X., LV, J., XIAO, S., WEI, X., CHEN, X. & YANG, Z. 2025. Antioxidant capacity of the iron–sulfur cluster assembly protein IscU2 is mediated by aspartate metabolism to promote tumor survival. *Journal of Biological Chemistry,* 301.
